# Effects of *Withania somnifera (L.) Dunal* (Ashwagandha) on cognitive and physical function in adults: a systematic review and meta-analysis

**DOI:** 10.3389/fphar.2026.1799467

**Published:** 2026-05-11

**Authors:** XiaoLing Zhu, Qiang Zeng, Yuyu Lei, Danping Xu

**Affiliations:** 1 Department of Traditional Chinese Medicine, Qinghai University, Xi Ning, China; 2 Department of Traditional Chinese Medicine, The Eighth Affiliated Hospital, Sun Yat-sen University, Shen Zhen, China

**Keywords:** Withania somnifera, ashwagandha, cognitive function, physical function, meta-analysis

## Abstract

**Background:**

This study aimed to systematically review and evaluate the effects of *Withania somnifera (L.) Dunal* (Solanaceae) supplementation on cognitive and physical function in adults by meta-analysis.

**Methods:**

Following PRISMA guidelines, we searched PubMed, Web of Science, Cochrane Library, and Embase from inception to 1 February 2026. Ramdom control trials were included to evaluate the effects of Ashwagandha on cognitive function, musclestrength, testosterone, body weight, and body fat percentage by meta-analysis using Stata 14. A random-effects model was used to calculate standardized mean differences (SMD) with 95% confidence intervals. To assess the heterogeneity, sensitivity analyses and subgroup analyses were conducted. Methodological quality and risk of bias were assessed with RoB 2, evidence certainty for each outcome was evaluated with GRADE system, and botanical reporting was assessed with the ConPhyMP checklist.

**Results:**

Twenty studies with 1,249 participants were included. Ashwagandha significantly improved memory (SMD = 0.52; 95% CI: 0.27–0.78; P < 0.001; I^2^ = 29.7%), attention and processing speed (SMD = 0.29; 95% CI: 0.07–0.51; P = 0.009; I^2^ = 0%), and executive function (SMD = −0.42; 95% CI: −0.70 to −0.13; P = 0.004; I^2^ = 37.5%). Visuospatial ability improved after excluding one heterogeneous study (SMD = 0.39; 95% CI: 0.08–0.70; P = 0.013; I^2^ = 0%). Only one study assessed global cognition and reported significant improvement. For physical outcomes, Ashwagandha increased testosterone (SMD = 0.33; 95% CI: 0.13–0.54; P = 0.001; I^2^ = 20.8%) and muscle strength (SMD = 0.58; 95% CI: 0.12–1.04; P = 0.013; I^2^ = 56.9%). Subgroup analysis showed significant muscle strength gains in physically active individuals (SMD = 1.03; 95% CI: 0.56–1.49; P < 0.001; I^2^ = 0%) but not in untrained or special populations. No significant effects were observed on body weight or body fat percentage. Sensitivity analyses confirmed robustness, and most studies were rated as high or moderate quality.

**Conclusion:**

Ashwagandha supplementation significantly improved several aspects of cognitive function (including global cognitive, memory, attention and processing speed, executive function, and visuospatial ability), as well as serum testosterone and muscle strength in adults, but did not affect body weight or body fat percentage.

**Systematic Review Registration:**

https://www.crd.york.ac.uk/PROSPERO/view/CRD420261292996.

## 1 Introduction

*Withania somnifera (L.) Dunal* (Solanaceae), commonly known as Ashwagandha, is a traditional medicinal plant of the Solanaceae family used for thousands of years with a variety of health benefits (Paul et al., 2021). It is mainly distributed in dry tropical and subtropical areas such as India, the Middle East and Africa (Mikulska et al., 2023), in which India is the main planting center and using region. The plant is rich in a variety of bioactive metabolites, including steroidal lactones, alkaloids, flavonoids, saponins and glycosides (Dar et al., 2015).

Ashwagandha has been reported a variety of pharmacological activities, including anti-inflammation, anti-stress and neuroprotective effects. Studies have shown that its extracts and active metabolites, such as withanoside IV and its metabolite sominone, can promote axonal regeneration and synaptic reconstruction, thus improving memory and spatial cognitive ability. These characteristics suggest that it may have potential therapeutic value for neurodegenerative diseases such as Alzheimer’s disease and Parkinson’s disease (Kuboyama et al., 2014). In addition, drunken Ashwagandha can also regulate γ-aminobutyric acid (GABA) receptor activity and the hypothalamus-pituitary-adrenal (HPA) axis, reduce cortisol levels, relieve anxiety and stress reactions, and contribute to immune regulation and improve sleep quality (Guo and Rezaei, 2024). Clinical research further demonstrates that Ashwagandha can significantly improve memory, attention, and information-processing speed in patients with mild cognitive impairment (MCI) (Choudhary et al., 2017a).

Ashwagandha is often used to regulate mood, relieve anxiety and improve sleep quality. For example, Arumugam et al. (2024) evaluated nine randomized controlled trials by meta-analysis, and found that the scores of Perceived Stress Questionnaire (PSS), Anxiety Score (HAS) and serum cortisol levels were significantly reduced, indicating that it has a significant anti-pressure effect. In addition, Fatima et al. (2024) emphasized the efficacy of Ashwagandha in relieving anxiety and insomnia, especially in individuals with higher doses (>1 g/day) and more serious anxiety symptoms. A meta-analysis by Cheah et al. (2021) integrated the data of 400 participants, and the results showed that Ashwagandha extract could significantly improve the overall sleep quality of adults. In addition, it can also enhance physical functions such as muscle strength, muscle hypertrophy and aerobic exercise ability. A randomized controlled trial conducted by Verma et al. (2021) also yielded results similar to those above. A study (Rai and Mishra, 2025) involving 80 healthy subjects aged 18–45 years showed that, compared with the placebo group, the Ashwagandha-supplemented group significantly improved bench press and leg lift strength, arm circumference and chest circumference, thigh circumference, and maximum oxygen uptake (VO_2_max) (p < 0.05).

Despite the growing body of evidence, the effects of Ashwagandha on cognitive function remain controversial. Rai and Mishra (2025) reported that Ashwagandha extract can improve the cognitive abilities of patients with mild cognitive impairment, such as immediate memory, working memory, attention and information processing speed, but a recent study by Chengappa et al. (2013) failed to repeat these findings. Moreover, previous systematic reviews and meta-analyses have predominantly focused on individual outcomes such as stress, anxiety, sleep quality, or physical performance (Choudhary et al., 2017a; Arumugam et al., 2024; Fatima et al., 2024; Cheah et al., 2021), with limited synthesis of domain-specific cognitive functions. In addition, the variability in the composition of Ashwagandha preparations—particularly the inconsistent reporting of withanolides content—has raised concerns regarding the reproducibility and comparability of findings across studies, highlighting the need for a systematic evaluation that also assesses reporting quality.

Therefore, the present study aims to systematically evaluate the effects of Ashwagandha extracts on both cognitive function (including global cognitive, memory, attention, executive function, and visuospatial ability) and physical function (including muscle strength, testosterone levels, body weight, and body fat percentage) in adults, and to assess the reporting quality of botanical preparations in the included studies using the ConPhyMP checklist, providing a more integrated evidence base for the application of Ashwagandha in health promotion and clinical practice.

## 2 Methods

### 2.1 Study registration and protocol

This study followed the Preferred Reporting Items for Systematic Reviews and Meta-Analyses (PRISMA) guidelines (Moher et al., 2009). The PRISMA 2020 Checklist is available in the Supplementary Table S1. The prospective protocol for this systematic review was registered with the International Prospective Register of Systematic Reviews (PROSPERO), registration number CRD420261292996.

### 2.2 Information sources and search strategy

A systematic literature search was performed on 26 January 2026, and updated on 1 February 2026, using the following databases: PubMed, Web of Science, Cochrane Library, and Embase. Search terms for each database were derived from previous systematic reviews. Taking Web of Science as an example, the following query was used to search within titles, abstracts, and language fields: TS=((“Withania somnifera” OR “Ashwagandha”) AND (cognit* OR “executive function” OR memory OR attention OR testosterone OR “body weight” OR “body mass” OR BMI OR “body fat” OR “muscle strength”) AND (random* OR placebo OR “clinical trial”)) AND LA=(English)). The full search strategy is detailed in Supplementary Table S2.

Studies were excluded if the full text could not be obtained after attempting all available channels. Additionally, the reference lists of included trials were screened to identify potentially eligible studies. Title and abstract screening, as well as full-text assessment, were conducted independently by XZ and QZ to determine study eligibility. If disagreements arose between them, YL was consulted as a third reviewer to resolve discrepancies.

### 2.3 Eligibility criteria

To establish the eligibility criteria for this review, studies were required to meet the following PICOS (Participants, Intervention, Comparison, Outcomes, Study Design) framework (Spitzer et al., 1978): (1) Participants: populations encompassing individuals aged 18 years and older; (2) Intervention: Withania somnifera; (3) Comparison: placebo; (4) Cognitive outcomes covered domains such as global cognition, memory, attention and processing speed, executive function, and visuospatial ability, and this classification was applied consistently in the subsequent analyses. Hormonal outcomes primarily referred to serum testosterone levels. Muscle strength outcomes included measures of muscular strength, such as one-repetition maximum tests, while body composition outcomes included body weight and body fat percentage. (5) Study design: studies were included if they quantitatively evaluated the effects of Withania somnifera on at least one of the following outcomes: cognitive function, testosterone levels, muscle strength, body composition (body weight or body fat percentage).

### 2.4 Data collection process

Data from each study were independently extracted by two reviewers (XZ and QZ). The following information was collected: (1) surname of the first author; (2) year of publication; (3) country where the study was conducted; (4) type of study design; (5) number of participants and their age in both the intervention and control groups; (6) dosage and duration of the intervention; (7) relevant outcomes; (8) mean values, standard deviations, and sample sizes for each group before and after the intervention. These data were used to calculate changes in outcome measures between pre- and post-intervention. The calculations were performed as follows.

First, the calculation formula for the mean difference is as follows:

Mdiff=Mpost ‐Mpre

Mdiff denotes the raw mean difference, Mpost refers to the reported mean post-intervention, and Mpre refers to the reported mean pre-intervention (Cumpston et al., 2019).

The standard deviation of the mean difference (SDdiff) was calculated using the following formula:

$$\text{SDdiff}=\sqrt{{\text{SDpre}}^{2}+{\text{SDpost}}^{2}‐2r+\text{SDpre}\times \text{SDpost}}$$

SDdiff corresponds to the standard deviation of the mean difference, SDpre refers to the standard deviation in the pre-intervention period, and SDpost designates the standard deviation in the post-intervention phase (Cumpston et al., 2019). As the original studies did not report Pearson correlation coefficients (r), a value of r = 0.5, drawn from a previous meta-analysis (Chen et al., 2024), was used in the calculations.

For studies that reported only standard error (SE) or confidence intervals (CIs), these values were converted to standard deviation (SD) using the following formula:

$$\text{SD}=\text{SE}\times \sqrt{N}$$

$$\text{SD}=\sqrt{N}\times \frac{\text{CIupper}‐\text{CIlower}}{2t}$$

In this context, SE denotes standard error, SD represents standard deviation, N indicates the sample size of the group, CIupper refers to the upper limit of the confidence interval, CIlower signifies the lower limit of the confidence interval, and t corresponds to the t distribution with *N* – 1 degrees of freedom for the appropriate confidence interval (Cumpston et al., 2019).

### 2.5 The risk of bias of the included studies

To assess the impact of methodological limitations on our findings, we conducted sensitivity analysis excluding studies at high risk of bias. Risk of bias was assessed independently by two reviewers (XZ and QZ) using the Cochrane Collaboration’s Risk of Bias Tool 2 (RoB 2) (Ja et al., 2016). Five domains were evaluated (randomization process, deviations from intended interventions, missing outcome data, measurement of the outcome, and selection of the reported result), with each rated as “low risk of bias”, “some concerns”, or “high risk of bias” based on information from published reports, trial registrations, and Supplementary Material.

### 2.6 ConPhyMP assessment

The methodological quality of the included studies was assessed using the ConPhyMP tool, which provides standardized criteria for reporting the composition and processing of botanical preparations (Heinrich and Jalil, 2023). This tool evaluates key aspects, including botanical identification, extraction procedures, phytochemical characterization, and analytical methods. The assessment was performed independently by authors XZ and QZ, and detailed results are available in the Supplementary Material.

### 2.7 Data synthesis and analysis

All analyses were performed using Stata software (v14.0; StataCorp, College Station, TX, USA). Due to anticipated heterogeneity across studies, a random-effects model (DerSimonian and Laird method) was employed to pool effect estimates (Jackson et al., 2010). Calculate in difference of change value between the intervention group and the control group to quantify the result index. The combined mean (M) and standard deviation (SD) of two groups of variation values are included in the calculation. Because all interested outcome indicators are continuous variables and may be affected by sampling error deviation, Hedge’s g is chosen as the effect quantity indicator instead of Cohen’s d (Higgins et al., 2019). The combined effect of Hedge’s g is interpreted as small (0 ≤ g ≤ 0.50), medium (0.50 < g ≤ 0.80) or large (>0.80) (Cohen, 2013).

Heterogeneity among studies was evaluated using I statistics derived from the Cochran Q statistic (Higgins and Thompson, 2002). The I_2_ value represents the proportion of the total variation that can be attributed to the heterogeneity between studies. Values of about 25%, 50% and 75% are interpreted as low, medium, and high heterogeneity, respectively (Higgins et al., 2003). If articles (≥10) report a certain outcome indicator, a funnel plot is used for publication bias analysis (Sterne et al., 2011). For outcome indicators with high heterogeneity (I^2^ > 50%), sensitivity analyses were performed by excluding one study at a time from the model to explore sources of heterogeneity and assess the robustness of the pooled results, or subgroup analyses were conducted (Spineli and Pandis, 2020).

### 2.8 Certainty of evidence

Certainty of evidence was assessed using the GRADE framework. We considered risk of bias, inconsistency, indirectness, imprecision, and publication bias (Schünemann et al., 2019). Certainty was rated as high, moderate, low, or very low. One reviewer performed all GRADE assessments, which were verified by a second reviewer. Disagreements were resolved through discussion until a consensus was reached.

## 3 Results

### 3.1 Study selection

**FIGURE 1:**
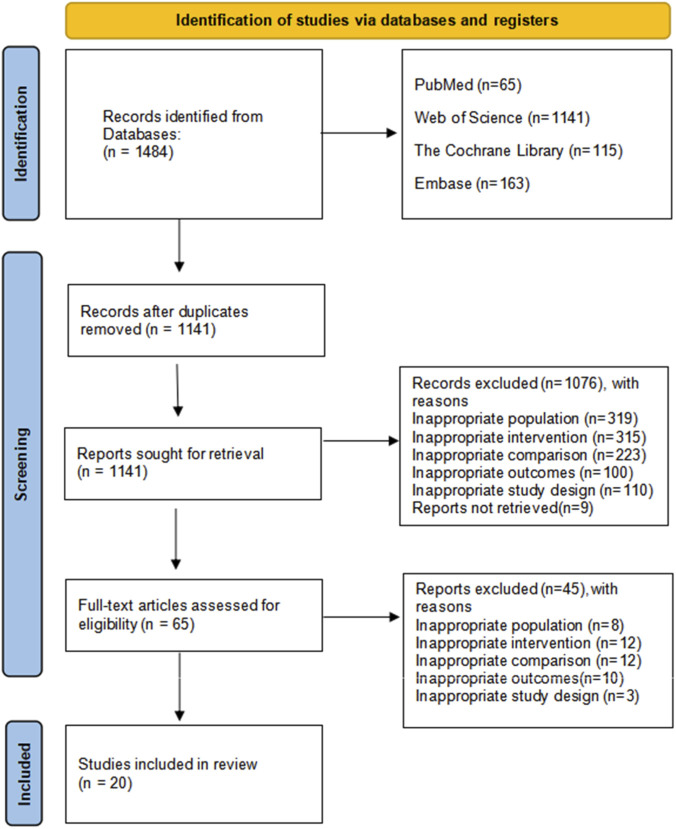
The low chart of the literature search and screening.

The electronic search retrieved 1,484 records (PubMed = 65; Web of Science = 1,141; Cochrane Library = 115; Embase = 163). After removing 343 duplicates, 1,141 records were screened. Based on title and abstract review, 1,076 records were excluded due to inappropriate population (n = 319), intervention (n = 315), comparison (n = 223), outcomes (n = 100), study design (n = 110), reports not retrieved (n = 9). The full texts of the remaining 65 articles were assessed for eligibility. Of these, 45 were excluded for similar reasons: inappropriate population (n = 8), intervention (n = 12), comparison (n = 12), outcomes (n = 10), and study design (n = 3). Consequently, 20 studies met the inclusion criteria and were included in the final meta-analysis. The study selection process is presented in Figure 1.

### 3.2 Study characteristics

Table 1 summarizes the characteristics of the 20 randomized controlled trials (RCTs) included in this meta-analysis. The studies, published between 2011 and 2025, originated from four countries: India (14 studies), the United States four studies), Australia (1 study), and Spain (1 study). The total pooled sample comprised 1,249 adult participants. Participants ranged in age from 18 to 75 years, with most studies enrolling healthy adults.

**TABLE 1:** Characteristics of the included studies.

Num	Study	Country	Design	Sample (A/B)	Age (years)	Intervention	Period	Outcomes	Safety classification	Simplified summary
1	Rai and Mishra (2025)	India	RCT	20/20	25–65	250 mg/day	8*W*	(1) WMS-III India; (2) MoCA; (3) MMSE; (4) Shepard Mental Rotation Task	No adverse events	No adverse events reported
2	Coope et al. (2025)	Spain	RCT	15/15	18–36	600 mg/day	4*W*	(1) Body Mass; (2) Body Fat	No adverse events	No adverse events reported
3	Leonard et al. (2024)	United States	RCT	30/30	18–60	225 mg/day	4*W*	(1) Word Recognition Test; (2) Choice Reaction Time Test; (3) Stroop Color–Word Test	Comparable between groups	No significant difference in AEs between groups
4	Verma et al. (2024)	India	RCT	37/36	18–45	600 mg/day (300 mg twice daily)	8*W*	(1) Body Mass; (2) Body Fat	No adverse events	No adverse events reported
5	Lopresti et al. (2019a)	India	RCT	30/30	18–65	240 mg/day	8*W*	(1) Testosterone	No adverse events	No adverse events reported
6	Lopresti et al. (2019b)	India	RCT	19/21	18–65	21 mg withanolide glycosides/day	16*W*	(1) Testosterone	Comparable between groups	No significant difference in AEs between groups
7	Choudhary et al. (2017a)	India	RCT	25/25	18–60	600 mg/day (300 mg twice daily)	8*W*	(1) WMS-III; (2) Attention and Information Processing Speed; (3) Executive Function; (4) Visuospatial Processing	Mild adverse events	A: 2 participants with mild AEs (giddiness, head heaviness, blurred vision, hyperacidity)
8	Wankhede et al. (2015)	India	RCT	25/25	18–50	600 mg/day (300 mg twice daily)	8*W*	(1) Testosterone	Comparable between groups	No significant difference in AEs between groups
9	Chengappa et al. (2013)	United States	RCT	29/24	18–65	250 mg/day (week 1), then 500 mg/day	8*W*	(1) Auditory Digit Span; (2) Flanker Test	Mild adverse events	A: 19 AEs B: 26 AEs; all were mild and transient
10	Smith et al. (2023)	Australia	RCT	52/56	40–75	400 mg/day (200 mg twice daily)	12*W*	(1) Testosterone	Comparable between groups	No significant difference in AEs between groups
11	Mutha et al. (2025)	India	RCT	47/46	30–50	600 mg/day (300 mg twice daily)	8*W*	(1) Testosterone	No adverse events	No adverse events reported
12	Choudhary et al. (2017b)	India	RCT	25/25	18–60	600 mg/day (300 mg twice daily)	8*W*	(1) Body Mass; (2) Body Fat	Mild adverse events	Only 2 participants with AEs (giddiness, heaviness of head, blurring of vision, and/or hyperacidity)
13	Remenapp et al. (2022)	India	RCT	55/59	18–54	225 mg/day or 400 mg/day	4*W*	(1) Reaction Time; (2) Executive Function	No adverse events	No adverse events reported
14	Kale et al. (2024)	India	RCT	55/59	30–75	600 mg/day (300 mg twice daily)	8*W*	(1) Episodic Memory; (2) Attention Accuracy; (3) COMPASS Location Learning Displacement	Mild adverse events	A: 3 participants with AEs (dizziness, bloating, frequent urination) B: 2 participants (dizziness, constipation)
15	Xing et al. (2022)	United States	RCT	25/25	18–59	400 mg single dose	7*D*	(1) Multitasking Test	No adverse events	No adverse events reported
16	Majeed et al. (2023)	India	RCT	18/25	21–54	500 mg extract 5 mg of 95% piperine	60*D*	(1) Delayed Matching Sample	No adverse events	No adverse events reported
17	Ziegenfuss et al. (2018)	United States	RCT	19/19	18–45	500 mg/day	12*W*	(1) Body Mass; (2) Body Fat	Mild adverse events	A: 3 participants with AEs (arthralgia, myalgia, abdominal pain); B: 2 participants (arthralgia)
18	Pingali et al. (2014)	India	RCT	21/25	20–35	500 mg/day (250 mg twice daily)	14*D*	(1) Digit Symbol Substitution Test; (2) Card Sorting Test	No adverse events	No adverse events reported
19	Ambiye et al. (2013)	India	RCT	21/25	22–40	675 mg/day (225 mg three times daily)	12*W*	(1) Testosterone	No adverse events	No adverse events reported
20	Gopal et al. (2021)	India	RCT	46/45	45–60	600 mg/day (300 mg twice daily)	8*W*	(1) Testosterone	Mild adverse events	A: 3 participants with AEs (abdominal discomfort, pain, nausea); B: 4 participants (abdominal discomfort, pain, insomnia, nausea)

RCT, randomized controlled trial; W, week; D, day; A, ashwagandha supplement; B, placebo.

The daily dosage of the intervention ranged from 225 mg to 675 mg Ashwagandha extracts, with 600 mg/day (typically administered as 300 mg twice daily) being the most commonly used dose. The intervention duration ranged from 7 days to 16 weeks, with 8 weeks being the most common.

Outcome measures were categorized into two primary domains: cognitive function and physical function. Cognitive outcomes were further classified into five domains, including global cognition, memory, attention and processing speed, executive function, and visuospatial ability, and this classification was applied consistently in the subsequent analyses. The most frequently employed comprehensive cognitive screening tool was the Mini-Mental State Examination (MMSE), while other assessments included the Wechsler Memory Scale (WMS-III), the Cambridge Neuropsychological Test Automated Battery (CANTAB), and the Computerised Mental Performance Assessment System (COMPASS) location learning displacement scores. Physical indicators encompassed serum testosterone levels, muscle strength as well as body weight and body fat percentage.

### 3.3 Characteristics and composition of ashwagandha preparations

Table 2 summarizes the characteristics and composition of the Ashwagandha preparations used in the included studies. Overall, most studies used Ashwagandha extracts derived from the root, while several studies employed extracts prepared from both root and leaf. In terms of formulation, the majority of interventions used standardized Ashwagandha extracts, although one study used a liposomal formulation. The content of withanolides varied substantially across studies. Several trials reported extracts standardized to approximately 5% total withanolides, which was the most commonly used specification. Some studies used higher standardized concentrations, such as ≥8% or ≥10% withanolides or withanolide glycosides, whereas others reported lower concentrations (e.g., 1.5% or 2.5% total withanolides). In certain preparations, additional characteristic constituents were provided, including withaferin A or the presence of oligosaccharides. However, several studies did not report detailed information on withanolides content. Overall, considerable variability existed across studies in terms of extract type, plant part used, and reported composition of Ashwagandha preparations, which may have implications for the reproducibility of the findings.

**TABLE 2:** Characteristics of the ashwagandha.

Num	Study	Preparation	Plant part	Withanolides content
1	Rai and Mishra (2025)	Ashwagandha extract standardized with Sominone	NR	Total withanolides ≥10%;sominone ≥2%
2	Coope et al. (2025)	Ashwagandha extract	Root	5% withanolides
3	Leonard et al. (2024)	Liposomal ashwagandha extract	Root and leaf	NR
4	Verma et al. (2024)	Ashwagandha extract	Root	>5% withanolides
5	Lopresti et al. (2019a)	Ashwagandha extract	Root and leaf	NR
6	Lopresti et al. (2019b)	Ashwagandha extract	Root and leaf	35% withanolide glycosides
7	Choudhary et al. (2017a)	Ashwagandha extract	Root	5% withanolides
8	Wankhede et al. (2015)	Ashwagandha extract	Root	5% withanolides
9	Chengappa et al. (2013)	Ashwagandha extract	Root and leaf	≥8% withanolides ≤2% withaferin-A ≥32% oligosaccharides
10	Smith et al. (2023)	Ashwagandha standardized extract	Root	1.5% total withanolides
11	Mutha et al. (2025)	Ashwagandha extract	Root	>5% withanolides
12	Choudhary et al. (2017b)	Ashwagandha extract	Root	5% withanolides
13	Remenapp et al. (2022)	Ashwagandha extract	Root and leaf	NR
14	Kale et al. (2024)	Ashwagandha extract	Root	>5% withanolides
15	Xing et al. (2022)	Ashwagandha extract	Root and leaf	NR
16	Majeed et al. (2023)	Ashwagandha standardized extract	Root	2.5% Total withanolides
17	Ziegenfuss et al. (2018)	Ashwagandha standardized extract	Root and leaf	NR
18	Pingali et al. (2014)	Withania somnifera extract	Root and leaf	≥10% withanolide glycosides, ≤0.5% withaferin-A, ≥32% oligosaccharides
19	Ambiye et al. (2013)	Ashwagandha extract	Root	≥5% withanolides
20	Gopal et al. (2021)	Ashwagandha extract	Root	>5% withanolides

NR: not reported in the original study.

### 3.4 ConPhyMP tool assessment results

The quality of botanical reporting was systematically assessed using the ConPhyMP reporting checklist, in accordance with best-practice guidelines for standardized reporting of botanical drug products. All 20 studies correctly reported the plant’s scientific name. Detailed botanical authentication (e.g., voucher specimen information and expert identification) was adequately described in 12 studies (60%). Extraction procedures, including solvent, temperature, and processing details, were fully reported in 12 studies (60%). Information on phytochemical characterization, including quantification of marker compounds, was provided in 13 studies (65%). Although manufacturer information was available in 19 studies (95%), batch numbers were reported in only two studies (10%). Certificates of analysis or third-party verification were mentioned in 15 studies (75%). Overall, the incomplete reporting of key botanical authentication, extraction procedures, and phytochemical characterization identified by the ConPhyMP assessment suggests a potential risk of non-reproducibility and limited comparability across the included studies, highlighting the need for standardized reporting in future trials. Detailed ConPhyMP assessment results for each individual study are provided in the Supplementary Material (ConPhyMP Tool Assessment).

### 3.5 Quality assessment

**FIGURE 2:**
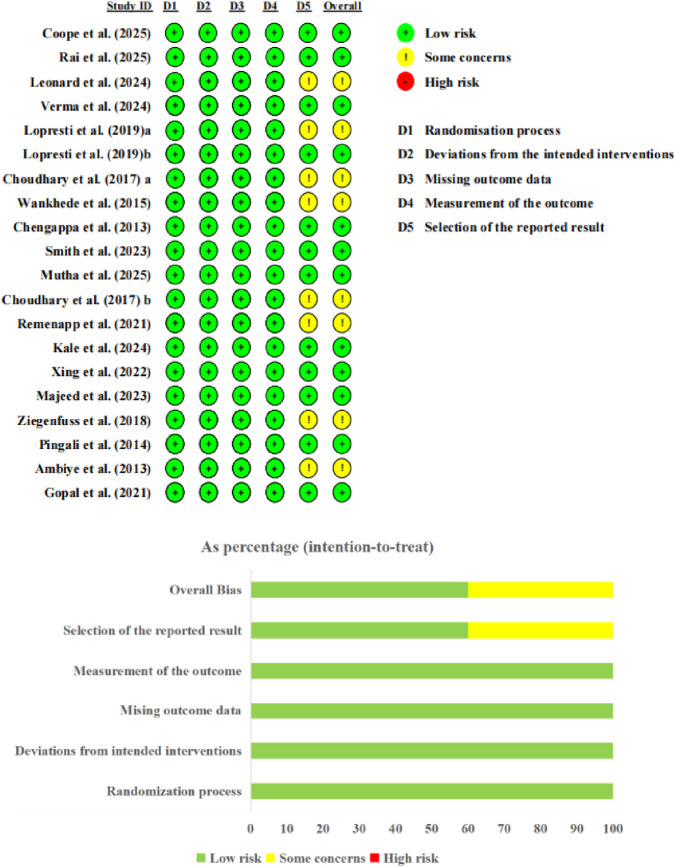
Risk of bias graph.

With respect to the RoB 2 assessment, most studies were rated as low risk for the domains of randomization process, deviations from intended interventions, missing outcome data, and measurement of the outcome, indicating generally acceptable methodological quality in these areas. For the domain of bias in selection of the reported result, 12 studies (60%) were rated as low risk, supported by the availability of prospectively registered protocols or consistency between reported outcomes and study methods, while eight studies (40%) were rated as “some concerns” due to the absence of prospective registration or insufficient information on pre-specified analysis plans, although no clear evidence of selective reporting was identified. Overall, 12 studies (60%) were judged as low risk and 8 (40%) as “some concerns,” with no study rated as high risk. The primary sources of uncertainty included the absence of prospectively registered protocols and insufficient reporting of key methodological details in some studies. Detailed assessments are presented in Figure 2.

### 3.6 GRADE evidence quality grading

A total of 10 outcome indicators were assessed. Among them, four indicators were rated as moderate quality (memory, attention and processing speed, body fat, body mass), four indicators were listed as low quality (executive function, visuospatial ability, muscle strength, testosterone levels), and two indicators were rated as very low quality (MoCA, MMSE). The results of the evidence quality assessment for cognitive function and physical function outcomes are presented in Supplementary Table S3.

### 3.7 Results of meta-analysis

#### 3.7.1 Effects of ashwagandha on cognitive function

Cognitive outcomes reported in the included studies covered multiple domains and were assessed using different validated instruments. For clarity, outcomes were categorized into five domains: global cognition, memory, attention and processing speed, executive function, and visuospatial ability. Meta-analyses were performed when at least two studies reported comparable outcomes.

##### 3.7.1.1 Global cognitive function

The Montreal Cognitive Assessment (MoCA) and Mini-Mental State Examination (MMSE) were used to evaluate global cognitive function. Only one studie (Rai and Mishra, 2025) involving 80 participants evaluated global cognitive function; therefore, a meta-analysis was not performed. Results were synthesized narratively. The study reported significant improvements in global cognition following Ashwagandha supplementation. The MoCA score increased significantly compared with the control group (standard mean difference [SMD] = 2.49, 95% CI: 1.64 to 3.33, P < 0.001, GRADE:Very low). Similarly, the MMSE score showed a significant improvement (SMD = 3.66, 95% CI: 2.61 to 4.70, P < 0.001, GRADE:Very low). Given that the evidence is derived from a single study, these findings should be interpreted with caution (Detailed forest plot results are provided in Supplementary Figures S1,S2 in the Supplementary Material).

##### 3.7.1.2 Memory

Six studies (Choudhary et al., 2017a; Rai and Mishra, 2025; Chengappa et al., 2013; Majeed et al., 2023; Kale et al., 2024; Leonard et al., 2024) involving 366 participants evaluated the effects of Ashwagandha on memory function using validated neuropsychological assessments, including the Wechsler Memory Scale (WMS-III) immediate memory subset, working memory tasks, word recall tests, and the Delayed Matching to Sample task. The pooled analysis demonstrated a statistically significant improvement in memory performance following Ashwagandha supplementation (standardized mean difference [SMD] = 0.52, 95% CI: 0.27 to 0.78, P < 0.001), with low heterogeneity across studies (I^2^ = 29.7%, GRADE: Moderate). These results indicate a moderate beneficial effect on memory-related outcomes (Supplementary Figure S3).

##### 3.7.1.3 Attention and processing speed

Five studies (Choudhary et al., 2017a; Kale et al., 2024; Leonard et al., 2024; Pingali et al., 2014; Remenapp et al., 2022) involving 327 participants assessed attention and processing speed using measures such as the Choice Reaction Time test, the Choice Discrimination test, and composite indices of attention. The pooled results showed a small but statistically significant improvement in favor of Ashwagandha supplementation (SMD = 0.29, 95% CI: 0.07 to 0.51, P = 0.009), with no observed heterogeneity (I^2^ = 0.0%, GRADE: Moderate). These findings suggest a consistent, albeit modest, effect on attention and processing speed (Supplementary Figure S4).

##### 3.7.1.4 Executive function

Six studies (Choudhary et al., 2017a; Chengappa et al., 2013; Majeed et al., 2023; Leonard et al., 2024; Pingali et al., 2014; Remenapp et al., 2022; Xing et al., 2022) involving 366 participants evaluated executive function using standardized neuropsychological tests, including the Stroop Color–Word Test, Flanker Test, Card Sorting Test, and Multitasking Test. Outcome measures in this domain were primarily reaction times or error rates, where lower values indicate better performance; therefore, negative SMD values favor the intervention group. The pooled analysis showed a statistically significant improvement in executive function following Ashwagandha supplementation (SMD = −0.42, 95% CI: −0.70 to −0.13, P = 0.004), with low-to-moderate heterogeneity (I^2^ = 37.5%, GRADE: Low) (Supplementary Figure S5).

##### 3.7.1.5 Visuospatial ability

Three studies (Choudhary et al., 2017a; Rai and Mishra, 2025; Kale et al., 2024) involving 204 participants assessed visuospatial ability using different tasks, including the Shepard mental rotation task and COMPASS-based spatial measures. The initial pooled analysis showed a large but non-significant effect (SMD = 1.40, 95% CI: −0.07 to 2.87, P = 0.062), with substantial heterogeneity (I^2^ = 94.5%, GRADE: Low) (Supplementary Figure S6).

Sensitivity analysis suggested that the study by Rai et al. may be a major source of this heterogeneity. After excluding this study, heterogeneity was reduced to 0% (I^2^ = 0%), and the pooled effect became statistically significant, with a small-to-moderate effect size (SMD = 0.39, 95% CI: 0.08 to 0.70, P = 0.013) (Supplementary Figure S7).

#### 3.7.2 Effects of ashwagandha on physical function

##### 3.7.2.1 Testosterone levels

Seven studies (Ambiye et al., 2013; Gopal et al., 2021; Lopresti et al., 2019a; Lopresti et al., 2019b; Mutha et al., 2025; Smith et al., 2023; Wankhede et al., 2015) involving 488 participants were included in the meta-analysis examining testosterone levels. The pooled results from the random-effects model indicated that Ashwagandha supplementation significantly increased testosterone levels compared with the control group (SMD = 0.33, 95% CI: 0.13–0.54, P = 0.001). Low heterogeneity was observed among the included studies (I^2^ = 20.8%, GRADE: Moderate) (Supplementary Figure S8).

##### 3.7.2.2 Muscle strength

Four studies (Wankhede et al., 2015; Coope et al., 2025; Verma et al., 2024; Ziegenfuss et al., 2018) involving 223 participants were included in the meta-analysis, comprising 114 participants in the Ashwagandha supplementation group and 109 in the placebo group. All studies assessed muscle strength and recovery-related outcomes. Using a random-effects model, the pooled analysis demonstrated that Ashwagandha supplementation significantly improved muscle strength compared with the control group (SMD = 0.58, 95% CI: 0.12–1.04, P = 0.013), with moderate heterogeneity across studies (I^2^ = 56.9%, GRADE: Low) (Supplementary Figure S9).

Subgroup analysis based on participants’ training status revealed that in two studies in physically active individuals, the pooled effect size was SMD = 1.03 (95% CI: 0.56–1.49, P < 0.001), with no heterogeneity within the subgroup (I^2^ = 0%). In contrast, in two studies in untrained or special populations, the pooled effect size was SMD = 0.23 (95% CI: −0.14–0.61, P = 0.219), with no heterogeneity observed (I^2^ = 0%) (Supplementary Figure S10).

##### 3.7.2.3 Body composition

Four studies (Coope et al., 2025; Verma et al., 2024; Ziegenfuss et al., 2018; Choudhary et al., 2017b) assessed body composition outcomes, including body fat percentage and body mass, in 191 participants each. The pooled analysis showed no significant effect of Ashwagandha supplementation on body fat percentage (SMD = −0.23, 95% CI: −0.50–0.05, P = 0.104; I^2^ = 0%, GRADE: Moderate). Similarly, no significant difference in body mass was observed between the Ashwagandha and control groups (SMD = 0.09, 95% CI: −0.20–0.37, P = 0.551; I^2^ = 0%, GRADE: Moderate) (Supplementary Figures S11-S12).

#### 3.7.3 Safety outcomes and adverse events

A total of 20 randomized controlled trials were included. Of these, six studies reported adverse events, while 12 explicitly stated that none occurred. Across all studies, reported adverse events were mild to moderate in severity. They were transient in nature and resolved either spontaneously or with minimal symptomatic treatment. No serious adverse events were reported. The most commonly reported adverse events involved the gastrointestinal system, such as abdominal discomfort, nausea, and bloating. These were followed by the nervous system (dizziness, headache, insomnia) and musculoskeletal system (arthralgia, myalgia). Other, less frequently reported events included rash or pruritus, urinary frequency, and fatigue. Only a small proportion of participants required dose adjustment or discontinuation due to adverse events. Overall, these findings indicate that Ashwagandha supplementation is generally well tolerated. However, the reporting of adverse events varied across studies. This variation may limit the accuracy of incidence estimates.

#### 3.7.4 Publication bias and sensitivity

Analysis Publication bias was planned to be assessed using funnel plots and Egger’s regression test for outcomes including at least 10 studies. However, none of the outcomes included sufficient studies (n ≥ 10) to allow a reliable assessment; therefore, publication bias was not formally evaluated.

Sensitivity analyses were conducted using a leave-one-out approach to explore the sources of heterogeneity. For visuospatial ability, sensitivity analysis indicated that one study (Rai et al.) contributed substantially to the observed heterogeneity. After excluding this study, heterogeneity was eliminated (I^2^ = 0%), and the pooled effect became statistically significant (SMD = 0.391, 95% CI: 0.081 to 0.700, P = 0.013). Overall, leave-one-out analyses showed that the pooled results were not materially altered by the exclusion of any single study, indicating the robustness of the findings. The detailed results of the sensitivity analyses are presented in Supplementary Figures S13–S21.

## 4 Discussion

This meta-study comprehensively evaluated the effects of Ashwagandha on cognitive and physical function of adults. Overall, the results supported the positive role of Ashwagandha in improving cognitive function, increasing serum testosterone levels, and increasing strength. However, its influence on body weight and body fat percentage did not reach statistical significance. These findings provided an important evidence basis for the application of Ashwagandha to enhance cognitive health, sports performance and related endocrine functions. According to the GRADE assessment, the certainty of evidence varied across outcomes, ranging from moderate to very low, suggesting that these findings should be interpreted with caution. Importantly, varying withanolides content was reported across the included studies, and several studies did not provide detailed compositional information. Moreover, differences in extraction methods and plant parts used were also observed. These factors may contribute to heterogeneity in the effect estimates, introduce potential bias, and affect the reproducibility of results.

### 4.1 Impact of ashwagandha on cognitive function

Previous meta-analysis showed that Ashwagandha could improve sleep, cortisol levels, and stress and anxiety (Arumugam et al., 2024; Cheah et al., 2021). However, few studies have explored the relationship between Ashwagandha supplements and cognitive function. In contrast to previous studies, the present meta-analysis evaluated cognitive outcomes across multiple domains, including global cognitive, memory, attention and processing speed, executive function and visuospatial ability. The results demonstrated that Ashwagandha supplementation significantly improved memory (moderate effect), attention and processing speed (weak effect), and executive function (moderate effect), whereas evidence for global cognition and visuospatial ability was limited or inconsistent. Notably, global cognitive function was assessed in only one study, and therefore no pooled analysis was performed, limiting the strength of inference. For visuospatial ability, the initial results showed substantial heterogeneity and were sensitive to the inclusion of individual studies; after excluding a study contributing to heterogeneity, a modest but statistically significant effect was observed.

It showed that Ashwagandha could effectively improve comprehensive performance global cognitive, memory, attention and processing speed, executive function and visuospatial ability. An 8-week randomized controlled trial involving 121 adults who reported cognitive and energy problems showed that taking 600 mg Ashwagandha extract daily could significantly improve the accuracy of situational memory, working memory and attention, and was superior to the placebo group in emotion, vitality and executive function (Kale et al., 2024). Another randomized controlled trial found that Ashwagandha extract can enhance working memory, help maintain attention and relieve mental fatigue at 3 h and 6 h after taking it (Xing et al., 2022). In addition, a 14-day study of 20 healthy men showed that Ashwagandha could significantly shorten the response time of many psychomotor tests (Pingali et al., 2014). Further research confirmed that Ashwagandha can significantly improve memory and recall, reduce error rates, relieve stress and improve sleep quality and mental health (Gopukumar et al., 2021). These domain-specific improvements suggested that Ashwagandha possibly exert selective effects on higher-order cognitive processes rather than producing a uniform enhancement across all cognitive domains.

### 4.2 Impact of ashwagandha on serum testosterone levels

This study showed that Ashwagandha could regulate endocrine and physiological function, especially significantly increased testosterone levels. An 8-week randomized controlled trial found that the serum testosterone levels of healthy men who supplemented with 600 mg of Ashwagandha extract daily, combined with resistance training, were significantly higher than those of the placebo group (Wankhede et al., 2015). Consistent with the literature, our meta-analysis showed that Ashwagandha can increase serum testosterone levels in healthy adults. The increase of testosterone level may partly contribute to the improvements in physical performance and muscle strength observed in this study.

### 4.3 Impact of ashwagandha on muscle strength

The meta-analysis revealed a moderate enhancement in muscle strength with Ashwagandha supplementation. A variety of previous studies have examined the effects of Ashwagandha supplementation on various aspects of muscle function in adults who have undergone resistance training. An eight-week, prospective, randomized, double-blind, placebo-controlled study (Verma et al., 2024) included 80 healthy subjects aged 18 to 45 showed that 300 mg of root extract (containing >5% withanolides) twice daily could significantly improve muscle strength and cardiopulmonary endurance. These findings were further supported by a follow-up study using 600 mg of the extract, which showed that it had positive effects on muscle volume, strength, and cardiopulmonary endurance after resistance training (Verma et al., 2024). It is worth noting that the heterogeneity of this part of the analysis is relatively high (I^2^ = 56.9%), which may be attributed to the differences in the evaluated strength indicators (such as grip strength, leg lift, and bench press), the training level of the subjects, and the supplementary dose and duration included in the study. More research is needed to further clarify the synergistic effects of Ashwagandha in the resistance-training population and to optimize its integration strategy across different training programs.

### 4.4 Impact of ashwagandha on body composition

The results of this meta-analysis showed that supplementation with Ashwagandha did not have a significantly direct impact on body weight or body fat percentage in adults during a relatively short intervention period. This finding is consistent with the pooled results, which showed no significant differences in body weight or body fat percentage between the intervention and control groups. This finding suggested that Ashwagandha extract had limited direct role in weight management as an independent intervention.

### 4.5 Impact of ashwagandha on safety

The findings of this systematic review indicate that Ashwagandha supplementation is generally well tolerated in clinical trials. The reported adverse events were all mild to moderate in severity, transient in nature, and resolved either spontaneously or with minimal symptomatic treatment, with no serious adverse events observed. This safety profile is consistent with previous literature. One study (Elgar, 2021) suggested that no serious adverse events or clinically significant changes in vital signs, hematological, or biochemical markers were observed in human trials; only mild to moderate transient adverse events such as gastrointestinal discomfort, dizziness, and drowsiness were reported. Ashwagandha is generally considered to be safe, with a few minor possible side-effects, although care should be taken alongside certain drugs.

### 4.6 Variability in botanical preparations and phytochemical composition

Another important consideration is the variability in the composition and quality of Ashwagandha preparations across studies. Several studies reported the content of withanolides, but the concentrations varied widely, and some lacked detailed phytochemical characterization. Given that withanolides are considered the primary bioactive metabolites, such variability may lead to differences in pharmacological effects. Furthermore, differences in extraction methods and plant parts (root vs. root and leaf) suggest that these preparations are not pharmacologically equivalent. This heterogeneity may contribute to inconsistent findings and limit the comparability and reproducibility of the results.

## 5 Limitations

This study has some limitations. First of all, although the overall sample size is sufficient, some outcome indicators (such as muscle strength, weight, and body fat percentage) are included in relatively few studies, which may affect the stability and generalizability of the estimation effect. Additionally, none of the outcome domains included sufficient studies (n ≥ 10) to allow a reliable assessment of publication bias, which may limit the robustness of the pooled estimates. Secondly, differences in the sources, standardized metabolites (especially the content of withanolides), dosage, and intervention duration of Ashwagandha extracts across studies may lead to heterogeneity in effects. In addition, differences in outcome assessment tools across studies may also contribute to heterogeneity in the results. Variability in botanical reporting quality, particularly incomplete reporting of withanolides content and extraction procedures, may compromise comparability across studies and introduce uncertainty in interpreting pooled effects. Third, most participants are healthy or sub-healthy, so its impact on clinical patients is unclear, which may limit the generalizability of the findings to populations with specific clinical conditions. Fourth, the safety of long-term use and the optimal dosage scheme need more data to further verify, as the duration of most included trials was relatively short. Finally, some original studies provide insufficient details on randomization, distribution concealment, and blinding, and may introduce bias, potentially affecting the overall reliability of the evidence.

Future research should focus on: 1) Conducting larger-sample, longer-term, multi-center randomized controlled trials using standardized, high-bioavailability extracts to confirm long-term benefits and safety; 2) Delving deeper into its potential value for specific populations such as the elderly, individuals with mild cognitive impairment, or those experiencing chronic stress; 3) Employing multi-omics technologies (e.g., metabolomics, transcriptomics) to further elucidate the specific molecular pathways underlying its cognitive-enhancing and endocrine-regulating effects; 4) Exploring its synergistic effects when combined with traditional physical exercise or cognitive training, to inform the development of multidimensional health promotion strategies.

## 6 Conclusion

In conclusion, Ashwagandha supplementation may improve several domains of cognitive function in adults, including global cognitive, memory, attention and processing speed, executive function, and visuospatial ability, as well as increase serum testosterone levels and muscle strength. However, no significant effects were observed on body weight or body fat percentage. Since there are differences in the composition of preparations used across studies and the withanolides content is not always clearly reported, the comparability and reproducibility of the findings remain limited. Furthermore, the overall certainty of evidence ranged from moderate to very low according to the GRADE assessment, indicating that these results should be interpreted with caution and that further high-quality, well-standardized trials are needed to confirm the effects. In addition, Ashwagandha is generally considered to be safe in most cases although it has been reported mild side effects, and caution should be taken regarding dosage and duration of use.

## Data Availability

The original contributions presented in the study are included in the article/Supplementary Material, further inquiries can be directed to the corresponding author.

## References

[B1] Ambiye V. R. Langade D. Dongre S. Aptikar P. Kulkarni M. Dongre A. (2013). Clinical evaluation of the spermatogenic activity of the root extract of ashwagandha (withania somnifera) in oligospermic males: a pilot study. Evid. Based Complement. Altern. Med. 2013, 571420. 10.1155/2013/571420 24371462 PMC3863556

[B2] Arumugam V. Vijayakumar V. Balakrishnan A. B Bhandari R. Boopalan D. Ponnurangam R. (2024). Effects of ashwagandha (withania somnifera) on stress and anxiety: a systematic review and meta-analysis. Explore 20, 103062. 10.1016/j.explore.2024.103062 39348746

[B3] Cheah K. L. Norhayati M. N. Husniati Yaacob L. Abdul Rahman R. (2021). Effect of ashwagandha (withania somnifera) extract on sleep: a systematic review and meta-analysis. PLOS One 16, e0257843. 10.1371/journal.pone.0257843 34559859 PMC8462692

[B4] Chen J. Xu T. Zhou J. Han B. Wu Q. Jin W. (2024). The superiority of schroth exercise combined brace treatment for mild-to-moderate adolescent idiopathic scoliosis: a systematic review and network meta-analysis. World Neurosurg. 186, 184–196.e9. 10.1016/j.wneu.2024.03.103 38531472

[B5] Chengappa K. N. R. Bowie C. R. Schlicht P. J. Fleet D. Brar J. S. Jindal R. D. (2013). Randomized placebo-controlled adjunctive study of an extract of withania somnifera for cognitive dysfunction in bipolar disorder. J. Diet. Suppl. 74, 1076–1083. 10.4088/JCP.13m08413 24330893

[B6] Choudhary D. Bhattacharyya S. Bose S. (2017a). Efficacy and safety of ashwagandha (*withania somnifera* (L.) *dunal*) root extract in improving memory and cognitive functions. J. Diet. Suppl. 14, 599–612. 10.1080/19390211.2017.1284970 28471731

[B7] Choudhary D. Bhattacharyya S. Joshi K. (2017b). Body weight management in adults under chronic stress through treatment with ashwagandha root extract: a double-blind, randomized, placebo-controlled trial. J. Evid-Based Complement. Altern. Med. 22, 96–106. 10.1177/2156587216641830 27055824 PMC5871210

[B8] Cohen J. (2013). Statistical power analysis for the behavioral sciences. 2nd ed. New York: Routledge, 567. 10.4324/9780203771587

[B9] Coope O. C. Reales Salguero A. Spurr T. Páez Calvente A. Domenech Farre A. Jordán Fisas E. (2025). Effects of root extract of ashwagandha on perception of recovery and muscle strength in female athletes. Eur. J. Sport Sci. 25, e12265. 10.1002/ejsc.12265 39954269 PMC11829707

[B10] Cumpston M. Li T. Page M. J. Chandler J. Welch V. A. Higgins J. P. (2019). Updated guidance for trusted systematic reviews: a new edition of the cochrane handbook for systematic reviews of interventions. Cochrane Database Syst. Rev. 10, ED000142. 10.1002/14651858.ED000142 31643080 PMC10284251

[B11] Dar N. J. Hamid A. Ahmad M. (2015). Pharmacologic overview of withania somnifera, the Indian ginseng. Cell Mol. Life Sci. 72, 4445–4460. 10.1007/s00018-015-2012-1 26306935 PMC11113996

[B12] Elgar K. (2021). Ashwagandha A review of clinical use and efficacy. Nutr. Med. J. 1 (1), 68–78.

[B13] Fatima K. Malik J. Muskan F. Raza G. Waseem A. Shahid H. (2024). Safety and efficacy of *withania somnifera* for anxiety and insomnia: systematic review and meta-analysis. Hum. Psychopharmacol. - Clin. Exp. 39, e2911. 10.1002/hup.2911 39083548

[B14] Gopal S. Ajgaonkar A. Kanchi P. Kaundinya A. Thakare V. Chauhan S. (2021). Effect of an ashwagandha (withania somnifera) root extract on climacteric symptoms in women during perimenopause: a randomized, double-blind, placebo-controlled study. J. Obstet. Gynaecol. Res. 47, 4414–4425. 10.1111/jog.15030 34553463

[B15] Gopukumar K. Thanawala S. Somepalli V. Rao T. S. S. Thamatam V. B. Chauhan S. (2021). Efficacy and safety of ashwagandha root extract on cognitive functions in healthy, stressed adults: a randomized, double-blind, placebo-controlled study. Evid-Based Complement. Altern. Med. 2021, 1–10. 10.1155/2021/8254344 34858513 PMC8632422

[B16] Guo S. Rezaei M. J. (2024). The benefits of ashwagandha (withania somnifera) supplements on brain function and sports performance. Front. Nutr. 11, 1439294. 10.3389/fnut.2024.1439294 39155932 PMC11327513

[B17] Heinrich M. Jalil B. (2023). From the CONSORT to the ConPhyMP statement and beyond—how to ascertain best practice. Front. Pharmacol. 14, 1338710. 10.3389/fphar.2023.1338710 38149050 PMC10750347

[B18] Higgins J. P. T. Thompson S. G. (2002). Quantifying heterogeneity in a meta-analysis. Stat. Med. 21, 1539–1558. 10.1002/sim.1186 12111919

[B19] Higgins J. P. T. Thomas J. Chandler J. Cumpston M. Li T. Page M. J. (2019). Cochrane handbook for systematic reviews of interventions. Wiley online books. 10.1002/9781119536604 PMC10284251 31643080

[B20] Higgins J. P. T. Thompson S. G. Deeks J. J. Altman D. G. (2003). Measuring inconsistency in meta-analyses. BMJ 327, 557–560. 10.1136/bmj.327.7414.557 12958120 PMC192859

[B21] Ja S. Ma H. Bc R. J S. Nd B. M V. (2016). ROBINS-I: a tool for assessing risk of bias in non-randomised studies of interventions. BMJ Clin. Res. Ed 355. 10.1136/bmj.i4919 PMC5062054 27733354

[B22] Jackson D. White I. R. Thompson S. G. (2010). Extending DerSimonian and laird’s methodology to perform multivariate random effects meta-analyses. Stat. Med. 29, 1282–1297. 10.1002/sim.3602 19408255

[B23] Kale S. Lopresti A. Suri R. Garg N. Langade D. (2024). Safety and efficacy of ashwagandha root extract on cognition, energy and mood problems in adults: prospective, randomized, placebo-controlled study. J. Psychoact. Drugs 58, 45–57. 10.1080/02791072.2024.2424279 39498904

[B24] Kuboyama T. Tohda C. Komatsu K. (2014). Effects of ashwagandha (roots of *withania somnifera*) on neurodegenerative diseases. Biol. Pharm. Bull. 37, 892–897. 10.1248/bpb.b14-00022 24882401

[B25] Leonard M. Dickerson B. Estes L. Gonzalez D. E. Jenkins V. Johnson S. (2024). Acute and repeated ashwagandha supplementation improves markers of cognitive function and mood. Nutrients 16, 1813. 10.3390/nu16121813 38931168 PMC11207027

[B26] Lopresti A. L. Drummond P. D. Smith S. J. (2019a). A randomized, double-blind, placebo-controlled, crossover study examining the hormonal and vitality effects of ashwagandha in aging, overweight males. Am. J. Men’s HEALTH 13, 1557988319835985. 10.1177/1557988319835985 30854916 PMC6438434

[B27] Lopresti A. L. Smith S. J. Malvi H. Kodgule R. (2019b). An investigation into the stress-relieving and pharmacological actions of an ashwagandha (withania somnifera) extract: a randomized, double-blind, placebo-controlled study. Med. (Baltimore) 98, e17186. 10.1097/MD.0000000000017186 31517876 PMC6750292

[B28] Majeed M. Nagabhushanam K. Mundkur L. (2023). A standardized ashwagandha root extract alleviates stress, anxiety, and improves quality of life in healthy adults by modulating stress hormones: results from a randomized, double-blind, placebo-controlled study. Med. (Baltimore) 102, e35521. 10.1097/MD.0000000000035521 37832082 PMC10578737

[B29] Mikulska P. Malinowska M. Ignacyk M. Szustowski P. Nowak J. Pesta K. (2023). Ashwagandha (withania somnifera)—current research on the health-promoting activities: a narrative review. Pharmaceutics 15, 1057. 10.3390/pharmaceutics15041057 37111543 PMC10147008

[B30] Moher D. Liberati A. Tetzlaff J. Altman D. G., and PRISMA Group (2009). Preferred reporting items for systematic reviews and meta-analyses: the PRISMA statement. PLoS Med. 6, e1000097. 10.1371/journal.pmed.1000097 19621072 PMC2707599

[B31] Mutha A. S. Mutha S. A. Tejuja A. H. Beldar A. S. Mulay A. M. Langade D. (2025). Efficacy and safety of eight-week therapy with ashwagandha root extract in improvement of sexual health in healthy men: findings of a prospective, randomized, double-blind, placebo-controlled study. J. Ayurveda Integr. Med. 16, 101155. 10.1016/j.jaim.2025.101155 40582043 PMC12266485

[B32] Paul S. Chakraborty S. Anand U. Dey S. Nandy S. Ghorai M. (2021). Withania somnifera (L.) dunal (ashwagandha): a comprehensive review on ethnopharmacology, pharmacotherapeutics, biomedicinal and toxicological aspects. Biomed. Pharmacother. 143, 112175. 10.1016/j.biopha.2021.112175 34649336

[B33] Pingali U. Pilli R. Fatima N. (2014). Effect of standardized aqueous extract of withania somniferaon tests of cognitive and psychomotor performance in healthy human participants. Pharmacogn. Res. 6, 12–18. 10.4103/0974-8490.122912 24497737 PMC3897003

[B34] Rai H. P. Mishra D. N. (2025). Effect of ashwagandha (*withania somnifera*) extract with sominone (somin-on^TM^) to improve memory in adults with mild cognitive impairment: a randomized, double-blind, placebo-controlled study. J. Psychopharmacol. 39, 350–363. 10.1177/02698811251324377 40099725

[B35] Remenapp A. Coyle K. Orange T. Lynch T. Hooper D. Hooper S. (2022). Efficacy of withania somnifera supplementation on adult’s cognition and mood. J. Ayurveda Integr. Med. 13, 100510. 10.1016/j.jaim.2021.08.003 34838432 PMC8728079

[B36] Schünemann H. J. Higgins J. P. Vist G. E. Glasziou P. Akl E. A. Skoetz N. (2019). Completing ‘Summary of findings’ tables and grading the certainty of the evidence. In Cochrane Handbook for Systematic Reviews of Interventions (eds Higgins J.P.T. Thomas J. Chandler J. Cumpston M. Li T. Page M.J. Welch V.A.). 10.1002/9781119536604.ch14

[B37] Smith S. J. Lopresti A. L. Fairchild T. J. (2023). Exploring the efficacy and safety of a novel standardized ashwagandha root extract (witholytin®) in adults experiencing high stress and fatigue in a randomized, double-blind, placebo-controlled trial. J. Psychopharmacol. 37, 1091–1104. 10.1177/02698811231200023 37740662 PMC10647917

[B38] Spineli L. M. Pandis N. (2020). Exploring heterogeneity in meta-analysis: meta-regression analysis. Am. J. Orthod. Dentofac. Orthop. Off. Publ. Am. Assoc. Orthod. Const. Soc. Am. Board Orthod. 158, 623–625. 10.1016/j.ajodo.2020.07.002 32988571

[B39] Spitzer R. L. Endicott J. Robins E. (1978). Research diagnostic criteria: rationale and reliability. Arch. Gen. Psychiatry 35, 773–782. 10.1001/archpsyc.1978.01770300115013 655775

[B40] Sterne J. A. C. Sutton A. J. Ioannidis J. P. A. Terrin N. Jones D. R. Lau J. (2011). Recommendations for examining and interpreting funnel plot asymmetry in meta-analyses of randomised controlled trials. BMJ 343, d4002. 10.1136/bmj.d4002 21784880

[B41] Verma N. Gupta S. K. Tiwari S. Mishra A. K. (2021). Safety of ashwagandha root extract: a randomized, placebo-controlled, study in healthy volunteers. Complement. Ther. Med. 57, 102642. 10.1016/j.ctim.2020.102642 33338583

[B42] Verma N. Gupta S. K. Patil S. Tiwari S. Mishra A. K. (2024). Effects of ashwagandha (withania somnifera) standardized root extract on physical endurance and VO2max in healthy adults performing resistance training: an eight- week, prospective, randomized, double-blind, placebo- controlled study. F1000Res 12, 1–23. 10.12688/f1000research.130932.2 38988644 PMC11234080

[B43] Wankhede S. Langade D. Joshi K. Sinha S. R. Bhattacharyya S. (2015). Examining the effect of withania somnifera supplementation on muscle strength and recovery: a randomized controlled trial. J. Int. Soc. Sports Nutr. 12, 43–53. 10.1186/s12970-015-0104-9 26609282 PMC4658772

[B44] Xing D. Yoo C. Gonzalez D. Jenkins V. Nottingham K. Dickerson B. (2022). Effects of acute ashwagandha ingestion on cognitive function. Int. J. Environ. Res. Public Health 19, 11852. 10.3390/ijerph191911852 36231152 PMC9565281

[B45] Ziegenfuss T. N. Kedia A. W. Sandrock J. E. Raub B. J. Kerksick C. M. Lopez H. L. (2018). Effects of an aqueous extract of withania somnifera on strength training adaptations and recovery: the STAR trial. Nutrients 10, 1807. 10.3390/nu10111807 30463324 PMC6266766

