# Supplementary material for: Effects of Withania somnifera (L.) Dunal (Ashwagandha) on cognitive and physical function in adults: a systematic review and meta-analysis
Source: Front Pharmacol. 2026 May 11;17:1799467. doi: 10.3389/fphar.2026.1799467 (PMC13199302; doi:10.3389/fphar.2026.1799467)
Supplement: Supplementary file 1 [file Supplementaryfile1.pdf]

Table S1.The PRISMAchecklist
Table S2. Complete Search Strategy Details for the Systematic Review and Meta-Analysis
Table S3. GRADE quality assessment

Figure S1. Forest plot of Montreal Cognitive Assessment (MoCA) score
Figure S2. Forest plot of Mini-Mental State Examination (MMSE) score
Figure S3. Forest plot of memory
Figure S4. Forest plot of attention and processing speed
Figure S5. Forest plot of executive function
Figure S6. Forest plot of visuospatial ability
Figure S7. Forest plot of visuospatial ability (excluding Rai et al.,2025)
Figure S8. Forest plot of Testosterone levels
Figure S9. Forest plot of muscle strength
Figure S10. Subgroup analysis for muscle strength by participants' training status revealed
Figure S11. Forest plot of body fat percentage
Figure S12. Forest plot of body mass

Figure S13. Sensitivity analysis – memory
Figure S14. Sensitivity analysis – attention and processing speed
Figure S15. Sensitivity analysis – executive function
Figure S16. Sensitivity analysis – visuospatial ability
Figure S17. Sensitivity analysis for visuospatial ability (excluding Rai et al.,2025)
Figure S18. Sensitivity analysis – testosterone levels
Figure S19. Sensitivity analysis – muscle strength
Figure S20. Sensitivity analysis – body fat percentage
Figure S21. Sensitivity analysis – body mass

Table S1.The PRISMAchecklist

Section and Topic	Item #	Checklist item	Location where item is reported
**TITLE**			
Title	1	Identify the report as a systematic review.	Title
**ABSTRACT**			
Abstract	2	See the PRISMA 2020 for Abstracts checklist.	Abstract
**INTRODUCTION**			
Rationale	3	Describe the rationale for the review in the context of existing knowledge.	Introduction
Objectives	4	Provide an explicit statement of the objective(s) or question(s) the review addresses.	Introduction
**METHODS**			
Eligibility criteria	5	Specify the inclusion and exclusion criteria for the review and how studies were grouped for the syntheses.	Methods
Information sources	6	Specify all databases, registers, websites, organisations, reference lists and other sources searched or consulted to identify studies. Specify the date when each source was last searched or consulted.	Methods
Search strategy	7	Present the full search strategies for all databases, registers and websites, including any filters and limits used.	Methods
Selection process	8	Specify the methods used to decide whether a study met the inclusion criteria of the review, including how many reviewers screened each record and each report retrieved, whether they worked independently, and if applicable, details of automation tools used in the process.	Methods
Data collection process	9	Specify the methods used to collect data from reports, including how many reviewers collected data from each report, whether they worked independently, any processes for obtaining or confirming data from study investigators, and if applicable, details of automation tools used in the process.	Methods
Data items	10a	List and define all outcomes for which data were sought. Specify whether all results that were compatible with each outcome domain in each study were sought (e.g. for all measures, time points, analyses), and if not, the methods used to decide which results to collect.	Methods
	10b	List and define all other variables for which data were sought (e.g. participant and intervention characteristics, funding sources). Describe any assumptions made about any missing or unclear information.	Methods
Study risk of bias assessment	11	Specify the methods used to assess risk of bias in the included studies, including details of the tool(s) used, how many reviewers assessed each study and whether they worked independently, and if applicable, details of automation tools used in the process.	Methods
Effect measures	12	Specify for each outcome the effect measure(s) (e.g. risk ratio, mean difference) used in the synthesis or presentation of results.	Methods
Synthesis methods	13a	Describe the processes used to decide which studies were eligible for each synthesis (e.g. tabulating the study intervention characteristics and comparing against the planned groups for each synthesis (item #5)).	Methods
	13b	Describe any methods required to prepare the data for presentation or synthesis, such as handling of missing summary statistics, or data conversions.	Methods
	13c	Describe any methods used to tabulate or visually display results of individual studies and syntheses.	Methods
	13d	Describe any methods used to synthesize results and provide a rationale for the choice(s). If meta-analysis was performed, describe the model(s), method(s) to identify the presence and extent of statistical heterogeneity, and software package(s) used.	Methods
	13e	Describe any methods used to explore possible causes of heterogeneity among study results (e.g. subgroup analysis, meta-regression).	Methods
	13f	Describe any sensitivity analyses conducted to assess robustness of the synthesized results.	Methods
Reporting bias assessment	14	Describe any methods used to assess risk of bias due to missing results in a synthesis (arising from reporting biases).	N/A
Certainty assessment	15	Describe any methods used to assess certainty (or confidence) in the body of evidence for an outcome.	Methods

Section and Topic	Item #	Checklist item	Location where item is reported
**RESULTS**			
Study selection	16a	Describe the results of the search and selection process, from the number of records identified in the search to the number of studies included in the review, ideally using a flow diagram.	Results
	16b	Cite studies that might appear to meet the inclusion criteria, but which were excluded, and explain why they were excluded.	Results
Study characteristics	17	Cite each included study and present its characteristics.	Results; Table1
Risk of bias in studies	18	Present assessments of risk of bias for each included study.	Results; Figure2
Results of individual studies	19	For all outcomes, present, for each study: (a) summary statistics for each group (where appropriate) and (b) an effect estimate and its precision (e.g. confidence/credible interval), ideally using structured tables or plots.	Results; Supplementary Figure S1-S21
Results of syntheses	20a	For each synthesis, briefly summarise the characteristics and risk of bias among contributing studies.	Results
	20b	Present results of all statistical syntheses conducted. If meta-analysis was done, present for each the summary estimate and its precision (e.g. confidence/credible interval) and measures of statistical heterogeneity. If comparing groups, describe the direction of the effect.	Results; Supplementary Figure S1-S12
	20c	Present results of all investigations of possible causes of heterogeneity among study results.	Results
	20d	Present results of all sensitivity analyses conducted to assess the robustness of the synthesized results.	Results; Supplementary Figure S13-S21
Reporting biases	21	Present assessments of risk of bias due to missing results (arising from reporting biases) for each synthesis assessed.	N/A
Certainty of evidence	22	Present assessments of certainty (or confidence) in the body of evidence for each outcome assessed.	Results; Supplementary Table S3
**DISCUSSION**			
Discussion	23a	Provide a general interpretation of the results in the context of other evidence.	Discussion
	23b	Discuss any limitations of the evidence included in the review.	Discussion
	23c	Discuss any limitations of the review processes used.	Discussion
	23d	Discuss implications of the results for practice, policy, and future research.	Discussion
**OTHER INFORMATION**			
Registration and protocol	24a	Provide registration information for the review, including register name and registration number, or state that the review was not registered.	Methods
	24b	Indicate where the review protocol can be accessed, or state that a protocol was not prepared.	Methods
	24c	Describe and explain any amendments to information provided at registration or in the protocol.	N/A
Support	25	Describe sources of financial or non-financial support for the review, and the role of the funders or sponsors in the review.	Funding
Competing interests	26	Declare any competing interests of review authors.	Conflict of interest
Availability of data, code and other materials	27	Report which of the following are publicly available and where they can be found: template data collection forms; data extracted from included studies; data used for all analyses; analytic code; any other materials used in the review.	N/A

Table S2. Complete Search Strategy Details for the Systematic Review and Meta-Analysis

Database	Search Strategy
PubMed 65	("Withania somnifera"[MeSH Terms] OR "Withania somnifera"[All Fields] OR "Ashwagandha"[All Fields]) AND ("Cognitive Dysfunction"[MeSH Terms] OR "Executive Function"[MeSH Terms] OR "Memory"[MeSH Terms] OR "Attention"[MeSH Terms] OR "cognit*"[All Fields] OR "executive function"[All Fields] OR "memory"[All Fields] OR "attention"[All Fields] OR "processing speed"[All Fields] OR "Testosterone"[MeSH Terms] OR "testosterone"[All Fields] OR "androgen*"[All Fields] OR "Body Weight"[MeSH Terms] OR "Body Mass Index"[MeSH Terms] OR "body weight"[All Fields] OR "body mass"[All Fields] OR "weight loss"[All Fields] OR "BMI"[All Fields] OR "Adipose Tissue"[MeSH Terms] OR "Body Composition"[MeSH Terms] OR "body fat"[All Fields] OR "adiposity"[All Fields] OR "body fat percentage"[All Fields] OR "fat mass"[All Fields] OR "lean mass"[All Fields] OR "Muscle Strength"[MeSH Terms] OR "Hand Strength"[MeSH Terms] OR "Physical Functional Performance"[MeSH Terms] OR "muscle strength"[All Fields] OR "handgrip strength"[All Fields] OR "grip strength"[All Fields] OR "muscular strength"[All Fields]) AND ("randomized controlled trial"[Publication Type] OR "controlled clinical trial"[Publication Type]) AND "English"[Language]
Web of Science 1141	TS=((“Withania somnifera” OR “Ashwagandha”) AND (cognit* OR “executive function” OR memory OR attention OR testosterone OR “body weight” OR “body mass” OR BMI OR “body fat” OR “muscle strength”) AND (random* OR placebo OR “clinical trial”)) AND LA=(English)
Cochrane Library 115	#1 (withania somnifera):ab,ti,kw<br>#2 (ashwagandha):ab,ti,kw<br>#3 #1 OR #2<br>#4 (cognit*):ab,ti,kw<br>#5 (executive function):ab,ti,kw<br>#6 (memory):ab,ti,kw<br>#7 (attention):ab,ti,kw<br>#8 (testosterone):ab,ti,kw<br>#9 (androgen*):ab,ti,kw<br>#10 (body weight):ab,ti,kw<br>#11 (body mass):ab,ti,kw<br>#12 (bmi):ab,ti,kw<br>#13 (body fat):ab,ti,kw<br>#14 (body composition):ab,ti,kw<br>#15 (muscle strength):ab,ti,kw<br>#16 (handgrip strength):ab,ti,kw<br>#17 #4 OR #5 OR #6 OR #7 OR #8 OR #9 OR #10 OR #11 OR #12 OR #13 OR #14 OR #15 OR #16<br>#18 (randomized controlled trial):ab,ti,kw<br>#19 (randomised controlled trial):ab,ti,kw<br>#20 (placebo):ab,ti,kw<br>#21 (rct):ab,ti,kw<br>#22 #18 OR #19 OR #20 OR #21

Database	Search Strategy
	#23 #3 AND #17 AND #22
Embase 163	1 'withania somnifera' OR 'withania somnifera' OR 'ashwagandha'<br>2 'cognition' OR 'executive function' OR 'memory' OR 'attention' OR 'testosterone' OR 'body weight' OR 'body mass' OR 'body composition' OR 'adipose tissue' OR 'muscle strength' OR 'cognit*' OR 'executive function' OR 'memory' OR 'attention' OR 'testosterone' OR 'body weight' OR 'bmi' OR 'body fat' OR 'muscle strength'<br>3 'randomized controlled trial' OR 'controlled clinical trial' OR 'randomized' OR 'placebo' OR 'clinical trial'<br>4 'english'<br>5 1 AND 2 AND 3 AND 4

Table S3. GRADE quality assessment

Outcome	K	Certainty of Evidence Assessment					Hedge's g [95% CI] *	GRADE†
		Risk of Bias	Inconsistency	Indirectness	Imprecision	Others		
**Cognitive Function**								
MoCA	K = 1	Not serious	Extremely serious	Not serious	Serious	Serious	2.49 [1.64–3.33]	⊕◯◯◯ Very low
MMSE	K = 1	Not serious	Extremely serious	Not serious	Serious	Serious	3.66 [2.61–4.70]	⊕◯◯◯ Very low
Memory	K = 6	Not serious	Not serious	Not serious	Not serious	Not serious	0.52[0.27–0.78]	⊕⊕⊕◯ Moderate
Attention and Processing Speed	K = 5	Not serious	Not serious	Not serious	Not serious	Not serious	0.29 [0.07–0.51]	⊕⊕⊕◯ Moderate
Executive Function	K = 6	Not serious	Not serious	Not serious	Not serious	Not serious	-0.42 [-0.70–0.13]	⊕⊕◯◯ Low
Visuospatial Ability	K = 3	Not serious	Not serious	Not serious	Not serious	Not serious	1.40 [-0.07–2.87]	⊕⊕◯◯ Low
**Physical Function**								
Testosterone	K =7	Serious	Not serious	Not serious	Not serious	Not serious	0.33[0.13–0.54]	⊕⊕⊕◯ Moderate

Muscle strength	K = 4	Not serious	Not serious	Not serious	Not serious	Not serious	0.58[0.12–1.04]	⊕⊕◯◯ Low
Body fat	K =4	Not serious	Not serious	Not serious	Not serious	Not serious	-0.23 [-0.50–0.05]	⊕⊕⊕◯ Moderate
Body mass	K =4	Not serious	Not serious	Not serious	Not serious	Not serious	0.09 [-0.20–0.37]	⊕⊕⊕◯ Moderate

Notes: K**:** the total number of effects included in the pooled effect size; ***Publication bias***, represented by Egger test $p < 0.05$.

***:** The effect size (*Hedges'g*) was significant ($p < 0.05$).

† GRADE Criteria for Certainty of Evidence:

High: Very confident in the estimated effect.
Moderate: Moderately confident in the estimated effect.
Low: Limited confidence in the estimated effect.
Very low: Very limited confidence in the estimated effect.

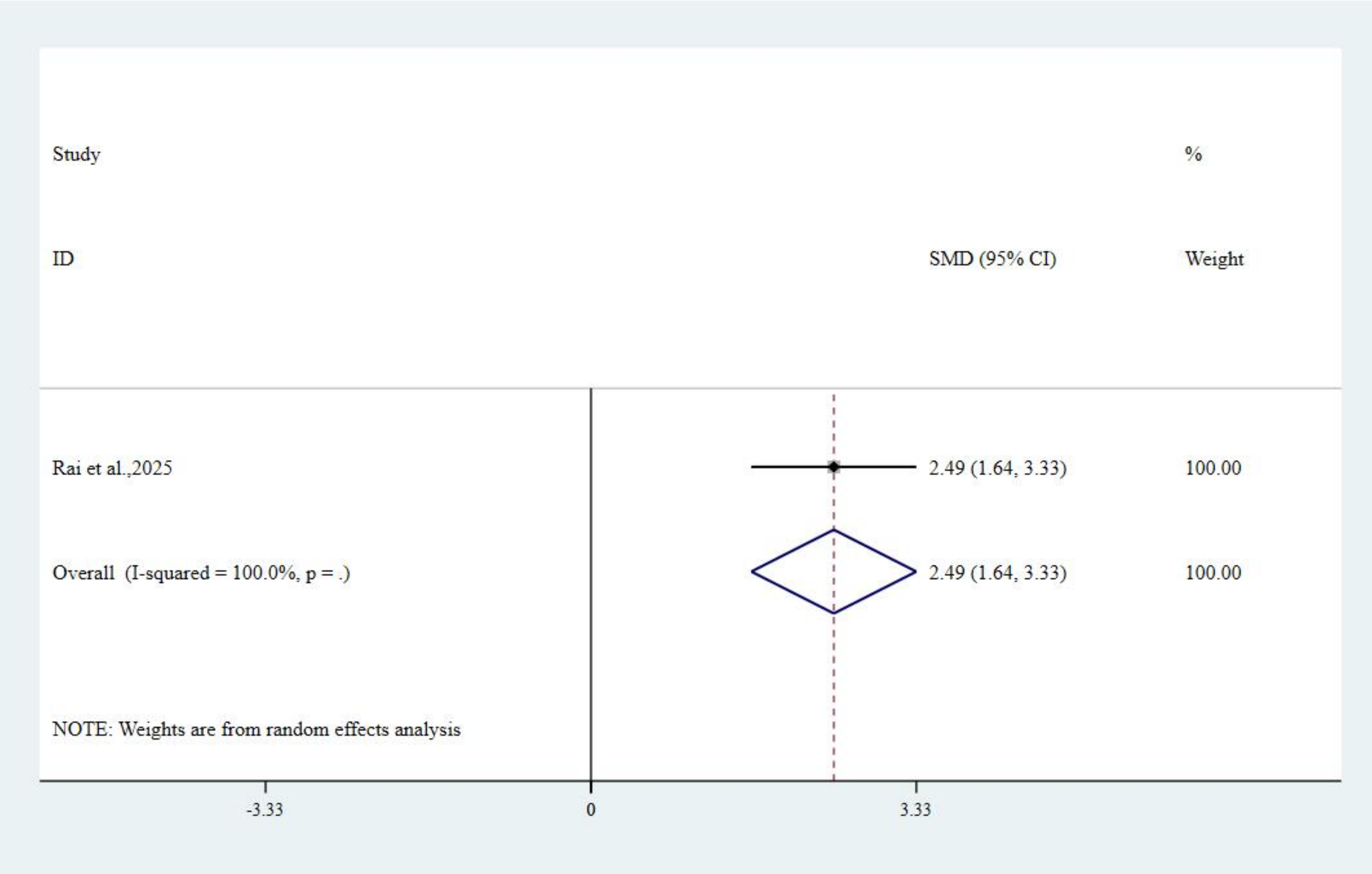

Figure S1. Forest plot of Montreal Cognitive Assessment (MoCA) score

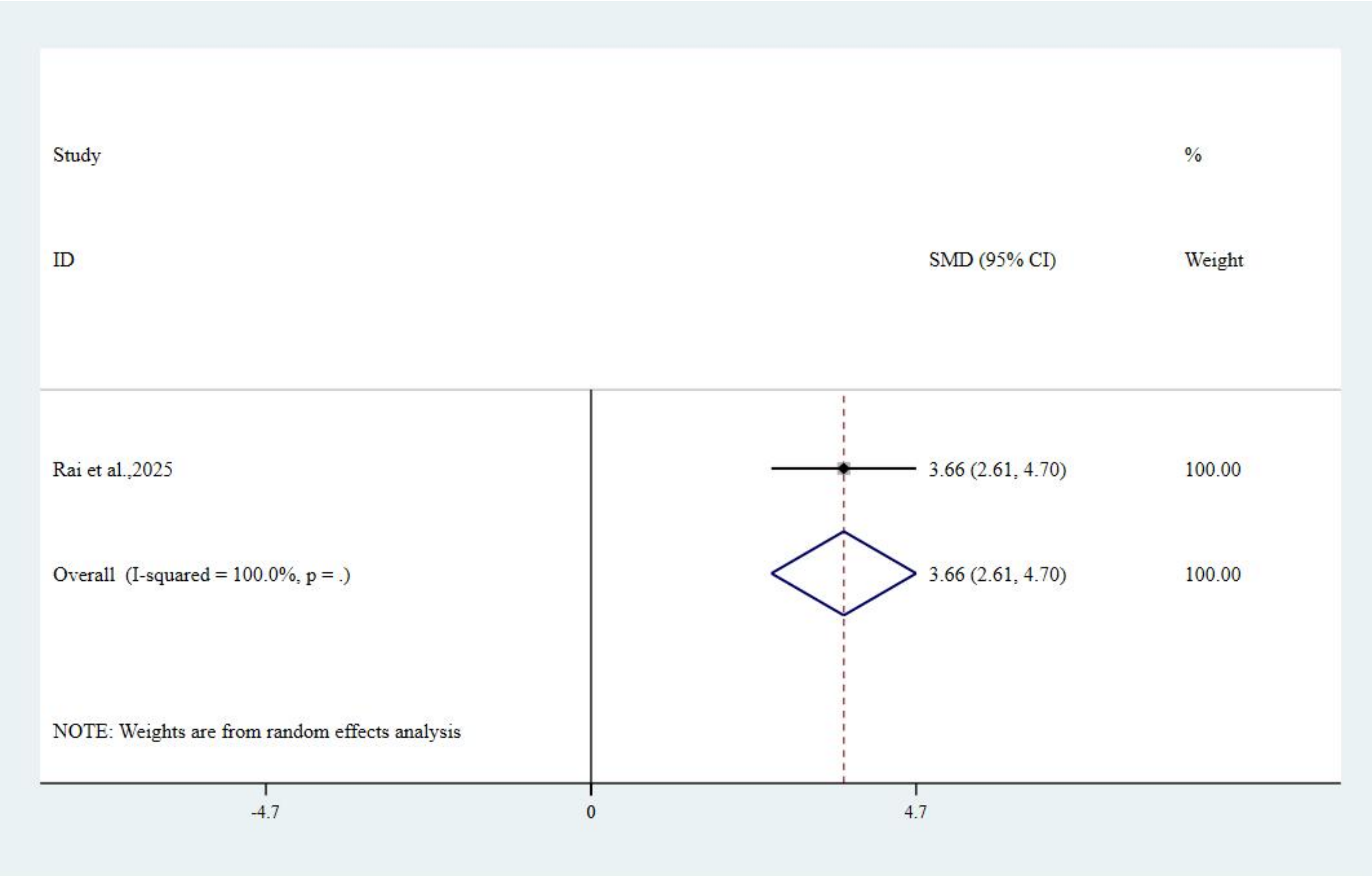

Figure S2. Forest plot of Mini-Mental State Examination (MMSE) score

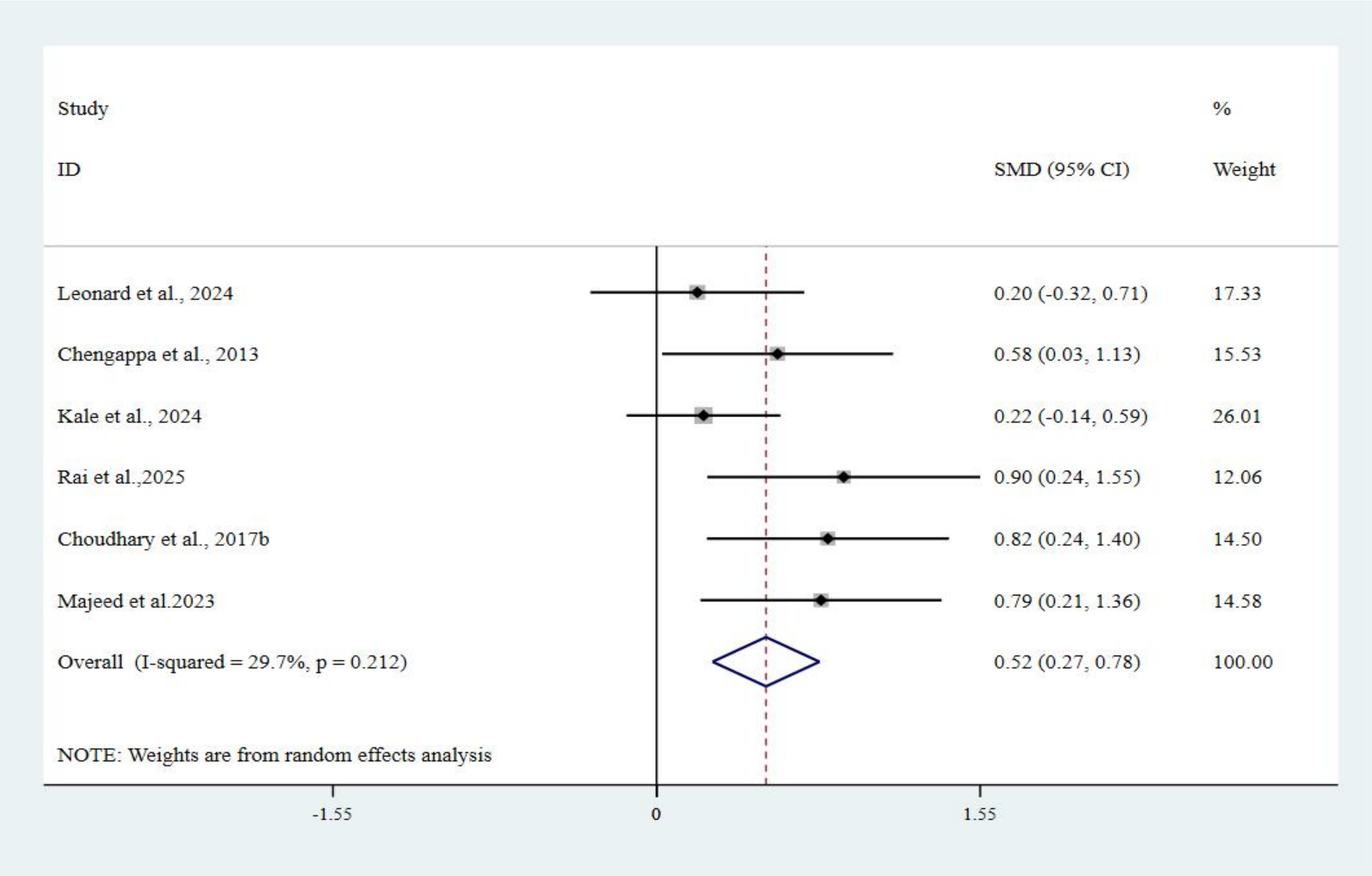

Figure S3. Forest plot of memory

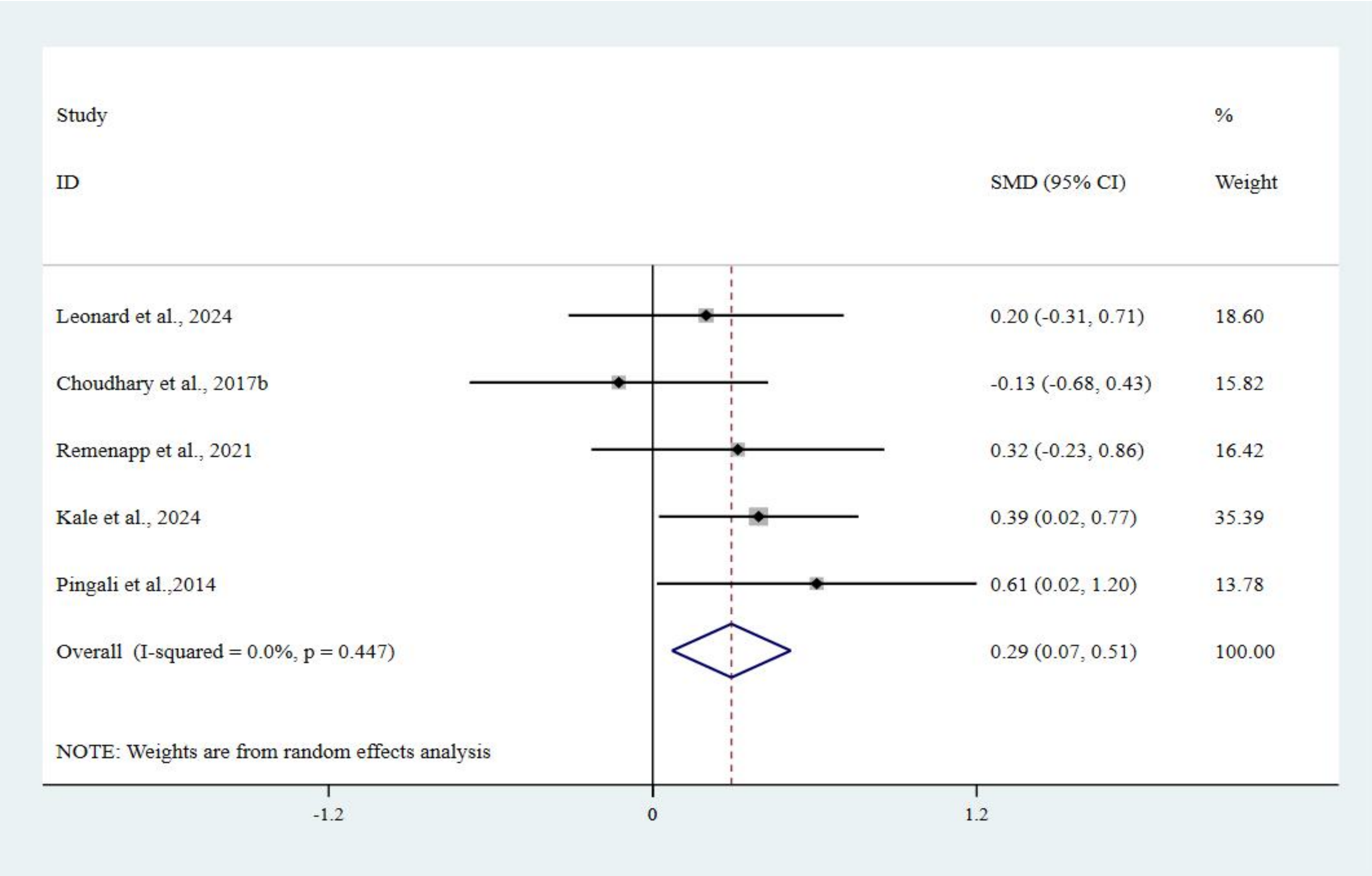

Figure S4. Forest plot of attention and processing speed

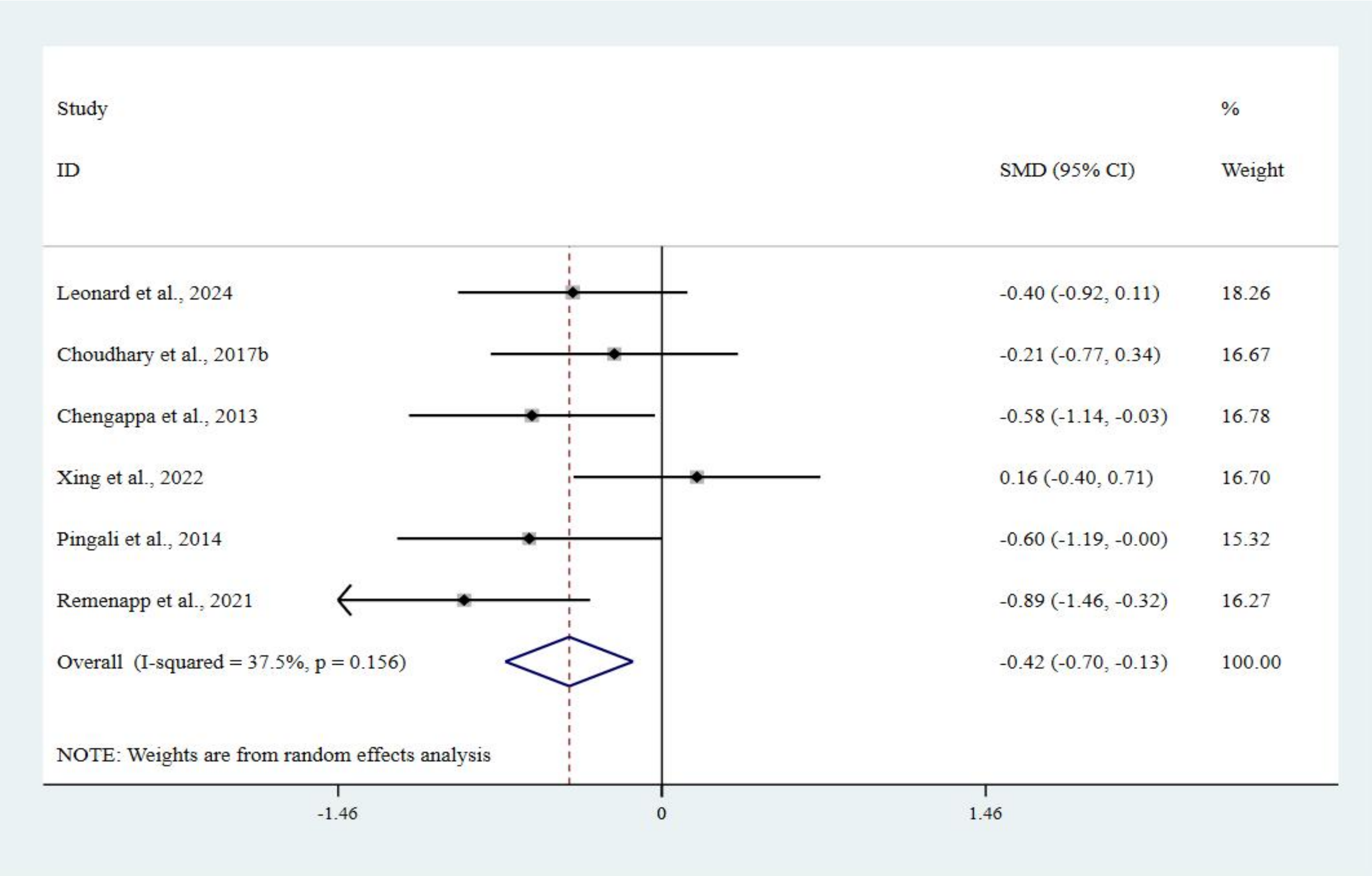

Figure S5. Forest plot of executive function

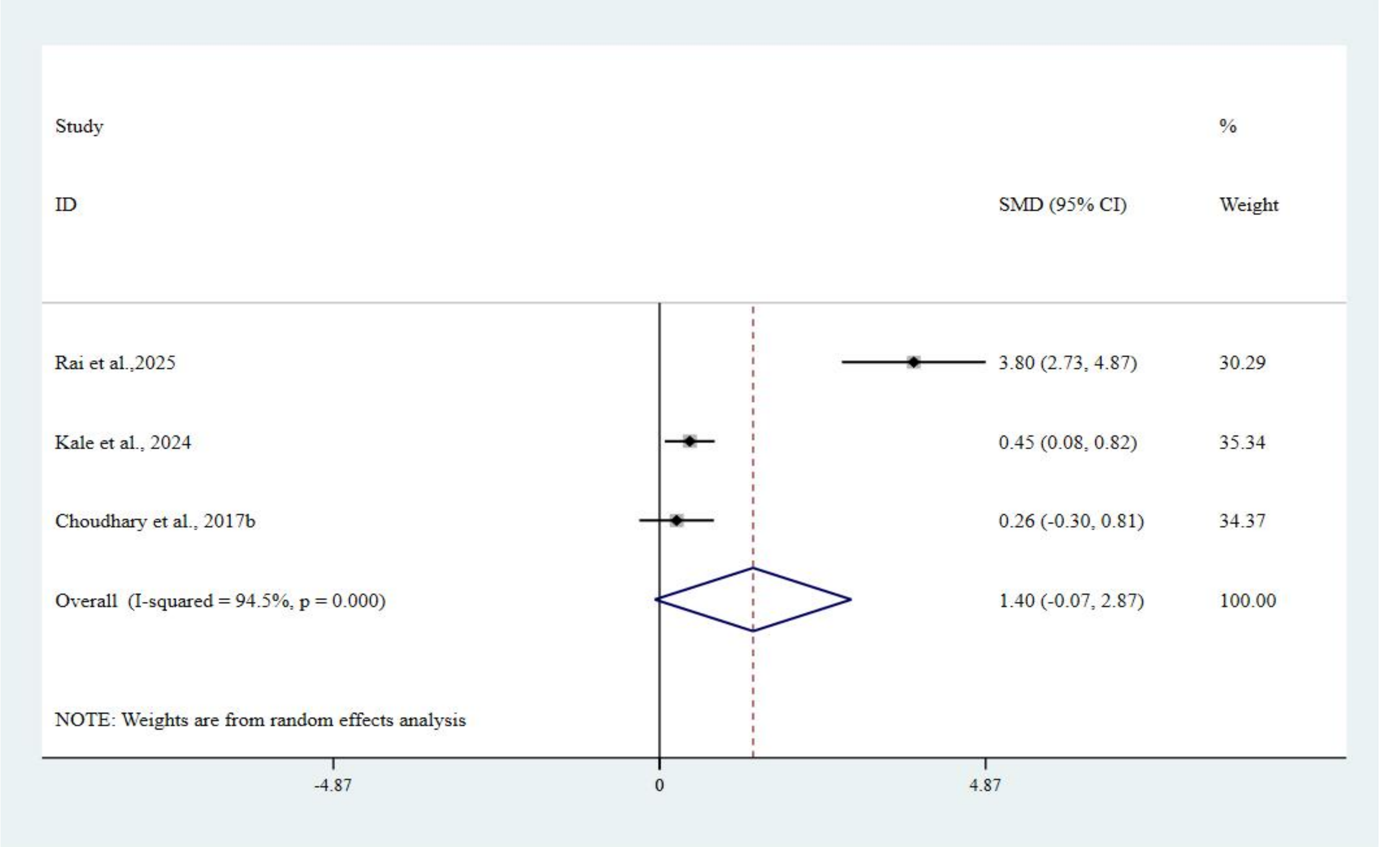

Figure S6. Forest plot of visuospatial ability

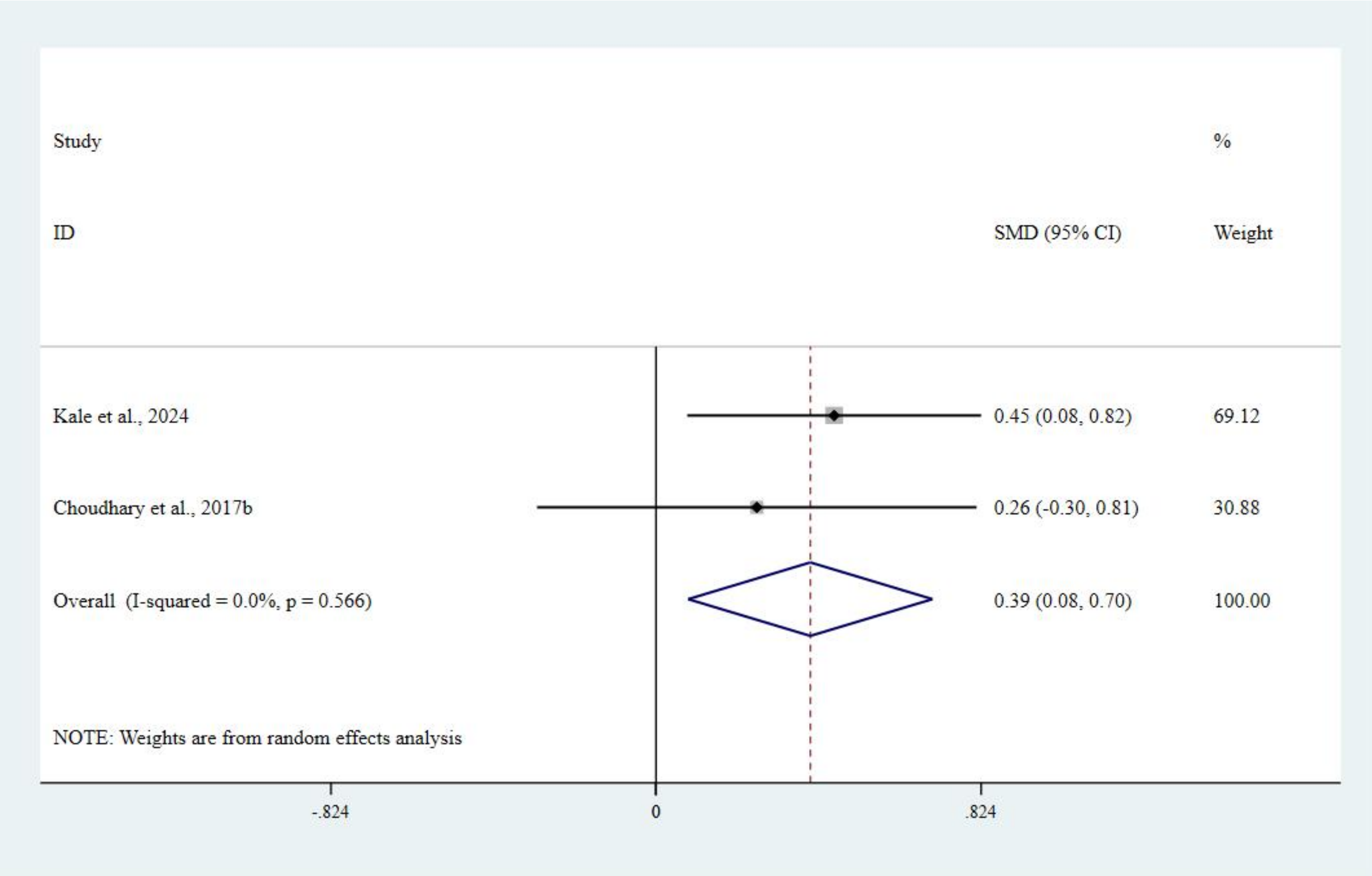

Figure S7. Forest plot of visuospatial ability (excluding Rai et al.,2025)

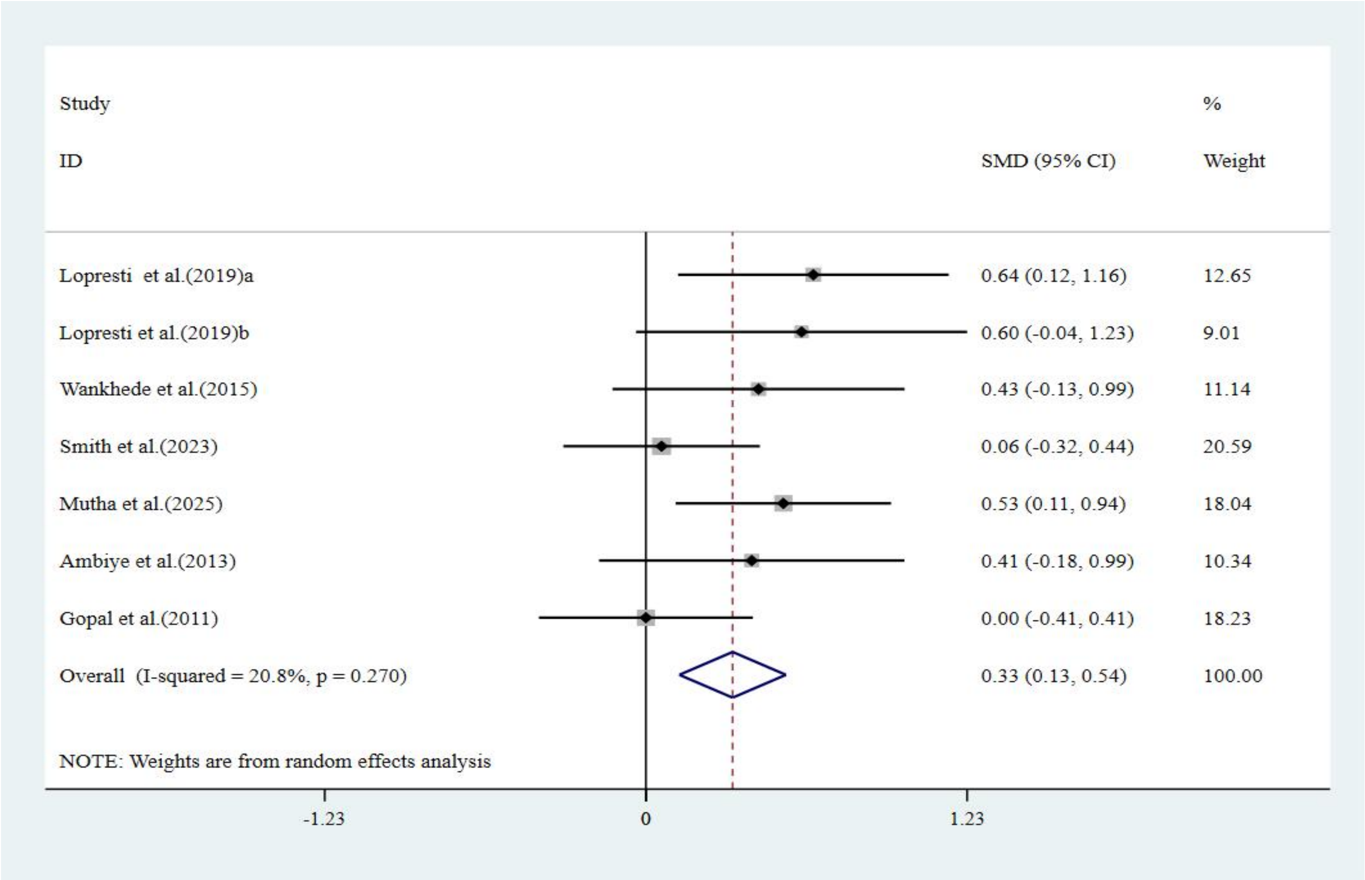

Figure S8. Forest plot of Testosterone levels

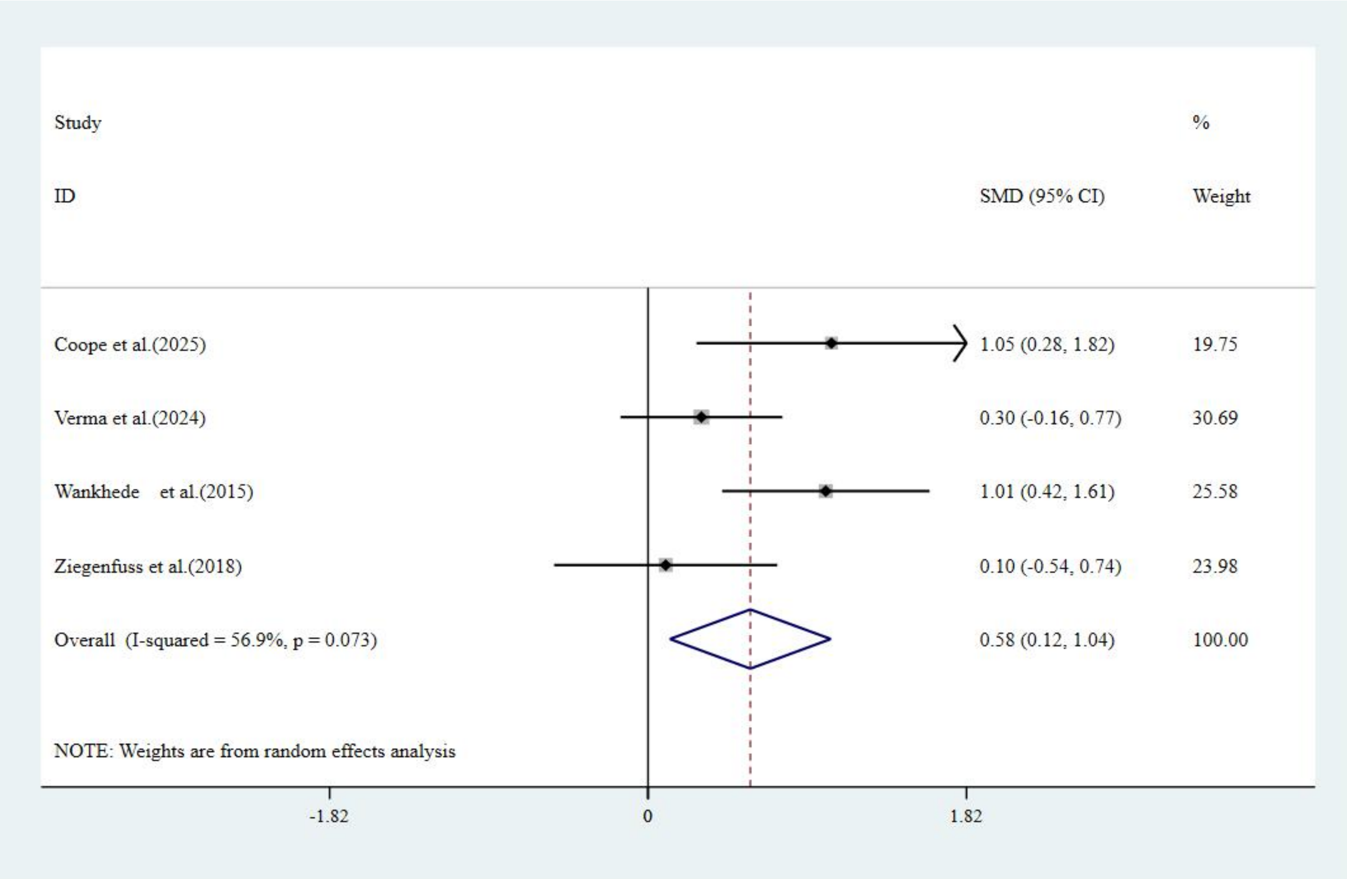

Figure S9. Forest plot of muscle strength

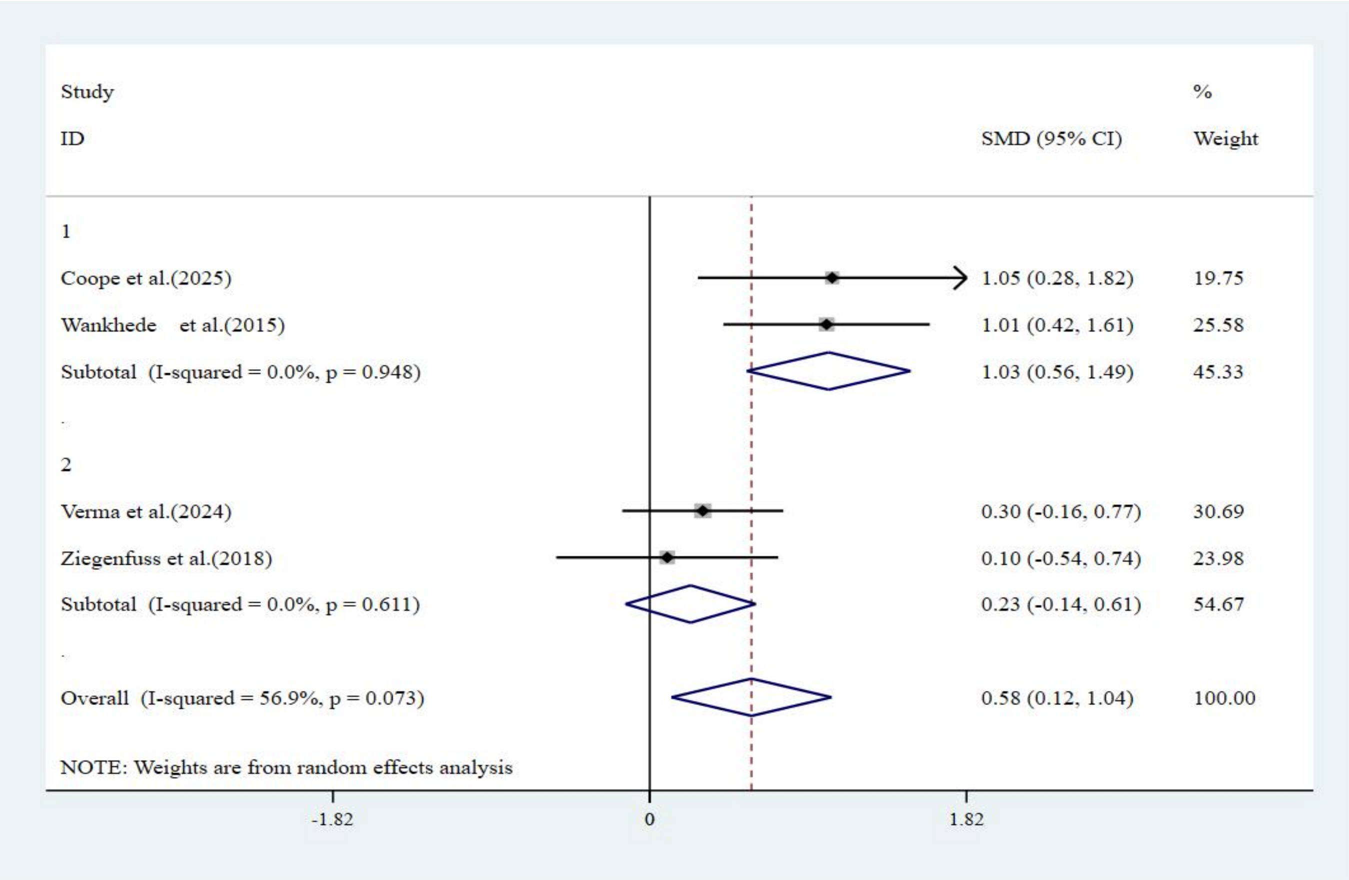

Figure S10. Subgroup analysis for muscle strength by participants' training status revealed

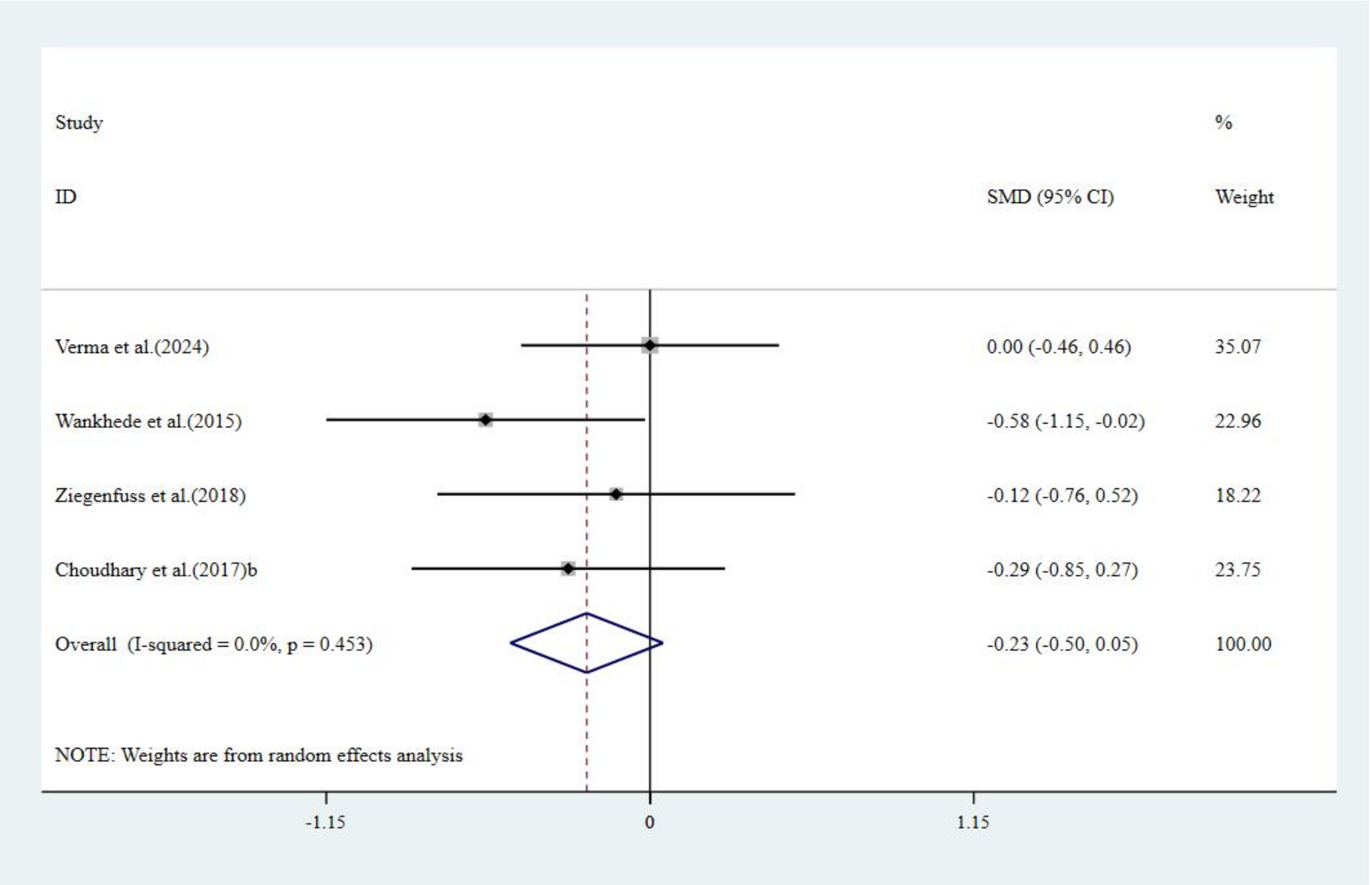

Figure S11. Forest plot of body fat percentage

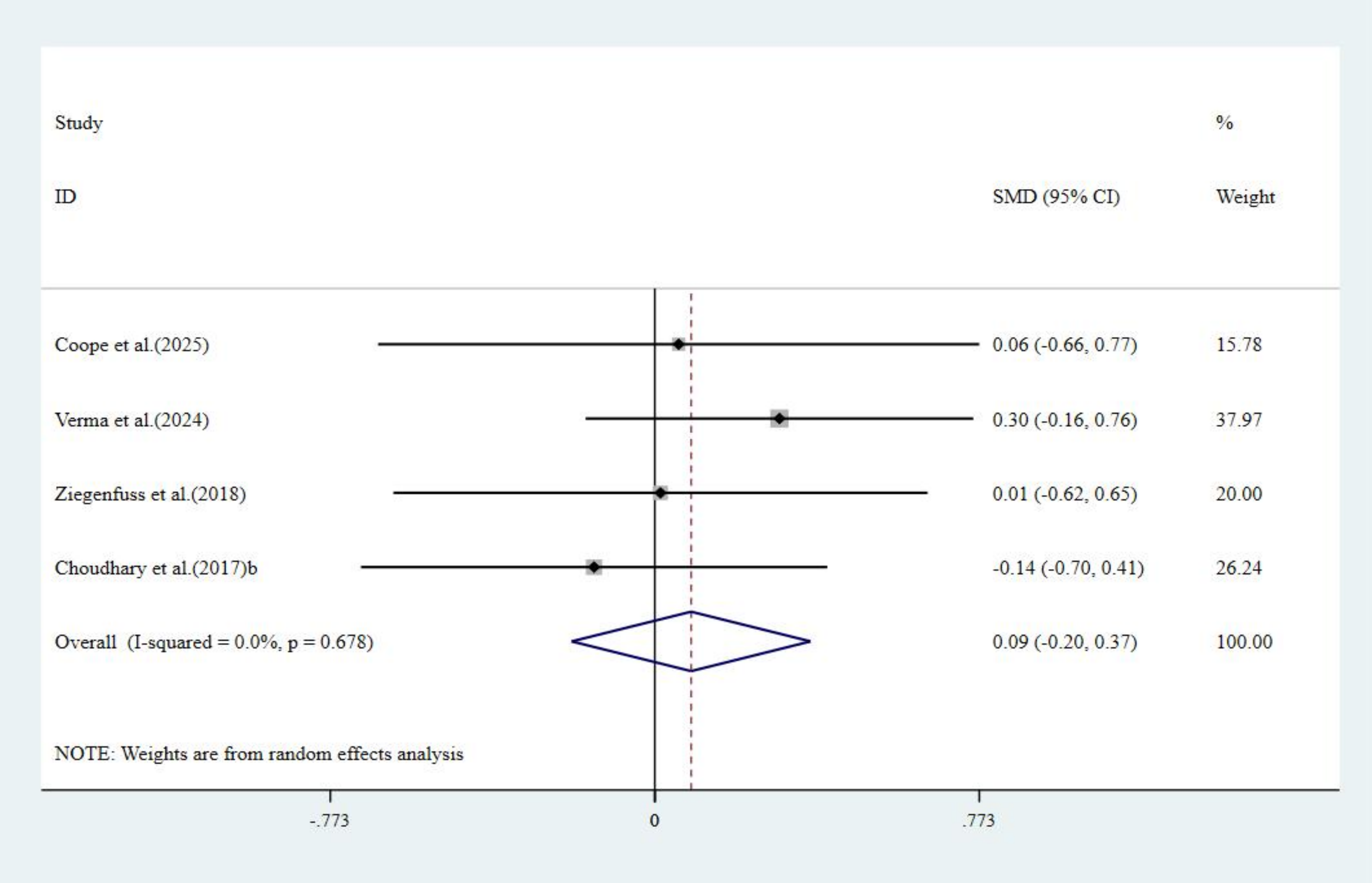

Figure S12. Forest plot of body mass

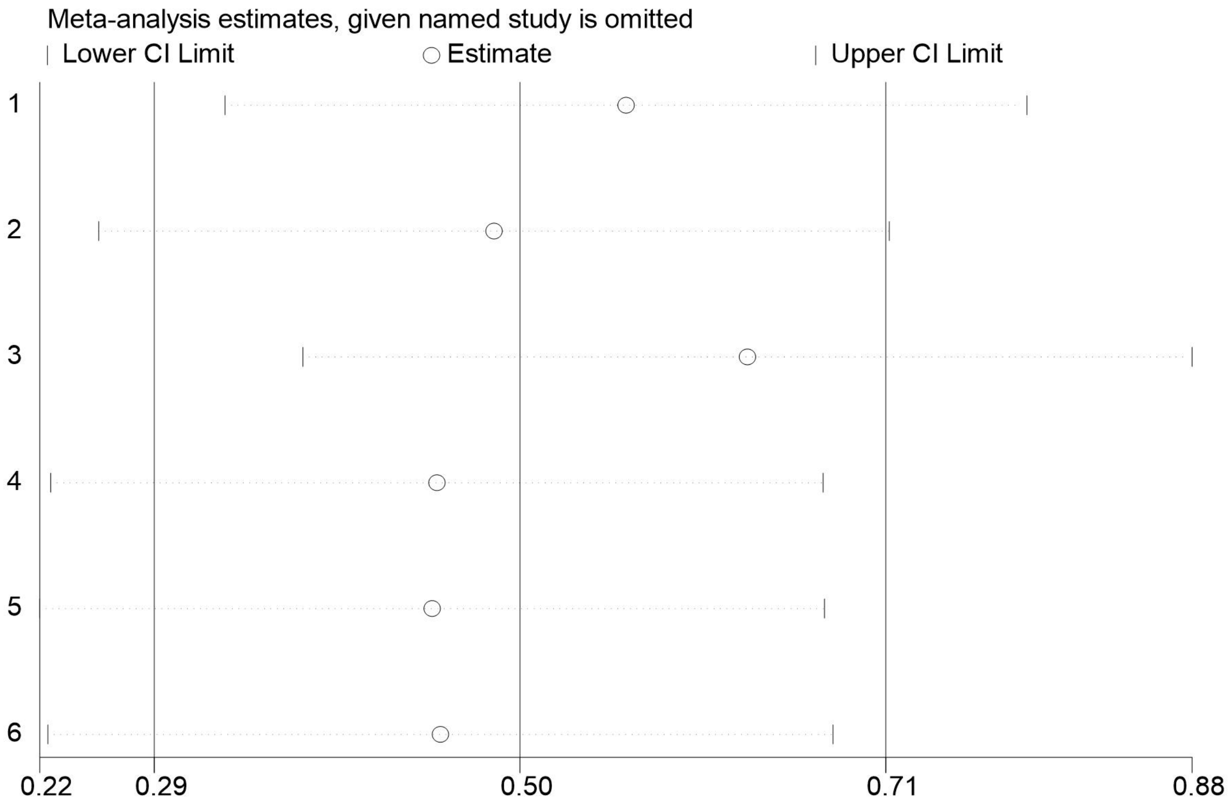

Figure S13. Sensitivity analysis – memory

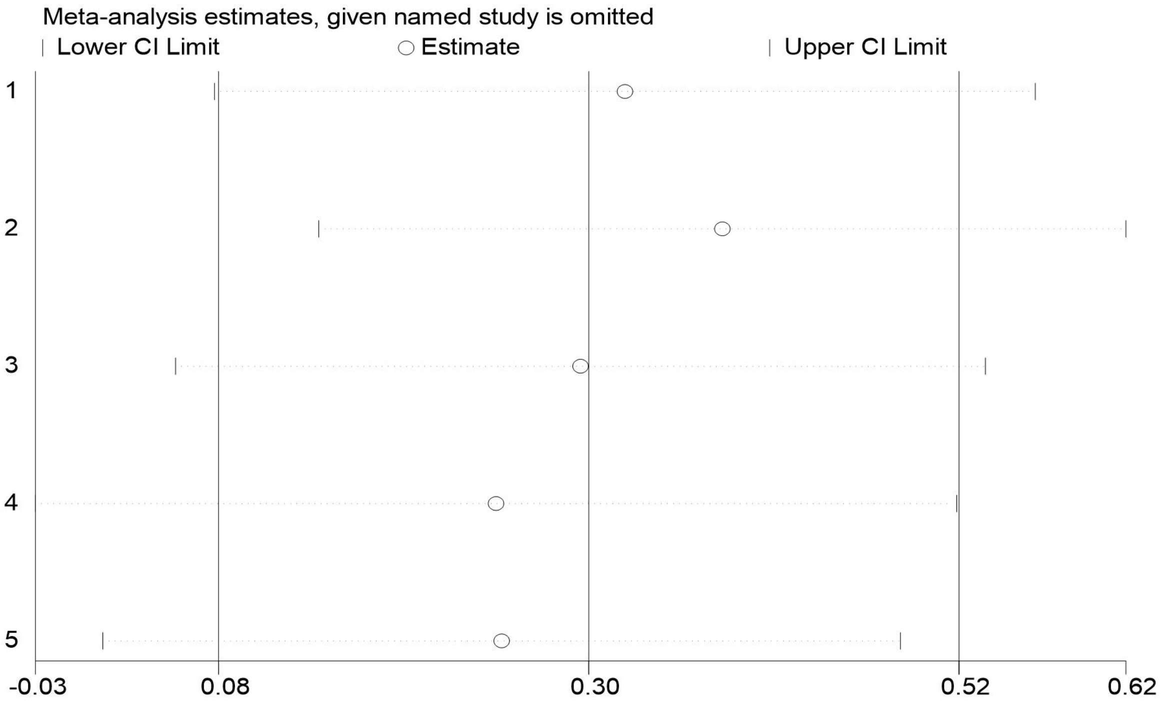

Figure S14. Sensitivity analysis – attention and processing speed

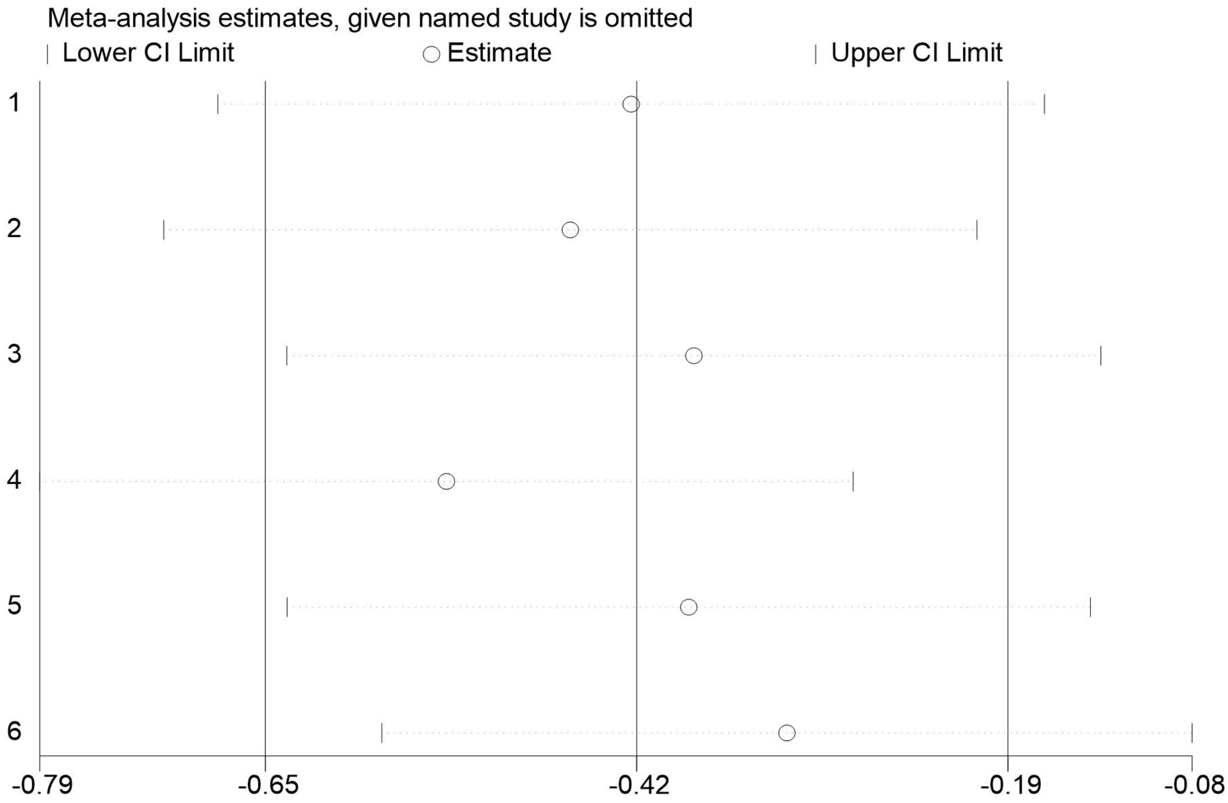

Figure S15. Sensitivity analysis – executive function

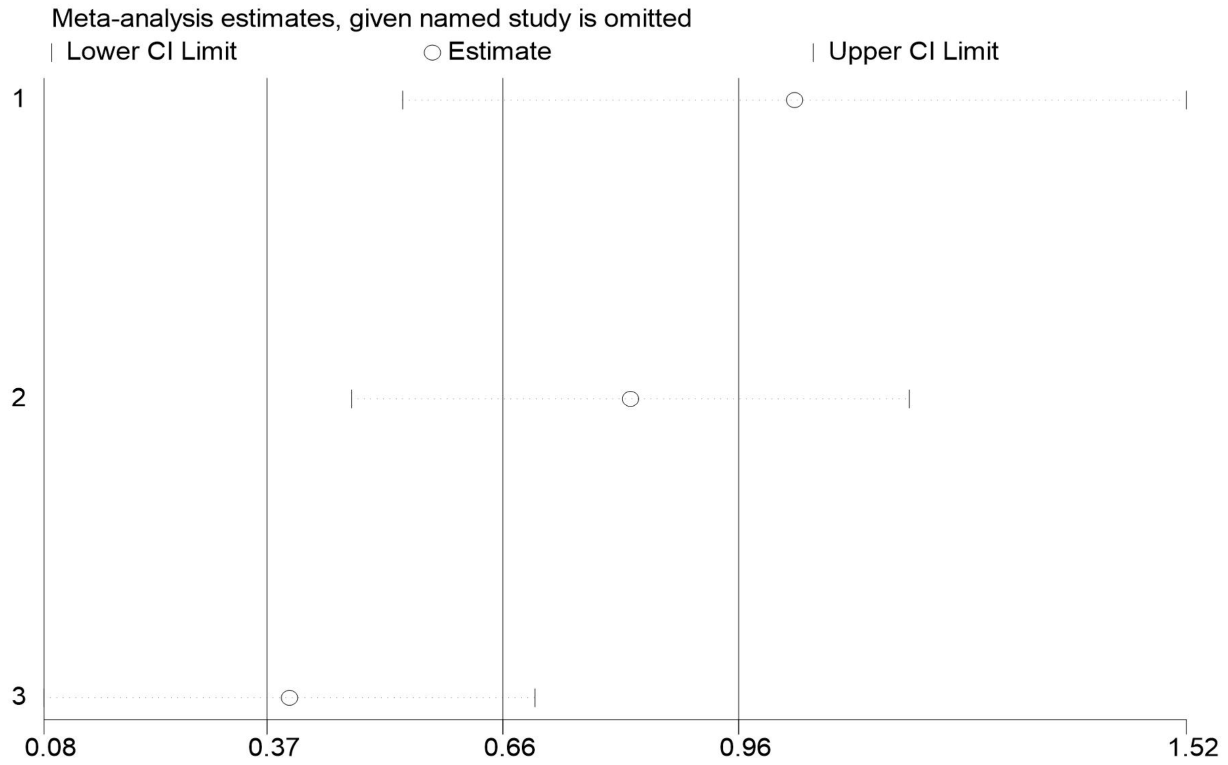

Figure S16. Sensitivity analysis – visuospatial ability

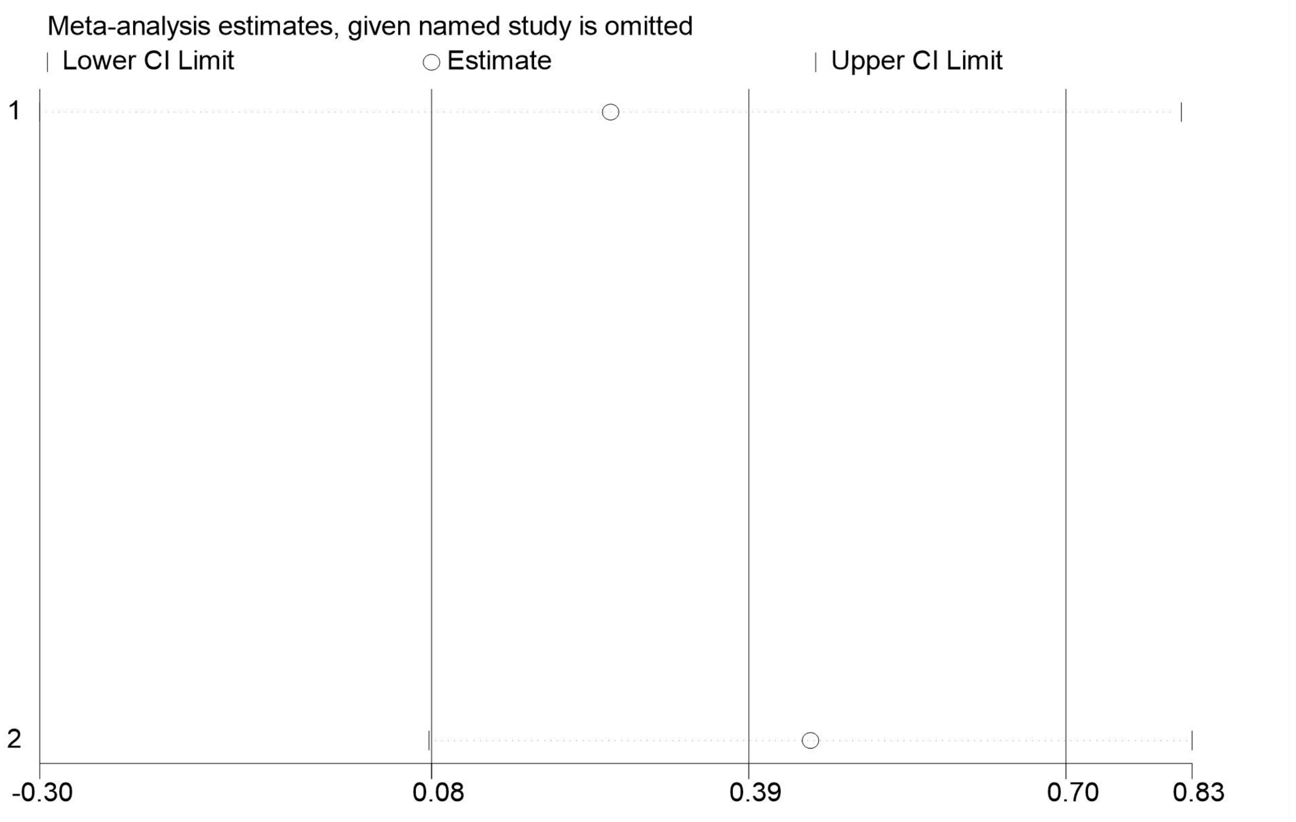

Figure S17. Sensitivity analysis for visuospatial ability (excluding Rai et al.,2025)

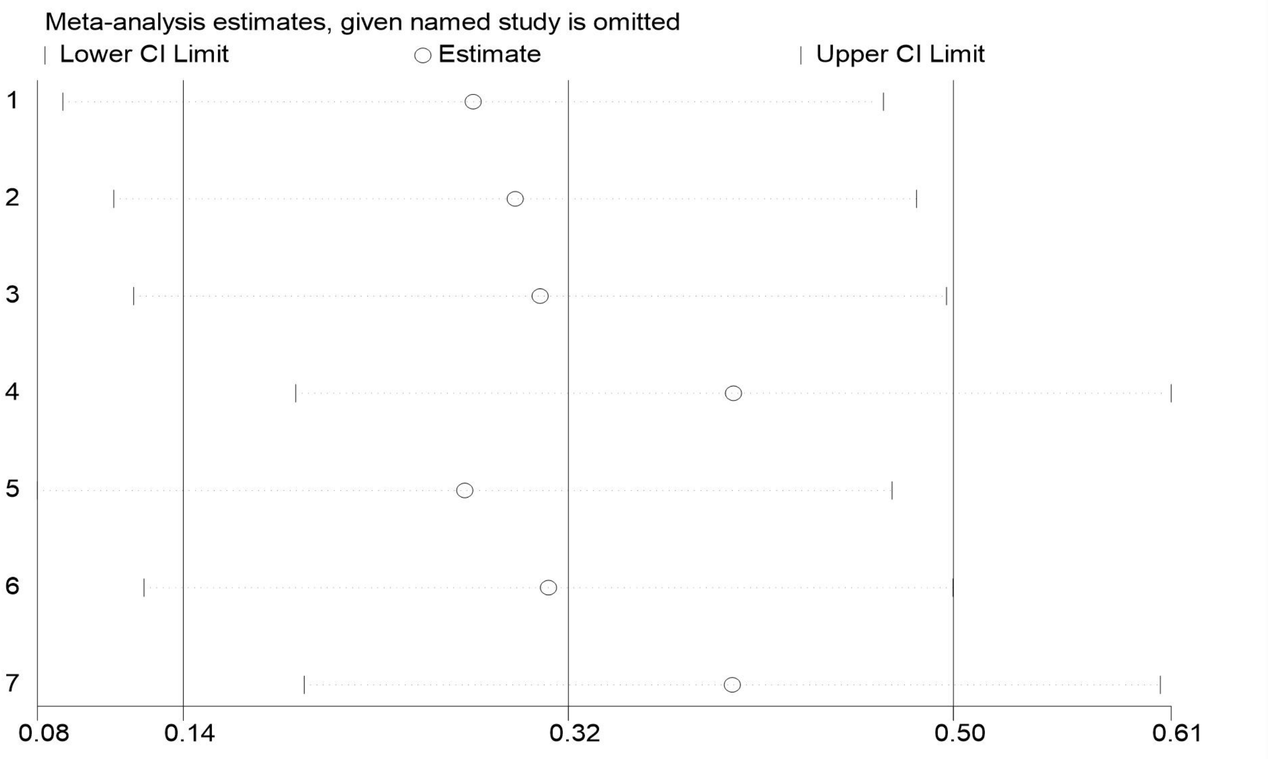

Figure S18. Sensitivity analysis – testosterone levels

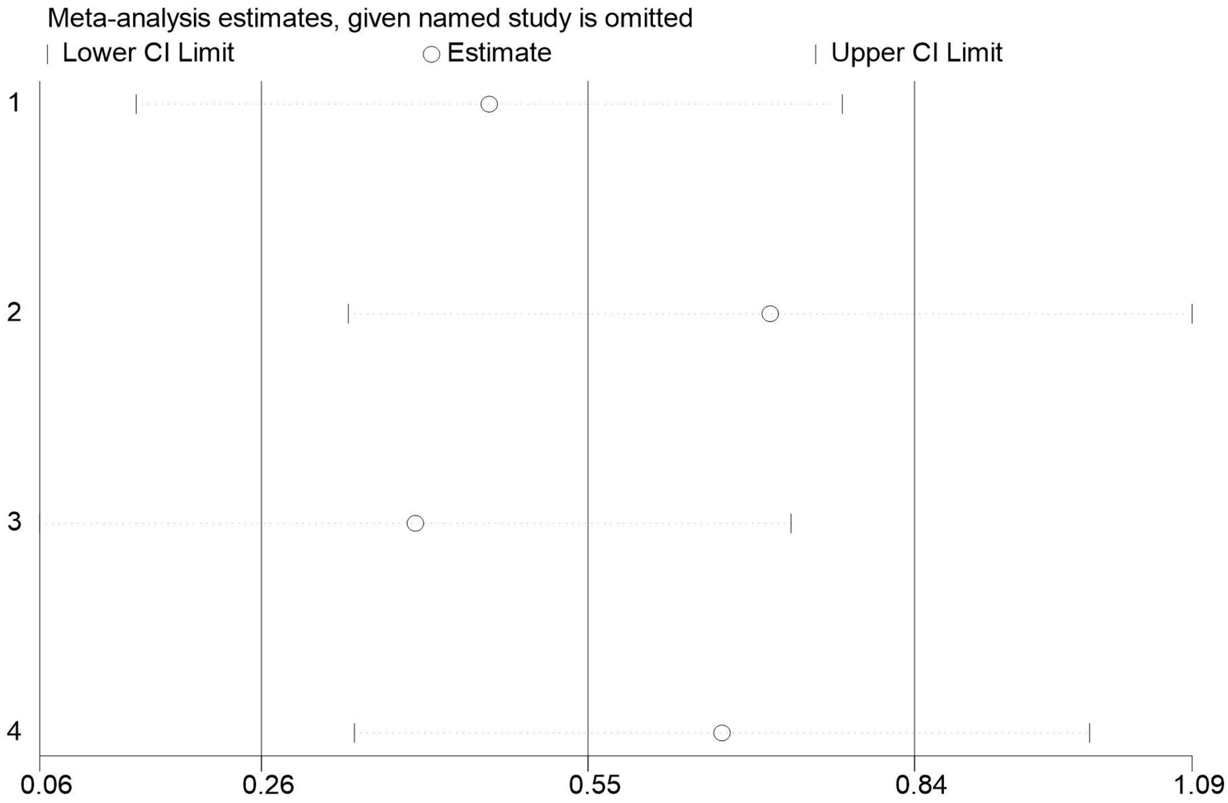

Figure S19. Sensitivity analysis – muscle strength

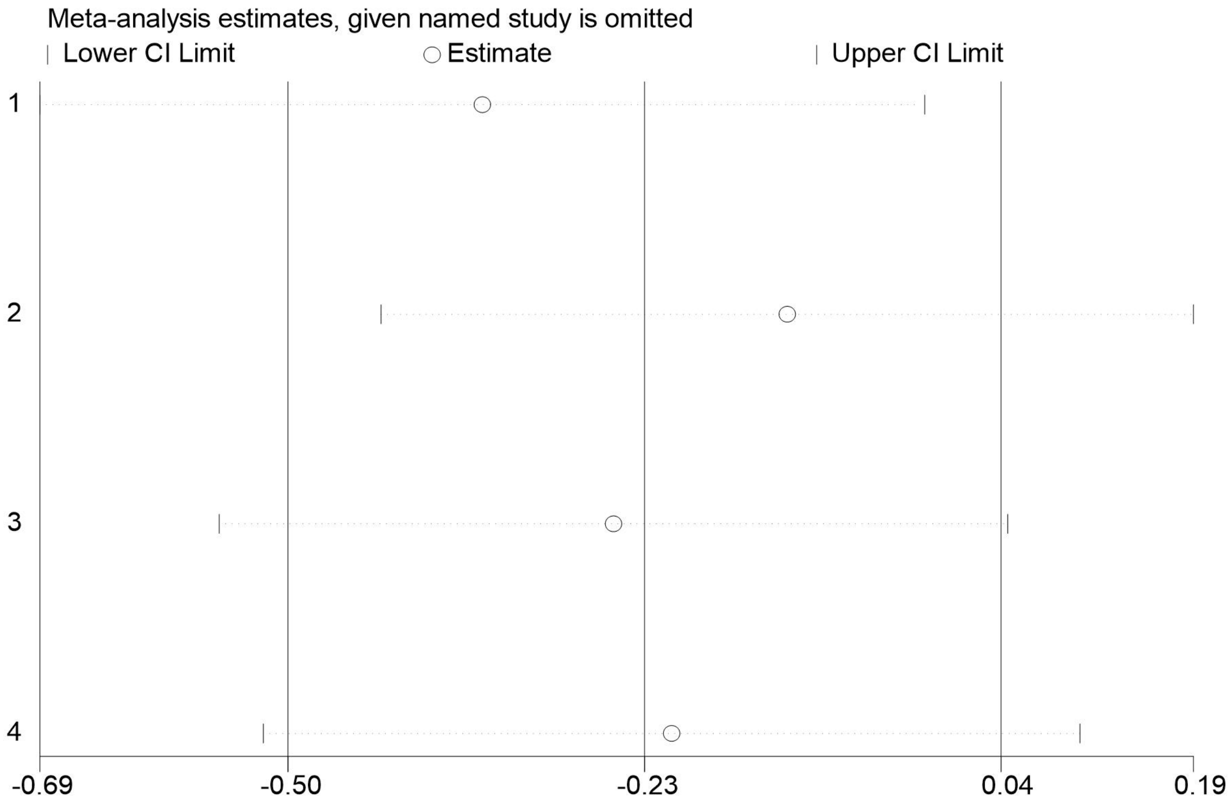

Figure S20. Sensitivity analysis – body fat percentage

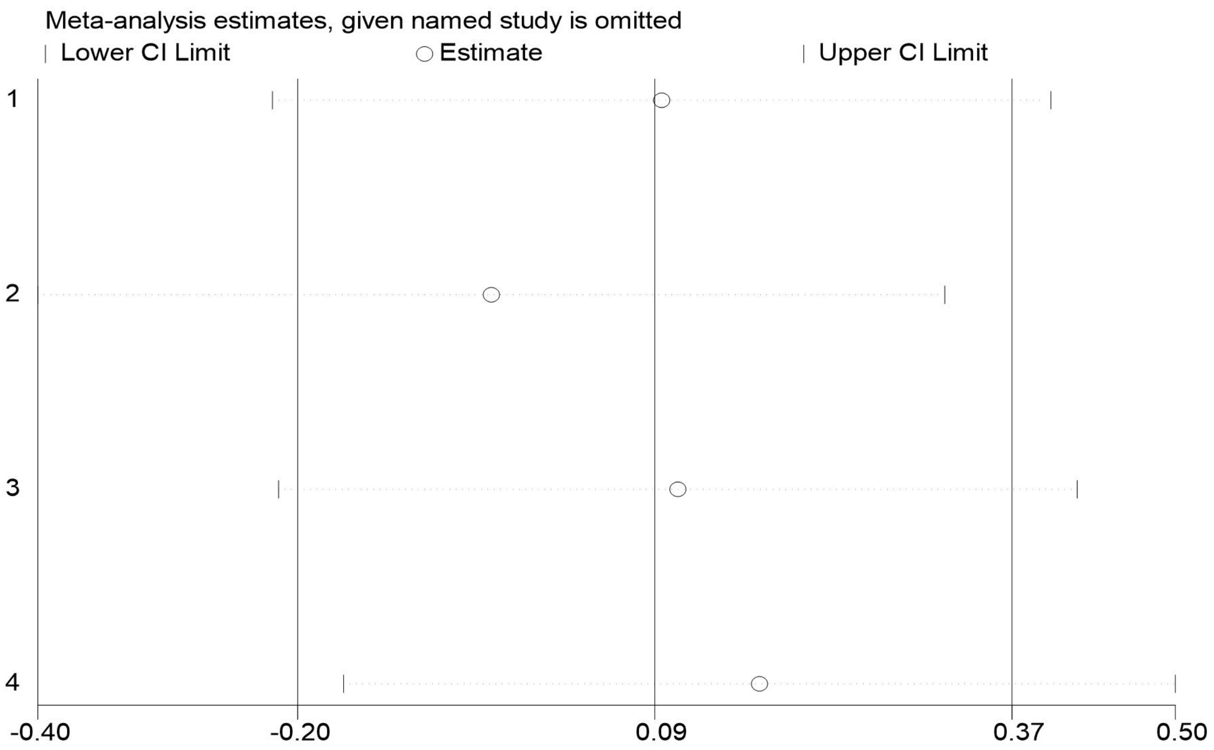

Figure S21. Sensitivity analysis – body mass
