# Supplementary material for: Effects of Withania somnifera (L.) Dunal (Ashwagandha) on cognitive and physical function in adults: a systematic review and meta-analysis
Source: Front Pharmacol. 2026 May 11;17:1799467. doi: 10.3389/fphar.2026.1799467 (PMC13199302; doi:10.3389/fphar.2026.1799467)
Supplement: Supplementary file 2 [file DataSheet1.pdf]

ConPhYMP Tool

1. Rai HP, Mishra DN. Effect of ashwagandha extract with sominone (somin-on™) to improve memory in adults with mild cognitive impairment: A randomized, double-blind, placebo-controlled study. *J Psychopharmacol (Oxf)* (2025) 39:351–363. doi: 10.1177/02698811251324377

2. Coope OC, Reales Salguero A, Spurr T, Páez Calvente A, Domenech Farre A, Jordán Fisas E, Lloyd B, Gooderick J, Abad Sangrà M, Roman-Viñas B. Effects of root extract of ashwagandha on perception of recovery and muscle strength in female athletes. *Eur J Sport Sci* (2025) 25:e12265. doi: 10.1002/ejsc.12265

3. Leonard M, Dickerson B, Estes L, Gonzalez DE, Jenkins V, Johnson S, Xing D, Yoo C, Ko J, Purpura M, et al. Acute and repeated ashwagandha supplementation improves markers of cognitive function and mood. *Nutrients* (2024) 16:1813. doi: 10.3390/nu16121813

4. Verma N, Gupta SK, Patil S, Tiwari S, Mishra AK. Effects of ashwagandha (withania somnifera) standardized root extract on physical endurance and VO2max in healthy adults performing resistance training: An eight- week, prospective, randomized, double-blind, placebo- controlled study. (2024)1–23.

5. Lopresti AL, Smith SJ, Malvi H, Kodgule R. An investigation into the stress-relieving and pharmacological actions of an ashwagandha (withania somnifera) extract: A randomized, double-blind, placebo-controlled study. *Medicine (Baltimore)* (2019) 98:e17186. doi: 10.1097/MD.0000000000017186

6. Lopresti AL, Drummond PD, Smith SJ. A randomized, double-blind, placebo-controlled, crossover study examining the hormonal and vitality effects of ashwagandha in aging, overweight males. *Am J Mens Health* (2019) 13:1557988319835985. doi: 10.1177/1557988319835985

7. Choudhary D, Bhattacharyya S, Joshi K. Body weight management in adults under chronic stress through treatment with ashwagandha root extract: A double-blind, randomized, placebo-controlled trial. *J Evid-Based Complement Altern Med* (2017) 22:96–106. doi: 10.1177/2156587216641830

8. Wankhede S, Langade D, Joshi K, Sinha SR, Bhattacharyya S. Examining the effect of withania somnifera supplementation on muscle strength and recovery: A randomized controlled trial. *J Int Soc Sports Nutr* (2015) 12:43–53. doi: 10.1186/s12970-015-0104-9

9. Chengappa KNR, Bowie CR, Schlicht PJ, Fleet D, Brar JS, Jindal RD. Randomized placebo-controlled adjunctive study of an extract of withania somnifera for cognitive dysfunction in bipolar disorder. (2013)

10. Smith SJ, Lopresti AL, Fairchild TJ. Exploring the efficacy and safety of a novel standardized ashwagandha root extract (witholytin®) in adults experiencing high

stress and fatigue in a randomized, double-blind, placebo-controlled trial. *J Psychopharmacol (Oxf)* (2023) 37:1091–1104. doi: 10.1177/02698811231200023

11. Mutha AS, Mutha SA, Tejuja AH, Beldar AS, Mulay AM, Langade D. Efficacy and safety of eight-week therapy with ashwagandha root extract in improvement of sexual health in healthy men: Findings of a prospective, randomized, double-blind, placebo-controlled study. *J Ayurveda Integr Med* (2025) 16:101155. doi: 10.1016/j.jaim.2025.101155

12. Choudhary D, Bhattacharyya S, Bose S. Efficacy and safety of ashwagandha (withania somnifera (L.) dunal) root extract in improving memory and cognitive functions. *J Diet Suppl* (2017) 14:599–612. doi: 10.1080/19390211.2017.1284970

13. Remenapp A, Coyle K, Orange T, Lynch T, Hooper D, Hooper S, Conway K, Hausenblas HA. Efficacy of withania somnifera supplementation on adult's cognition and mood. *J Ayurveda Integr Med* (2022) 13:100510. doi: 10.1016/j.jaim.2021.08.003

14. Kale S, Lopresti A, Suri R, Garg N, Langade D. Safety and efficacy of ashwagandha root extract on cognition, energy and mood problems in adults: Prospective, randomized, placebo-controlled study. *J Psychoactive Drugs* (2024) 58:45–57. doi: 10.1080/02791072.2024.2424279

15. Xing D, Yoo C, Gonzalez D, Jenkins V, Nottingham K, Dickerson B, Leonard M, Ko J, Faries M, Kephart W, et al. Effects of acute ashwagandha ingestion on cognitive function. *Int J Environ Res Public Health* (2022) 19:11852. doi: 10.3390/ijerph191911852

16. Majeed M, Nagabhushanam K, Mundkur L. A standardized ashwagandha root extract alleviates stress, anxiety, and improves quality of life in healthy adults by modulating stress hormones: Results from a randomized, double-blind, placebo-controlled study. *Medicine (Baltimore)* (2023) 102:e35521. doi: 10.1097/MD.0000000000035521

17. Ziegenfuss TN, Kedia AW, Sandrock JE, Raub BJ, Kerksick CM, Lopez HL. Effects of an aqueous extract of withania somnifera on strength training adaptations and recovery: The STAR trial. *Nutrients* (2018) 10:1807. doi: 10.3390/nu10111807

18. Pingali U, Pilli R, Fatima N. Effect of standardized aqueous extract of withania somniferaon tests of cognitive and psychomotor performance in healthy human participants. *Pharmacogn Res* (2014) 6:12. doi: 10.4103/0974-8490.122912

19. Ambiye VR, Langade D, Dongre S, Aptikar P, Kulkarni M, Dongre A. Clinical evaluation of the spermatogenic activity of the root extract of ashwagandha (withania somnifera) in oligospermic males: A pilot study. *Evid-Based Complement Altern Med ECAM* (2013) 2013:571420. doi: 10.1155/2013/571420

20. Gopal, Ajgaonkar, V T, S C, D L. Effect of an ashwagandha (withania somnifera) root extract on climacteric symptoms in women during perimenopause: a randomized, double-blind, placebo-controlled study. *J Obstet Gynaecol Res* (2021) 47: doi: 10.1111/jog.15030

ConPhyMP checklist of information for reporting plant material and its initial processing [1,2] (relevant for all studies on medicinal and food plants including extract types A, B, and C)

SECTION/TOPIC	ITEM NO.	CHECKLIST ITEM	YES	NO	NOT APPLICABLE	PAGE NO., IF ANY
Title and abstract	1	A clear and concise title including an informative abstract and balanced summary.	✓			
Description of the botanical drug and taxonomic authentication	2	Botanical or morphological authentication of the plant material (desirable is a combination with DNA barcoding, e.g., PCR, RFLP, genome sequencing) and the information must be included in a separate section of Material and Methods, if applicable, combined with the information required under item 3:	✓			
Description of the extract and extraction process	3	A separate section in Material and Methods, covers the relevant information on the material investigated, including the full species name(s), authorites and family; e.g. *Salvia miltorrhiza* Bunge [Lamiaceae; *Salviae miltorrhizae radix et rhizoma*], and on the processing and extraction of the crude drug including the traditional processing of the material used medicinally (fumigation, steaming, roasting, cooking, frying, etc.).		✓		
Documentation of the legal basis for collection and processing	4	Full compliance with the Nagoya protocol, CITES, and all associated treaties including phytosanitary regulations.		✓		
Description of product characteristics, in case of a finished (commercial) product	5	Information on the characteristics of the commercial products including batch number and date of production/best by information and regulatory status.	✓			

Note: Please also include here the following information about your submitted manuscript:

Name of the journal:	Journal Of Psychopharmacology
Date of the enquiry:	2025 Apr
Title of the manuscript:	A randomized, double-blind, placebo-controlled study
List of the authors:	Hari Prakash Rai , Deo Nidhi Mishra

[1] Please acknowledge/cite this as follows: Heinrich M, Jalil B, Abdel-Tawab M, Echeverria J, Kulić Ž, McGaw LJ, et al. Best Practice in the chemical characterisation of extracts used in pharmacological and toxicological research—The ConPhyMP—Guidelines. Frontiers in Pharmacology. 2022;13:953205. https://doi.org/10.3389/fphar.2022.953205

[2] We strongly recommend reading this checklist in conjuncton with ConPhyMP 2022 explanaton and elaboraton for important clarifications on all items. If relevant, we also recommend after reading Heinrich et al. (2020) Best practice in research—Overcoming common challenges in phytopharmacological research. Journal of Ethnopharmacology. 2020;246:112230. https://doi.org/10.1016/j.jep.2019.112230

ConPhyMP checklist of items for conducting and reporting analytical methods[1,2] relevant for extract type A (for species or botanical drugs covered in a monograph in one of the national or regional pharmacopoeias)

SECTION/TOPIC	ITEM NO.	CHECKLIST ITEM	YES	NO	NOT APPLICABLE	PAGE NO., IF ANY
Type of extract	1	A – Confirm that the species or botanical drug under investigation is covered in a monograph in one of the national or regional pharmacopoeias.	✓			
Preferred/main methods for extract characterisation/ chemical analysis	2	Compliance with pharmacopoeial standards to be followed:				
		(a) The description of the active ingredients in the botanical drug (if known) or analytical marker compounds as defined.	✓			
		(b) An analysis as defined in the monograph is needed if the extract has not been supplied with a certificate.		✓		
		(c) If the preparation was purchased, the manufacturer and certificate of analysis need to be included.	✓			
		Including either the preferred or alternative approaches for characterisation:				
		(a) Triple chemical fingerprinting methods, each with one or more detection parameters.		✓		
		(b) Quantification of at least two marker compounds (unless this is not feasible, evidence needs to be provided), and justification of the choice of markers (if applicable).	✓			
Alternative methods for extract characterisation/chemical analysis	3	(a) Single chemical fingerprinting method with at least three different detection parameters (i.e., altered detection parameters, like TLC/HPTLC with different staining reagents and/or UV excitation wave-lengths, HPLC-DAD/LCDAD with different wavelengths). The same applies to coupling MS or NMR to chromatographic techniques.		✓		
		(b) Quantification of at least two marker compounds (unless this is not feasible, evidence needs to be provided), and justification of the choice of markers (if applicable).	✓			
Use of reference standards	4	(a) Direct overlay of the chromatogram of the sample with that of an officially specified reference standard (if applicable).	✓			
		(b) Chromatographic fingerprinting: Direct overlay of the chromato-gram of the sample with that of official reference standards of the powdered plant material or the dry extract from the plant material.				
Comparison of different extracts/ samples of the same plants	5	(a) Direct comparison of the chromatographic/spectroscopic system and/or scoring system for "similarity" to be followed.			✓	

Note: Please also include here the following information about your submitted manuscript:

Name of the journal:	Journal Of Psychopharmacology
Date of the enquiry:	2025 Apr
Title of the manuscript:	A randomized, double-blind, placebo-controlled study
List of the authors:	Hari Prakash Rai , Deo Nidhi Mishra

[1] Please acknowledge/cite this as follows: Heinrich M, Jalil B, Abdel-Tawab M, Echeverria J, Kulić Ž, McGaw LJ, et al. Best Practice in the chemical characterisation of extracts used in pharmacological and toxicological research—The ConPhyMP—Guidelines. Frontiers in Pharmacology. 2022;13:953205. https://doi.org/10.3389/fphar.2022.953205

[2] We strongly recommend reading this checklist in conjuncton with ConPhyMP 2022 explanaton and elaboraton for important clarifications on all items. If relevant, we also recommend after reading Heinrich et al. (2020) Best practice in research—Overcoming common challenges in phytopharmacological research. Journal of Ethnopharmacology. 2020;246:112230. https://doi.org/10.1016/j.jep.2019.112230

ConPhyMP checklist of information for reporting plant material and its initial processing [1,2] (relevant for all studies on medicinal and food plants including extract types A, B, and C)

SECTION/TOPIC	ITEM NO.	CHECKLIST ITEM	YES	NO	NOT APPLICABLE	PAGE NO., IF ANY
Title and abstract	1	A clear and concise title including an informative abstract and balanced summary.	✓			
Description of the botanical drug and taxonomic authentication	2	Botanical or morphological authentication of the plant material (desirable is a combination with DNA barcoding, e.g., PCR, RFLP, genome sequencing) and the information must be included in a separate section of Material and Methods, if applicable, combined with the information required under item 3:	✓			
Description of the extract and extraction process	3	A separate section in Material and Methods, covers the relevant information on the material investigated, including the full species name(s), authorites and family; e.g. *Salvia miltorrhiza* Bunge [Lamiaceae; *Salviae miltorrhizae radix et rhizoma*], and on the processing and extraction of the crude drug including the traditional processing of the material used medicinally (fumigation, steaming, roasting, cooking, frying, etc.).		✓		
Documentation of the legal basis for collection and processing	4	Full compliance with the Nagoya protocol, CITES, and all associated treaties including phytosanitary regulations.			✓	
Description of product characteristics, in case of a finished (commercial) product	5	Information on the characteristics of the commercial products including batch number and date of production/best by information and regulatory status.	✓			

Note: Please also include here the following information about your submitted manuscript:

Name of the journal:	European Journal of Sport Science
Date of the enquiry:	2025 Mar
Title of the manuscript:	on of recovery and muscle strength in female athletes
List of the authors:	ooderick 6, Maria Abad Sangrà 7, Blanca Roman-Viñas 8

[1] Please acknowledge/cite this as follows: Heinrich M, Jalil B, Abdel-Tawab M, Echeverria J, Kulić Ž, McGaw LJ, et al. Best Practice in the chemical characterisation of extracts used in pharmacological and toxicological research—The ConPhyMP—Guidelines. Frontiers in Pharmacology. 2022;13:953205. https://doi.org/10.3389/fphar.2022.953205

[2] We strongly recommend reading this checklist in conjuncton with ConPhyMP 2022 explanaton and elaboraton for important clarifications on all items. If relevant, we also recommend after reading Heinrich et al. (2020) Best practice in research—Overcoming common challenges in phytopharmacological research. Journal of Ethnopharmacology. 2020;246:112230. https://doi.org/10.1016/j.jep.2019.112230

ConPhyMP checklist of items for conducting and reporting analytical methods[1,2] relevant for extract type A (for species or botanical drugs covered in a monograph in one of the national or regional pharmacopoeias)

SECTION/TOPIC	ITEM NO.	CHECKLIST ITEM	YES	NO	NOT APPLICABLE	PAGE NO., IF ANY
Type of extract	1	A – Confirm that the species or botanical drug under investigation is covered in a monograph in one of the national or regional pharmacopoeias.	✓			
Preferred/main methods for extract characterisation/ chemical analysis	2	Compliance with pharmacopoeial standards to be followed:				
		(a) The description of the active ingredients in the botanical drug (if known) or analytical marker compounds as defined.		✓		
		(b) An analysis as defined in the monograph is needed if the extract has not been supplied with a certificate.		✓		
		(c) If the preparation was purchased, the manufacturer and certificate of analysis need to be included.	✓			
		Including either the preferred or alternative approaches for characterisation:				
		(a) Triple chemical fingerprinting methods, each with one or more detection parameters.		✓		
		(b) Quantification of at least two marker compounds (unless this is not feasible, evidence needs to be provided), and justification of the choice of markers (if applicable).		✓		
Alternative methods for extract characterisation/chemical analysis	3	(a) Single chemical fingerprinting method with at least three different detection parameters (i.e., altered detection parameters, like TLC/ HPTLC with different staining reagents and/or UV excitation wave-lengths, HPLC-DAD/LCDAD with different wavelengths). The same applies to coupling MS or NMR to chromatographic techniques.		✓		
		(b) Quantification of at least two marker compounds (unless this is not feasible, evidence needs to be provided), and justification of the choice of markers (if applicable).		✓		
Use of reference standards	4	(a) Direct overlay of the chromatogram of the sample with that of an officially specified reference standard (if applicable).		✓		
		(b) Chromatographic fingerprinting: Direct overlay of the chromato-gram of the sample with that of official reference standards of the powdered plant material or the dry extract from the plant material.				
Comparison of different extracts/ samples of the same plants	5	(a) Direct comparison of the chromatographic/spectroscopic system and/or scoring system for "similarity" to be followed.			✓	

Note: Please also include here the following information about your submitted manuscript:

Name of the journal:	European Journal of Sport Science
Date of the enquiry:	2025 Mar
Title of the manuscript:	on of recovery and muscle strength in female athletes
List of the authors:	ooderick 6, Maria Abad Sangrà 7, Blanca Roman-Viñas 8

[1] Please acknowledge/cite this as follows: Heinrich M, Jalil B, Abdel-Tawab M, Echeverria J, Kulić Ž, McGaw LJ, et al. Best Practice in the chemical characterisation of extracts used in pharmacological and toxicological research—The ConPhyMP—Guidelines. Frontiers in Pharmacology. 2022;13:953205. https://doi.org/10.3389/fphar.2022.953205

[2] We strongly recommend reading this checklist in conjuncton with ConPhyMP 2022 explanaton and elaboraton for important clarifications on all items. If relevant, we also recommend after reading Heinrich et al. (2020) Best practice in research—Overcoming common challenges in phytopharmacological research. Journal of Ethnopharmacology. 2020;246:112230. https://doi.org/10.1016/j.jep.2019.112230

ConPhyMP checklist of information for reporting plant material and its initial processing [1,2] (relevant for all studies on medicinal and food plants including extract types A, B, and C)

SECTION/TOPIC	ITEM NO.	CHECKLIST ITEM	YES	NO	NOT APPLICABLE	PAGE NO., IF ANY
Title and abstract	1	A clear and concise title including an informative abstract and balanced summary.	✓			
Description of the botanical drug and taxonomic authentication	2	Botanical or morphological authentication of the plant material (desirable is a combination with DNA barcoding, e.g., PCR, RFLP, genome sequencing) and the information must be included in a separate section of Material and Methods, if applicable, combined with the information required under item 3:	✓			
Description of the extract and extraction process	3	A separate section in Material and Methods, covers the relevant information on the material investigated, including the full species name(s), authorites and family; e.g. *Salvia miltorrhiza* Bunge [Lamiaceae; *Salviae miltorrhizae radix et rhizoma*], and on the processing and extraction of the crude drug including the traditional processing of the material used medicinally (fumigation, steaming, roasting, cooking, frying, etc.).	✓			
Documentation of the legal basis for collection and processing	4	Full compliance with the Nagoya protocol, CITES, and all associated treaties including phytosanitary regulations.			✓	
Description of product characteristics, in case of a finished (commercial) product	5	Information on the characteristics of the commercial products including batch number and date of production/best by information and regulatory status.	✓			

Note: Please also include here the following information about your submitted manuscript:

Name of the journal: Nutrients

Date of the enquiry: 2024 Jun 8

Title of the manuscript: ation Improves Markers of Cognitive Function and Mood

List of the authors: ski 1, Christopher J Rasmussen 1, Richard B Kreider 1

[1] Please acknowledge/cite this as follows: Heinrich M, Jalil B, Abdel-Tawab M, Echeverria J, Kulić Ž, McGaw LJ, et al. Best Practice in the chemical characterisation of extracts used in pharmacological and toxicological research—The ConPhyMP—Guidelines. Frontiers in Pharmacology. 2022;13:953205. https://doi.org/10.3389/fphar.2022.953205

[2] We strongly recommend reading this checklist in conjuncton with ConPhyMP 2022 explanaton and elaboraton for important clarifications on all items. If relevant, we also recommend after reading Heinrich et al. (2020) Best practice in research—Overcoming common challenges in phytopharmacological research. Journal of Ethnopharmacology. 2020;246:112230. https://doi.org/10.1016/j.jep.2019.112230

ConPhyMP checklist of items for conducting and reporting analytical methods[1,2] relevant for extract type A (for species or botanical drugs covered in a monograph in one of the national or regional pharmacopoeias)

SECTION/TOPIC	ITEM NO.	CHECKLIST ITEM	YES	NO	NOT APPLICABLE	PAGE NO., IF ANY
Type of extract	1	A – Confirm that the species or botanical drug under investigation is covered in a monograph in one of the national or regional pharmacopoeias.	✓			
Preferred/main methods for extract characterisation/ chemical analysis	2	Compliance with pharmacopoeial standards to be followed:				
		(a) The description of the active ingredients in the botanical drug (if known) or analytical marker compounds as defined.		✓		
		(b) An analysis as defined in the monograph is needed if the extract has not been supplied with a certificate.	✓			
		(c) If the preparation was purchased, the manufacturer and certificate of analysis need to be included.	✓			
		Including either the preferred or alternative approaches for characterisation:				
		(a) Triple chemical fingerprinting methods, each with one or more detection parameters.		✓		
		(b) Quantification of at least two marker compounds (unless this is not feasible, evidence needs to be provided), and justification of the choice of markers (if applicable).		✓		
Alternative methods for extract characterisation/chemical analysis	3	(a) Single chemical fingerprinting method with at least three different detection parameters (i.e., altered detection parameters, like TLC/ HPTLC with different staining reagents and/or UV excitation wave-lengths, HPLC-DAD/LCDAD with different wavelengths). The same applies to coupling MS or NMR to chromatographic techniques.		✓		
		(b) Quantification of at least two marker compounds (unless this is not feasible, evidence needs to be provided), and justification of the choice of markers (if applicable).		✓		
Use of reference standards	4	(a) Direct overlay of the chromatogram of the sample with that of an officially specified reference standard (if applicable).		✓		
		(b) Chromatographic fingerprinting: Direct overlay of the chromato-gram of the sample with that of official reference standards of the powdered plant material or the dry extract from the plant material.				
Comparison of different extracts/ samples of the same plants	5	(a) Direct comparison of the chromatographic/spectroscopic system and/or scoring system for "similarity" to be followed.			✓	

Note: Please also include here the following information about your submitted manuscript:

Name of the journal: Nutrients

Date of the enquiry: 2024 Jun 8

Title of the manuscript: ation Improves Markers of Cognitive Function and Mood

List of the authors: ski 1, Christopher J Rasmussen 1, Richard B Kreider 1

[1] Please acknowledge/cite this as follows: Heinrich M, Jalil B, Abdel-Tawab M, Echeverria J, Kulić Ž, McGaw LJ, et al. Best Practice in the chemical characterisation of extracts used in pharmacological and toxicological research—The ConPhyMP—Guidelines. Frontiers in Pharmacology. 2022;13:953205. https://doi.org/10.3389/fphar.2022.953205

[2] We strongly recommend reading this checklist in conjuncton with ConPhyMP 2022 explanaton and elaboraton for important clarifications on all items. If relevant, we also recommend after reading Heinrich et al. (2020) Best practice in research—Overcoming common challenges in phytopharmacological research. Journal of Ethnopharmacology. 2020;246:112230. https://doi.org/10.1016/j.jep.2019.112230

ConPhyMP checklist of information for reporting plant material and its initial processing [1,2] (relevant for all studies on medicinal and food plants including extract types A, B, and C)

SECTION/TOPIC	ITEM NO.	CHECKLIST ITEM	YES	NO	NOT APPLICABLE	PAGE NO., IF ANY
Title and abstract	1	A clear and concise title including an informative abstract and balanced summary.	✓			
Description of the botanical drug and taxonomic authentication	2	Botanical or morphological authentication of the plant material (desirable is a combination with DNA barcoding, e.g., PCR, RFLP, genome sequencing) and the information must be included in a separate section of Material and Methods, if applicable, combined with the information required under item 3:	✓			
Description of the extract and extraction process	3	A separate section in Material and Methods, covers the relevant information on the material investigated, including the full species name(s), authorites and family; e.g. *Salvia miltorrhiza* Bunge [Lamiaceae; *Salviae miltorrhizae radix et rhizoma*], and on the processing and extraction of the crude drug including the traditional processing of the material used medicinally (fumigation, steaming, roasting, cooking, frying, etc.).	✓			
Documentation of the legal basis for collection and processing	4	Full compliance with the Nagoya protocol, CITES, and all associated treaties including phytosanitary regulations.			✓	
Description of product characteristics, in case of a finished (commercial) product	5	Information on the characteristics of the commercial products including batch number and date of production/best by information and regulatory status.	✓			

Note: Please also include here the following information about your submitted manuscript:

Name of the journal: F1000Research

Date of the enquiry: 2024 Apr

Title of the manuscript: e, randomized, double-blind, placebo-controlled study

List of the authors: yali Patil 3, Shashank Tiwari 1, Ashok Kumar Mishra 4

[1] Please acknowledge/cite this as follows: Heinrich M, Jalil B, Abdel-Tawab M, Echeverria J, Kulić Ž, McGaw LJ, et al. Best Practice in the chemical characterisation of extracts used in pharmacological and toxicological research—The ConPhyMP—Guidelines. Frontiers in Pharmacology. 2022;13:953205. https://doi.org/10.3389/fphar.2022.953205

[2] We strongly recommend reading this checklist in conjuncton with ConPhyMP 2022 explanaton and elaboraton for important clarifications on all items. If relevant, we also recommend after reading Heinrich et al. (2020) Best practice in research—Overcoming common challenges in phytopharmacological research. Journal of Ethnopharmacology. 2020;246:112230. https://doi.org/10.1016/j.jep.2019.112230

ConPhyMP checklist of items for conducting and reporting analytical methods[1,2] relevant for extract type A (for species or botanical drugs covered in a monograph in one of the national or regional pharmacopoeias)

SECTION/TOPIC	ITEM NO.	CHECKLIST ITEM	YES	NO	NOT APPLICABLE	PAGE NO., IF ANY
Type of extract	1	A – Confirm that the species or botanical drug under investigation is covered in a monograph in one of the national or regional pharmacopoeias.	✓			
Preferred/main methods for extract characterisation/ chemical analysis	2	Compliance with pharmacopoeial standards to be followed:				
		(a) The description of the active ingredients in the botanical drug (if known) or analytical marker compounds as defined.	✓			
		(b) An analysis as defined in the monograph is needed if the extract has not been supplied with a certificate.	✓			
		(c) If the preparation was purchased, the manufacturer and certificate of analysis need to be included.	✓			
		Including either the preferred or alternative approaches for characterisation:				
		(a) Triple chemical fingerprinting methods, each with one or more detection parameters.		✓		
		(b) Quantification of at least two marker compounds (unless this is not feasible, evidence needs to be provided), and justification of the choice of markers (if applicable).	✓			
Alternative methods for extract characterisation/chemical analysis	3	(a) Single chemical fingerprinting method with at least three different detection parameters (i.e., altered detection parameters, like TLC/HPTLC with different staining reagents and/or UV excitation wave-lengths, HPLC-DAD/LCDAD with different wavelengths). The same applies to coupling MS or NMR to chromatographic techniques.		✓		
		(b) Quantification of at least two marker compounds (unless this is not feasible, evidence needs to be provided), and justification of the choice of markers (if applicable).	✓			
Use of reference standards	4	(a) Direct overlay of the chromatogram of the sample with that of an officially specified reference standard (if applicable).		✓		
		(b) Chromatographic fingerprinting: Direct overlay of the chromato-gram of the sample with that of official reference standards of the powdered plant material or the dry extract from the plant material.				
Comparison of different extracts/ samples of the same plants	5	(a) Direct comparison of the chromatographic/spectroscopic system and/or scoring system for "similarity" to be followed.			✓	

Note: Please also include here the following information about your submitted manuscript:

Name of the journal:	F1000Research
Date of the enquiry:	2024 Apr
Title of the manuscript:	e, randomized, double-blind, placebo-controlled study
List of the authors:	yali Patil 3, Shashank Tiwari 1, Ashok Kumar Mishra 4

[1] Please acknowledge/cite this as follows: Heinrich M, Jalil B, Abdel-Tawab M, Echeverria J, Kulić Ž, McGaw LJ, et al. Best Practice in the chemical characterisation of extracts used in pharmacological and toxicological research—The ConPhyMP—Guidelines. Frontiers in Pharmacology. 2022;13:953205. https://doi.org/10.3389/fphar.2022.953205

[2] We strongly recommend reading this checklist in conjuncton with ConPhyMP 2022 explanaton and elaboraton for important clarifications on all items. If relevant, we also recommend after reading Heinrich et al. (2020) Best practice in research—Overcoming common challenges in phytopharmacological research. Journal of Ethnopharmacology. 2020;246:112230. https://doi.org/10.1016/j.jep.2019.112230

ConPhyMP checklist of information for reporting plant material and its initial processing [1,2] (relevant for all studies on medicinal and food plants including extract types A, B, and C)

SECTION/TOPIC	ITEM NO.	CHECKLIST ITEM	YES	NO	NOT APPLICABLE	PAGE NO., IF ANY
Title and abstract	1	A clear and concise title including an informative abstract and balanced summary.	✓			
Description of the botanical drug and taxonomic authentication	2	Botanical or morphological authentication of the plant material (desirable is a combination with DNA barcoding, e.g., PCR, RFLP, genome sequencing) and the information must be included in a separate section of Material and Methods, if applicable, combined with the information required under item 3:	✓			
Description of the extract and extraction process	3	A separate section in Material and Methods, covers the relevant information on the material investigated, including the full species name(s), authorites and family; e.g. *Salvia miltorrhiza* Bunge [Lamiaceae; *Salviae miltorrhizae radix et rhizoma*], and on the processing and extraction of the crude drug including the traditional processing of the material used medicinally (fumigation, steaming, roasting, cooking, frying, etc.).	✓			
Documentation of the legal basis for collection and processing	4	Full compliance with the Nagoya protocol, CITES, and all associated treaties including phytosanitary regulations.			✓	
Description of product characteristics, in case of a finished (commercial) product	5	Information on the characteristics of the commercial products including batch number and date of production/best by information and regulatory status.		✓		

Note: Please also include here the following information about your submitted manuscript:

Name of the journal:	Medicine
Date of the enquiry:	2019 Sep
Title of the manuscript:	A randomized, double-blind, placebo-controlled study
List of the authors:	phen J Smith 1 2, Hakeemudin Malvi 3, Rahul Kodgule 4

[1] Please acknowledge/cite this as follows: Heinrich M, Jalil B, Abdel-Tawab M, Echeverria J, Kulić Ž, McGaw LJ, et al. Best Practice in the chemical characterisation of extracts used in pharmacological and toxicological research—The ConPhyMP—Guidelines. Frontiers in Pharmacology. 2022;13:953205. https://doi.org/10.3389/fphar.2022.953205

[2] We strongly recommend reading this checklist in conjuncton with ConPhyMP 2022 explanaton and elaboraton for important clarifications on all items. If relevant, we also recommend after reading Heinrich et al. (2020) Best practice in research—Overcoming common challenges in phytopharmacological research. Journal of Ethnopharmacology. 2020;246:112230. https://doi.org/10.1016/j.jep.2019.112230

ConPhyMP checklist of items for conducting and reporting analytical methods[1,2] relevant for extract type A (for species or botanical drugs covered in a monograph in one of the national or regional pharmacopoeias)

SECTION/TOPIC	ITEM NO.	CHECKLIST ITEM	YES	NO	NOT APPLICABLE	PAGE NO., IF ANY
Type of extract	1	A – Confirm that the species or botanical drug under investigation is covered in a monograph in one of the national or regional pharmacopoeias.	✓			
Preferred/main methods for extract characterisation/ chemical analysis	2	Compliance with pharmacopoeial standards to be followed:				
		(a) The description of the active ingredients in the botanical drug (if known) or analytical marker compounds as defined.	✓			
		(b) An analysis as defined in the monograph is needed if the extract has not been supplied with a certificate.	✓			
		(c) If the preparation was purchased, the manufacturer and certificate of analysis need to be included.	✓			
		Including either the preferred or alternative approaches for characterisation:				
		(a) Triple chemical fingerprinting methods, each with one or more detection parameters.		✓		
		(b) Quantification of at least two marker compounds (unless this is not feasible, evidence needs to be provided), and justification of the choice of markers (if applicable).	✓			
Alternative methods for extract characterisation/chemical analysis	3	(a) Single chemical fingerprinting method with at least three different detection parameters (i.e., altered detection parameters, like TLC/HPTLC with different staining reagents and/or UV excitation wave-lengths, HPLC-DAD/LCDAD with different wavelengths). The same applies to coupling MS or NMR to chromatographic techniques.		✓		
		(b) Quantification of at least two marker compounds (unless this is not feasible, evidence needs to be provided), and justification of the choice of markers (if applicable).	✓			
Use of reference standards	4	(a) Direct overlay of the chromatogram of the sample with that of an officially specified reference standard (if applicable). (b) Chromatographic fingerprinting: Direct overlay of the chromato-gram of the sample with that of official reference standards of the powdered plant material or the dry extract from the plant material.		✓		
Comparison of different extracts/ samples of the same plants	5	(a) Direct comparison of the chromatographic/spectroscopic system and/or scoring system for "similarity" to be followed.			✓	

Note: Please also include here the following information about your submitted manuscript:

Name of the journal:	Medicine
Date of the enquiry:	2019 Sep
Title of the manuscript:	A randomized, double-blind, placebo-controlled study
List of the authors:	phen J Smith 1 2, Hakeemudin Malvi 3, Rahul Kodgule 4

[1] Please acknowledge/cite this as follows: Heinrich M, Jalil B, Abdel-Tawab M, Echeverria J, Kulić Ž, McGaw LJ, et al. Best Practice in the chemical characterisation of extracts used in pharmacological and toxicological research—The ConPhyMP—Guidelines. Frontiers in Pharmacology. 2022;13:953205. https://doi.org/10.3389/fphar.2022.953205

[2] We strongly recommend reading this checklist in conjuncton with ConPhyMP 2022 explanaton and elaboraton for important clarifications on all items. If relevant, we also recommend after reading Heinrich et al. (2020) Best practice in research—Overcoming common challenges in phytopharmacological research. Journal of Ethnopharmacology. 2020;246:112230. https://doi.org/10.1016/j.jep.2019.112230

ConPhyMP checklist of information for reporting plant material and its initial processing [1,2] (relevant for all studies on medicinal and food plants including extract types A, B, and C)

SECTION/TOPIC	ITEM NO.	CHECKLIST ITEM	YES	NO	NOT APPLICABLE	PAGE NO., IF ANY
Title and abstract	1	A clear and concise title including an informative abstract and balanced summary.	✓			
Description of the botanical drug and taxonomic authentication	2	Botanical or morphological authentication of the plant material (desirable is a combination with DNA barcoding, e.g., PCR, RFLP, genome sequencing) and the information must be included in a separate section of Material and Methods, if applicable, combined with the information required under item 3:	✓			
Description of the extract and extraction process	3	A separate section in Material and Methods, covers the relevant information on the material investigated, including the full species name(s), authorites and family; e.g. *Salvia miltorrhiza* Bunge [Lamiaceae; *Salviae miltorrhizae radix et rhizoma*], and on the processing and extraction of the crude drug including the traditional processing of the material used medicinally (fumigation, steaming, roasting, cooking, frying, etc.).	✓			
Documentation of the legal basis for collection and processing	4	Full compliance with the Nagoya protocol, CITES, and all associated treaties including phytosanitary regulations.			✓	
Description of product characteristics, in case of a finished (commercial) product	5	Information on the characteristics of the commercial products including batch number and date of production/best by information and regulatory status.	✓			

Note: Please also include here the following information about your submitted manuscript:

Name of the journal: American Journal of Men's Health

Date of the enquiry: 2019 Mar-Apr

Title of the manuscript: andha (Withania somnifera) in Aging, Overweight Males

List of the authors: Lopresti 1 2, Peter D Drummond 1, Stephen J Smith 1 2

[1] Please acknowledge/cite this as follows: Heinrich M, Jalil B, Abdel-Tawab M, Echeverria J, Kulić Ž, McGaw LJ, et al. Best Practice in the chemical characterisation of extracts used in pharmacological and toxicological research—The ConPhyMP—Guidelines. Frontiers in Pharmacology. 2022;13:953205. https://doi.org/10.3389/fphar.2022.953205

[2] We strongly recommend reading this checklist in conjuncton with ConPhyMP 2022 explanaton and elaboraton for important clarifications on all items. If relevant, we also recommend after reading Heinrich et al. (2020) Best practice in research—Overcoming common challenges in phytopharmacological research. Journal of Ethnopharmacology. 2020;246:112230. https://doi.org/10.1016/j.jep.2019.112230

ConPhyMP checklist of items for conducting and reporting analytical methods[1,2] relevant for extract type A (for species or botanical drugs covered in a monograph in one of the national or regional pharmacopoeias)

SECTION/TOPIC	ITEM NO.	CHECKLIST ITEM	YES	NO	NOT APPLICABLE	PAGE NO., IF ANY
Type of extract	1	A – Confirm that the species or botanical drug under investigation is covered in a monograph in one of the national or regional pharmacopoeias.	✓			
Preferred/main methods for extract characterisation/ chemical analysis	2	Compliance with pharmacopoeial standards to be followed:				
		(a) The description of the active ingredients in the botanical drug (if known) or analytical marker compounds as defined.	✓			
		(b) An analysis as defined in the monograph is needed if the extract has not been supplied with a certificate.	✓			
		(c) If the preparation was purchased, the manufacturer and certificate of analysis need to be included.	✓			
		Including either the preferred or alternative approaches for characterisation:				
		(a) Triple chemical fingerprinting methods, each with one or more detection parameters.		✓		
		(b) Quantification of at least two marker compounds (unless this is not feasible, evidence needs to be provided), and justification of the choice of markers (if applicable).	✓			
Alternative methods for extract characterisation/chemical analysis	3	(a) Single chemical fingerprinting method with at least three different detection parameters (i.e., altered detection parameters, like TLC/ HPTLC with different staining reagents and/or UV excitation wave-lengths, HPLC-DAD/LCDAD with different wavelengths). The same applies to coupling MS or NMR to chromatographic techniques.				
		(b) Quantification of at least two marker compounds (unless this is not feasible, evidence needs to be provided), and justification of the choice of markers (if applicable).	✓			
Use of reference standards	4	(a) Direct overlay of the chromatogram of the sample with that of an officially specified reference standard (if applicable).		✓		
		(b) Chromatographic fingerprinting: Direct overlay of the chromato-gram of the sample with that of official reference standards of the powdered plant material or the dry extract from the plant material.				
Comparison of different extracts/ samples of the same plants	5	(a) Direct comparison of the chromatographic/spectroscopic system and/or scoring system for "similarity" to be followed.			✓	

Note: Please also include here the following information about your submitted manuscript:

Name of the journal:	American Journal of Men's Health
Date of the enquiry:	2019 Mar-Apr
Title of the manuscript:	andha (Withania somnifera) in Aging, Overweight Males
List of the authors:	Lopresti 1 2, Peter D Drummond 1, Stephen J Smith 1 2

[1] Please acknowledge/cite this as follows: Heinrich M, Jalil B, Abdel-Tawab M, Echeverria J, Kulić Ž, McGaw LJ, et al. Best Practice in the chemical characterisation of extracts used in pharmacological and toxicological research—The ConPhyMP—Guidelines. Frontiers in Pharmacology. 2022;13:953205. https://doi.org/10.3389/fphar.2022.953205

[2] We strongly recommend reading this checklist in conjuncton with ConPhyMP 2022 explanaton and elaboraton for important clarifications on all items. If relevant, we also recommend after reading Heinrich et al. (2020) Best practice in research—Overcoming common challenges in phytopharmacological research. Journal of Ethnopharmacology. 2020;246:112230. https://doi.org/10.1016/j.jep.2019.112230

ConPhyMP checklist of information for reporting plant material and its initial processing [1,2] (relevant for all studies on medicinal and food plants including extract types A, B, and C)

SECTION/TOPIC	ITEM NO.	CHECKLIST ITEM	YES	NO	NOT APPLICABLE	PAGE NO., IF ANY
Title and abstract	1	A clear and concise title including an informative abstract and balanced summary.	✓			
Description of the botanical drug and taxonomic authentication	2	Botanical or morphological authentication of the plant material (desirable is a combination with DNA barcoding, e.g., PCR, RFLP, genome sequencing) and the information must be included in a separate section of Material and Methods, if applicable, combined with the information required under item 3:	✓			
Description of the extract and extraction process	3	A separate section in Material and Methods, covers the relevant information on the material investigated, including the full species name(s), authorites and family; e.g. *Salvia miltorrhiza* Bunge [Lamiaceae; *Salviae miltorrhizae radix et rhizoma*], and on the processing and extraction of the crude drug including the traditional processing of the material used medicinally (fumigation, steaming, roasting, cooking, frying, etc.).	✓			
Documentation of the legal basis for collection and processing	4	Full compliance with the Nagoya protocol, CITES, and all associated treaties including phytosanitary regulations.			✓	
Description of product characteristics, in case of a finished (commercial) product	5	Information on the characteristics of the commercial products including batch number and date of production/best by information and regulatory status.	✓			

Note: Please also include here the following information about your submitted manuscript:

Name of the journal:	Journal of Dietary Supplements
Date of the enquiry:	2017 Nov 2
Title of the manuscript:	t extract in improving memory and cognitive functions
List of the authors:	yanraj Choudhary , Sauvik Bhattacharyya , Sekhar Bose

[1] Please acknowledge/cite this as follows: Heinrich M, Jalil B, Abdel-Tawab M, Echeverria J, Kulić Ž, McGaw LJ, et al. Best Practice in the chemical characterisation of extracts used in pharmacological and toxicological research—The ConPhyMP—Guidelines. Frontiers in Pharmacology. 2022;13:953205. https://doi.org/10.3389/fphar.2022.953205

[2] We strongly recommend reading this checklist in conjuncton with ConPhyMP 2022 explanaton and elaboraton for important clarifications on all items. If relevant, we also recommend after reading Heinrich et al. (2020) Best practice in research—Overcoming common challenges in phytopharmacological research. Journal of Ethnopharmacology. 2020;246:112230. https://doi.org/10.1016/j.jep.2019.112230

ConPhyMP checklist of items for conducting and reporting analytical methods[1,2] relevant for extract type A (for species or botanical drugs covered in a monograph in one of the national or regional pharmacopoeias)

SECTION/TOPIC	ITEM NO.	CHECKLIST ITEM	YES	NO	NOT APPLICABLE	PAGE NO., IF ANY
Type of extract	1	A – Confirm that the species or botanical drug under investigation is covered in a monograph in one of the national or regional pharmacopoeias.	✓			
Preferred/main methods for extract characterisation/ chemical analysis	2	Compliance with pharmacopoeial standards to be followed:				
		(a) The description of the active ingredients in the botanical drug (if known) or analytical marker compounds as defined.	✓			
		(b) An analysis as defined in the monograph is needed if the extract has not been supplied with a certificate.	✓			
		(c) If the preparation was purchased, the manufacturer and certificate of analysis need to be included.	✓			
		Including either the preferred or alternative approaches for characterisation:				
		(a) Triple chemical fingerprinting methods, each with one or more detection parameters.		✓		
		(b) Quantification of at least two marker compounds (unless this is not feasible, evidence needs to be provided), and justification of the choice of markers (if applicable).	✓			
Alternative methods for extract characterisation/chemical analysis	3	(a) Single chemical fingerprinting method with at least three different detection parameters (i.e., altered detection parameters, like TLC/ HPTLC with different staining reagents and/or UV excitation wave-lengths, HPLC-DAD/LCDAD with different wavelengths). The same applies to coupling MS or NMR to chromatographic techniques.		✓		
		(b) Quantification of at least two marker compounds (unless this is not feasible, evidence needs to be provided), and justification of the choice of markers (if applicable).	✓			
Use of reference standards	4	(a) Direct overlay of the chromatogram of the sample with that of an officially specified reference standard (if applicable).	✓			
		(b) Chromatographic fingerprinting: Direct overlay of the chromato-gram of the sample with that of official reference standards of the powdered plant material or the dry extract from the plant material.				
Comparison of different extracts/ samples of the same plants	5	(a) Direct comparison of the chromatographic/spectroscopic system and/or scoring system for "similarity" to be followed.			✓	

Note: Please also include here the following information about your submitted manuscript:

Name of the journal:	Journal of Dietary Supplements
Date of the enquiry:	2017 Nov 2
Title of the manuscript:	t extract in improving memory and cognitive functions
List of the authors:	Dnyanraj Choudhary , Sauvik Bhattacharyya , Sekhar Bo

[1] Please acknowledge/cite this as follows: Heinrich M, Jalil B, Abdel-Tawab M, Echeverria J, Kulić Ž, McGaw LJ, et al. Best Practice in the chemical characterisation of extracts used in pharmacological and toxicological research—The ConPhyMP—Guidelines. Frontiers in Pharmacology. 2022;13:953205. https://doi.org/10.3389/fphar.2022.953205

[2] We strongly recommend reading this checklist in conjuncton with ConPhyMP 2022 explanaton and elaboraton for important clarifications on all items. If relevant, we also recommend after reading Heinrich et al. (2020) Best practice in research—Overcoming common challenges in phytopharmacological research. Journal of Ethnopharmacology. 2020;246:112230. https://doi.org/10.1016/j.jep.2019.112230

ConPhyMP checklist of information for reporting plant material and its initial processing [1,2] (relevant for all studies on medicinal and food plants including extract types A, B, and C)

SECTION/TOPIC	ITEM NO.	CHECKLIST ITEM	YES	NO	NOT APPLICABLE	PAGE NO., IF ANY
Title and abstract	1	A clear and concise title including an informative abstract and balanced summary.	✓			
Description of the botanical drug and taxonomic authentication	2	Botanical or morphological authentication of the plant material (desirable is a combination with DNA barcoding, e.g., PCR, RFLP, genome sequencing) and the information must be included in a separate section of Material and Methods, if applicable, combined with the information required under item 3:	✓			
Description of the extract and extraction process	3	A separate section in Material and Methods, covers the relevant information on the material investigated, including the full species name(s), authorites and family; e.g. *Salvia miltorrhiza* Bunge [Lamiaceae; *Salviae miltorrhizae radix et rhizoma*], and on the processing and extraction of the crude drug including the traditional processing of the material used medicinally (fumigation, steaming, roasting, cooking, frying, etc.).	✓			
Documentation of the legal basis for collection and processing	4	Full compliance with the Nagoya protocol, CITES, and all associated treaties including phytosanitary regulations.			✓	
Description of product characteristics, in case of a finished (commercial) product	5	Information on the characteristics of the commercial products including batch number and date of production/best by information and regulatory status.	✓			

Note: Please also include here the following information about your submitted manuscript:

Name of the journal: rnal of the International Society of Sports Nutrition

Date of the enquiry: 2015 Nov

Title of the manuscript: strength and recovery: a randomized controlled trial

List of the authors: dar Joshi 3, Shymal R Sinha 4, Sauvik Bhattacharyya 5

[1] Please acknowledge/cite this as follows: Heinrich M, Jalil B, Abdel-Tawab M, Echeverria J, Kulić Ž, McGaw LJ, et al. Best Practice in the chemical characterisation of extracts used in pharmacological and toxicological research—The ConPhyMP—Guidelines. Frontiers in Pharmacology. 2022;13:953205. https://doi.org/10.3389/fphar.2022.953205

[2] We strongly recommend reading this checklist in conjuncton with ConPhyMP 2022 explanaton and elaboraton for important clarifications on all items. If relevant, we also recommend after reading Heinrich et al. (2020) Best practice in research—Overcoming common challenges in phytopharmacological research. Journal of Ethnopharmacology. 2020;246:112230. https://doi.org/10.1016/j.jep.2019.112230

ConPhyMP checklist of items for conducting and reporting analytical methods[1,2] relevant for extract type A (for species or botanical drugs covered in a monograph in one of the national or regional pharmacopoeias)

SECTION/TOPIC	ITEM NO.	CHECKLIST ITEM	YES	NO	NOT APPLICABLE	PAGE NO., IF ANY
Type of extract	1	A – Confirm that the species or botanical drug under investigation is covered in a monograph in one of the national or regional pharmacopoeias.	✓			
Preferred/main methods for extract characterisation/ chemical analysis	2	Compliance with pharmacopoeial standards to be followed:				
		(a) The description of the active ingredients in the botanical drug (if known) or analytical marker compounds as defined.	✓			
		(b) An analysis as defined in the monograph is needed if the extract has not been supplied with a certificate.	✓			
		(c) If the preparation was purchased, the manufacturer and certificate of analysis need to be included.	✓			
		Including either the preferred or alternative approaches for characterisation:				
		(a) Triple chemical fingerprinting methods, each with one or more detection parameters.		✓		
		(b) Quantification of at least two marker compounds (unless this is not feasible, evidence needs to be provided), and justification of the choice of markers (if applicable).	✓			
Alternative methods for extract characterisation/chemical analysis	3	(a) Single chemical fingerprinting method with at least three different detection parameters (i.e., altered detection parameters, like TLC/ HPTLC with different staining reagents and/or UV excitation wave-lengths, HPLC-DAD/LCDAD with different wavelengths). The same applies to coupling MS or NMR to chromatographic techniques.		✓		
		(b) Quantification of at least two marker compounds (unless this is not feasible, evidence needs to be provided), and justification of the choice of markers (if applicable).	✓			
Use of reference standards	4	(a) Direct overlay of the chromatogram of the sample with that of an officially specified reference standard (if applicable). (b) Chromatographic fingerprinting: Direct overlay of the chromato-gram of the sample with that of official reference standards of the powdered plant material or the dry extract from the plant material.		✓		
Comparison of different extracts/ samples of the same plants	5	(a) Direct comparison of the chromatographic/spectroscopic system and/or scoring system for "similarity" to be followed.			✓	

Note: Please also include here the following information about your submitted manuscript:

Name of the journal:	rnal of the International Society of Sports Nutrition
Date of the enquiry:	2015 Nov
Title of the manuscript:	strength and recovery: a randomized controlled trial
List of the authors:	dar Joshi 3, Shymal R Sinha 4, Sauvik Bhattacharyya 5

[1] Please acknowledge/cite this as follows: Heinrich M, Jalil B, Abdel-Tawab M, Echeverria J, Kulić Ž, McGaw LJ, et al. Best Practice in the chemical characterisation of extracts used in pharmacological and toxicological research—The ConPhyMP—Guidelines. Frontiers in Pharmacology. 2022;13:953205. https://doi.org/10.3389/fphar.2022.953205

[2] We strongly recommend reading this checklist in conjuncton with ConPhyMP 2022 explanaton and elaboraton for important clarifications on all items. If relevant, we also recommend after reading Heinrich et al. (2020) Best practice in research—Overcoming common challenges in phytopharmacological research. Journal of Ethnopharmacology. 2020;246:112230. https://doi.org/10.1016/j.jep.2019.112230

ConPhyMP checklist of information for reporting plant material and its initial processing [1,2] (relevant for all studies on medicinal and food plants including extract types A, B, and C)

SECTION/TOPIC	ITEM NO.	CHECKLIST ITEM	YES	NO	NOT APPLICABLE	PAGE NO., IF ANY
Title and abstract	1	A clear and concise title including an informative abstract and balanced summary.	✓			
Description of the botanical drug and taxonomic authentication	2	Botanical or morphological authentication of the plant material (desirable is a combination with DNA barcoding, e.g., PCR, RFLP, genome sequencing) and the information must be included in a separate section of Material and Methods, if applicable, combined with the information required under item 3:	✓			
Description of the extract and extraction process	3	A separate section in Material and Methods, covers the relevant information on the material investigated, including the full species name(s), authorites and family; e.g. *Salvia miltorrhiza* Bunge [Lamiaceae; *Salviae miltorrhizae radix et rhizoma*], and on the processing and extraction of the crude drug including the traditional processing of the material used medicinally (fumigation, steaming, roasting, cooking, frying, etc.).	✓			
Documentation of the legal basis for collection and processing	4	Full compliance with the Nagoya protocol, CITES, and all associated treaties including phytosanitary regulations.			✓	
Description of product characteristics, in case of a finished (commercial) product	5	Information on the characteristics of the commercial products including batch number and date of production/best by information and regulatory status.	✓			

Note: Please also include here the following information about your submitted manuscript:

Name of the journal: Journal of Clinical Psychiatry

Date of the enquiry: 2013 Nov

Title of the manuscript: mnifera for cognitive dysfunction in bipolar disorder

List of the authors: J Schlicht, David Fleet, Jaspreet S Brar, Ripu Jindal

[1] Please acknowledge/cite this as follows: Heinrich M, Jalil B, Abdel-Tawab M, Echeverria J, Kulić Ž, McGaw LJ, et al. Best Practice in the chemical characterisation of extracts used in pharmacological and toxicological research—The ConPhyMP—Guidelines. Frontiers in Pharmacology. 2022;13:953205. https://doi.org/10.3389/fphar.2022.953205

[2] We strongly recommend reading this checklist in conjuncton with ConPhyMP 2022 explanaton and elaboraton for important clarifications on all items. If relevant, we also recommend after reading Heinrich et al. (2020) Best practice in research—Overcoming common challenges in phytopharmacological research. Journal of Ethnopharmacology. 2020;246:112230. https://doi.org/10.1016/j.jep.2019.112230

ConPhyMP checklist of items for conducting and reporting analytical methods[1,2] relevant for extract type A (for species or botanical drugs covered in a monograph in one of the national or regional pharmacopoeias)

SECTION/TOPIC	ITEM NO.	CHECKLIST ITEM	YES	NO	NOT APPLICABLE	PAGE NO., IF ANY
Type of extract	1	A – Confirm that the species or botanical drug under investigation is covered in a monograph in one of the national or regional pharmacopoeias.	✓			
Preferred/main methods for extract characterisation/ chemical analysis	2	Compliance with pharmacopoeial standards to be followed:				
		(a) The description of the active ingredients in the botanical drug (if known) or analytical marker compounds as defined.	✓			
		(b) An analysis as defined in the monograph is needed if the extract has not been supplied with a certificate.				
		(c) If the preparation was purchased, the manufacturer and certificate of analysis need to be included.	✓			
		Including either the preferred or alternative approaches for characterisation:				
		(a) Triple chemical fingerprinting methods, each with one or more detection parameters.		✓		
		(b) Quantification of at least two marker compounds (unless this is not feasible, evidence needs to be provided), and justification of the choice of markers (if applicable).	✓			
Alternative methods for extract characterisation/chemical analysis	3	(a) Single chemical fingerprinting method with at least three different detection parameters (i.e., altered detection parameters, like TLC/HPTLC with different staining reagents and/or UV excitation wave-lengths, HPLC-DAD/LCDAD with different wavelengths). The same applies to coupling MS or NMR to chromatographic techniques.		✓		
		(b) Quantification of at least two marker compounds (unless this is not feasible, evidence needs to be provided), and justification of the choice of markers (if applicable).	✓			
Use of reference standards	4	(a) Direct overlay of the chromatogram of the sample with that of an officially specified reference standard (if applicable).<br>(b) Chromatographic fingerprinting: Direct overlay of the chromato-gram of the sample with that of official reference standards of the powdered plant material or the dry extract from the plant material.		✓		
Comparison of different extracts/ samples of the same plants	5	(a) Direct comparison of the chromatographic/spectroscopic system and/or scoring system for "similarity" to be followed.			✓	

Note: Please also include here the following information about your submitted manuscript:

Name of the journal: Journal of Clinical Psychiatry

Date of the enquiry: 2013 Nov

Title of the manuscript: mnifera for cognitive dysfunction in bipolar disorder

List of the authors: J Schlicht, David Fleet, Jaspreet S Brar, Ripu Jindal

[1] Please acknowledge/cite this as follows: Heinrich M, Jalil B, Abdel-Tawab M, Echeverria J, Kulić Ž, McGaw LJ, et al. Best Practice in the chemical characterisation of extracts used in pharmacological and toxicological research—The ConPhyMP—Guidelines. Frontiers in Pharmacology. 2022;13:953205. https://doi.org/10.3389/fphar.2022.953205

[2] We strongly recommend reading this checklist in conjuncton with ConPhyMP 2022 explanaton and elaboraton for important clarifications on all items. If relevant, we also recommend after reading Heinrich et al. (2020) Best practice in research—Overcoming common challenges in phytopharmacological research. Journal of Ethnopharmacology. 2020;246:112230. https://doi.org/10.1016/j.jep.2019.112230

ConPhyMP checklist of information for reporting plant material and its initial processing [1,2] (relevant for all studies on medicinal and food plants including extract types A, B, and C)

SECTION/TOPIC	ITEM NO.	CHECKLIST ITEM	YES	NO	NOT APPLICABLE	PAGE NO., IF ANY
Title and abstract	1	A clear and concise title including an informative abstract and balanced summary.	✓			
Description of the botanical drug and taxonomic authentication	2	Botanical or morphological authentication of the plant material (desirable is a combination with DNA barcoding, e.g., PCR, RFLP, genome sequencing) and the information must be included in a separate section of Material and Methods, if applicable, combined with the information required under item 3:		✓		
Description of the extract and extraction process	3	A separate section in Material and Methods, covers the relevant information on the material investigated, including the full species name(s), authorites and family; e.g. *Salvia miltorrhiza* Bunge [Lamiaceae; *Salviae miltorrhizae radix et rhizoma*], and on the processing and extraction of the crude drug including the traditional processing of the material used medicinally (fumigation, steaming, roasting, cooking, frying, etc.).		✓		
Documentation of the legal basis for collection and processing	4	Full compliance with the Nagoya protocol, CITES, and all associated treaties including phytosanitary regulations.			✓	
Description of product characteristics, in case of a finished (commercial) product	5	Information on the characteristics of the commercial products including batch number and date of production/best by information and regulatory status.		✓		

Note: Please also include here the following information about your submitted manuscript:

Name of the journal: Journal of Psychopharmacology

Date of the enquiry: 2023 Nov

Title of the manuscript: a randomized, double-blind, placebo-controlled trial

List of the authors: ith 1 2, Adrian L Lopresti 1 2, Timothy J Fairchild 2

[1] Please acknowledge/cite this as follows: Heinrich M, Jalil B, Abdel-Tawab M, Echeverria J, Kulić Ž, McGaw LJ, et al. Best Practice in the chemical characterisation of extracts used in pharmacological and toxicological research—The ConPhyMP—Guidelines. Frontiers in Pharmacology. 2022;13:953205. https://doi.org/10.3389/fphar.2022.953205

[2] We strongly recommend reading this checklist in conjuncton with ConPhyMP 2022 explanaton and elaboraton for important clarifications on all items. If relevant, we also recommend after reading Heinrich et al. (2020) Best practice in research—Overcoming common challenges in phytopharmacological research. Journal of Ethnopharmacology. 2020;246:112230. https://doi.org/10.1016/j.jep.2019.112230

ConPhyMP checklist of items for conducting and reporting analytical methods[1,2] relevant for extract type A (for species or botanical drugs covered in a monograph in one of the national or regional pharmacopoeias)

SECTION/TOPIC	ITEM NO.	CHECKLIST ITEM	YES	NO	NOT APPLICABLE	PAGE NO., IF ANY
Type of extract	1	A – Confirm that the species or botanical drug under investigation is covered in a monograph in one of the national or regional pharmacopoeias.	✓			
Preferred/main methods for extract characterisation/ chemical analysis	2	Compliance with pharmacopoeial standards to be followed:				
		(a) The description of the active ingredients in the botanical drug (if known) or analytical marker compounds as defined.		✓		
		(b) An analysis as defined in the monograph is needed if the extract has not been supplied with a certificate.		✓		
		(c) If the preparation was purchased, the manufacturer and certificate of analysis need to be included.		✓		
		Including either the preferred or alternative approaches for characterisation:				
		(a) Triple chemical fingerprinting methods, each with one or more detection parameters.		✓		
		(b) Quantification of at least two marker compounds (unless this is not feasible, evidence needs to be provided), and justification of the choice of markers (if applicable).		✓		
Alternative methods for extract characterisation/chemical analysis	3	(a) Single chemical fingerprinting method with at least three different detection parameters (i.e., altered detection parameters, like TLC/ HPTLC with different staining reagents and/or UV excitation wave-lengths, HPLC-DAD/LCDAD with different wavelengths). The same applies to coupling MS or NMR to chromatographic techniques.		✓		
		(b) Quantification of at least two marker compounds (unless this is not feasible, evidence needs to be provided), and justification of the choice of markers (if applicable).		✓		
Use of reference standards	4	(a) Direct overlay of the chromatogram of the sample with that of an officially specified reference standard (if applicable). (b) Chromatographic fingerprinting: Direct overlay of the chromato-gram of the sample with that of official reference standards of the powdered plant material or the dry extract from the plant material.		✓		
Comparison of different extracts/ samples of the same plants	5	(a) Direct comparison of the chromatographic/spectroscopic system and/or scoring system for "similarity" to be followed.			✓	

Note: Please also include here the following information about your submitted manuscript:

Name of the journal:	Journal of Psychopharmacology
Date of the enquiry:	2023 Nov
Title of the manuscript:	a randomized, double-blind, placebo-controlled trial
List of the authors:	ith 1 2, Adrian L Lopresti 1 2, Timothy J Fairchild 2

[1] Please acknowledge/cite this as follows: Heinrich M, Jalil B, Abdel-Tawab M, Echeverria J, Kulić Ž, McGaw LJ, et al. Best Practice in the chemical characterisation of extracts used in pharmacological and toxicological research—The ConPhyMP—Guidelines. Frontiers in Pharmacology. 2022;13:953205. https://doi.org/10.3389/fphar.2022.953205

[2] We strongly recommend reading this checklist in conjuncton with ConPhyMP 2022 explanaton and elaboraton for important clarifications on all items. If relevant, we also recommend after reading Heinrich et al. (2020) Best practice in research—Overcoming common challenges in phytopharmacological research. Journal of Ethnopharmacology. 2020;246:112230. https://doi.org/10.1016/j.jep.2019.112230

ConPhyMP checklist of information for reporting plant material and its initial processing [1,2] (relevant for all studies on medicinal and food plants including extract types A, B, and C)

SECTION/TOPIC	ITEM NO.	CHECKLIST ITEM	YES	NO	NOT APPLICABLE	PAGE NO., IF ANY
Title and abstract	1	A clear and concise title including an informative abstract and balanced summary.	○	○	○	
Description of the botanical drug and taxonomic authentication	2	Botanical or morphological authentication of the plant material (desirable is a combination with DNA barcoding, e.g., PCR, RFLP, genome sequencing) and the information must be included in a separate section of Material and Methods, if applicable, combined with the information required under item 3:	✓	○	○	
Description of the extract and extraction process	3	A separate section in Material and Methods, covers the relevant information on the material investigated, including the full species name(s), authorites and family; e.g. *Salvia miltorrhiza* Bunge [Lamiaceae; *Salviae miltorrhizae radix et rhizoma*], and on the processing and extraction of the crude drug including the traditional processing of the material used medicinally (fumigation, steaming, roasting, cooking, frying, etc.).	✓	○	○	
Documentation of the legal basis for collection and processing	4	Full compliance with the Nagoya protocol, CITES, and all associated treaties including phytosanitary regulations.	○	○	✓	
Description of product characteristics, in case of a finished (commercial) product	5	Information on the characteristics of the commercial products including batch number and date of production/best by information and regulatory status.	✓	○	○	

Note: Please also include here the following information about your submitted manuscript:

Name of the journal: Journal of Ayurveda and Integrative Medicine

Date of the enquiry: 2025 Jun 28

Title of the manuscript: e, randomized, double-blind, placebo-controlled study

List of the authors: ant Beldar 3, Alok Mahendra Mulay 4, Deepak Langade 5

[1] Please acknowledge/cite this as follows: Heinrich M, Jalil B, Abdel-Tawab M, Echeverria J, Kulić Ž, McGaw LJ, et al. Best Practice in the chemical characterisation of extracts used in pharmacological and toxicological research—The ConPhyMP—Guidelines. Frontiers in Pharmacology. 2022;13:953205. https://doi.org/10.3389/fphar.2022.953205

[2] We strongly recommend reading this checklist in conjuncton with ConPhyMP 2022 explanaton and elaboraton for important clarifications on all items. If relevant, we also recommend after reading Heinrich et al. (2020) Best practice in research—Overcoming common challenges in phytopharmacological research. Journal of Ethnopharmacology. 2020;246:112230. https://doi.org/10.1016/j.jep.2019.112230

ConPhyMP checklist of items for conducting and reporting analytical methods[1,2] relevant for extract type A (for species or botanical drugs covered in a monograph in one of the national or regional pharmacopoeias)

SECTION/TOPIC	ITEM NO.	CHECKLIST ITEM	YES	NO	NOT APPLICABLE	PAGE NO., IF ANY
Type of extract	1	A – Confirm that the species or botanical drug under investigation is covered in a monograph in one of the national or regional pharmacopoeias.	✓			
Preferred/main methods for extract characterisation/ chemical analysis	2	Compliance with pharmacopoeial standards to be followed:				
		(a) The description of the active ingredients in the botanical drug (if known) or analytical marker compounds as defined.	✓			
		(b) An analysis as defined in the monograph is needed if the extract has not been supplied with a certificate.	✓			
		(c) If the preparation was purchased, the manufacturer and certificate of analysis need to be included.	✓			
		Including either the preferred or alternative approaches for characterisation:				
		(a) Triple chemical fingerprinting methods, each with one or more detection parameters.		✓		
		(b) Quantification of at least two marker compounds (unless this is not feasible, evidence needs to be provided), and justification of the choice of markers (if applicable).		✓		
Alternative methods for extract characterisation/chemical analysis	3	(a) Single chemical fingerprinting method with at least three different detection parameters (i.e., altered detection parameters, like TLC/ HPTLC with different staining reagents and/or UV excitation wave-lengths, HPLC-DAD/LCDAD with different wavelengths). The same applies to coupling MS or NMR to chromatographic techniques.		✓		
		(b) Quantification of at least two marker compounds (unless this is not feasible, evidence needs to be provided), and justification of the choice of markers (if applicable).		✓		
Use of reference standards	4	(a) Direct overlay of the chromatogram of the sample with that of an officially specified reference standard (if applicable).		✓		
		(b) Chromatographic fingerprinting: Direct overlay of the chromato-gram of the sample with that of official reference standards of the powdered plant material or the dry extract from the plant material.				
Comparison of different extracts/ samples of the same plants	5	(a) Direct comparison of the chromatographic/spectroscopic system and/or scoring system for "similarity" to be followed.			✓	

Note: Please also include here the following information about your submitted manuscript:

Name of the journal:	Journal of Ayurveda and Integrative Medicine
Date of the enquiry:	2025 Jun 28
Title of the manuscript:	e, randomized, double-blind, placebo-controlled study
List of the authors:	ant Beldar 3, Alok Mahendra Mulay 4, Deepak Langade 5

[1] Please acknowledge/cite this as follows: Heinrich M, Jalil B, Abdel-Tawab M, Echeverria J, Kulić Ž, McGaw LJ, et al. Best Practice in the chemical characterisation of extracts used in pharmacological and toxicological research—The ConPhyMP—Guidelines. Frontiers in Pharmacology. 2022;13:953205. https://doi.org/10.3389/fphar.2022.953205

[2] We strongly recommend reading this checklist in conjuncton with ConPhyMP 2022 explanaton and elaboraton for important clarifications on all items. If relevant, we also recommend after reading Heinrich et al. (2020) Best practice in research—Overcoming common challenges in phytopharmacological research. Journal of Ethnopharmacology. 2020;246:112230. https://doi.org/10.1016/j.jep.2019.112230

ConPhyMP checklist of information for reporting plant material and its initial processing [1,2] (relevant for all studies on medicinal and food plants including extract types A, B, and C)

SECTION/TOPIC	ITEM NO.	CHECKLIST ITEM	YES	NO	NOT APPLICABLE	PAGE NO., IF ANY
Title and abstract	1	A clear and concise title including an informative abstract and balanced summary.	✓			
Description of the botanical drug and taxonomic authentication	2	Botanical or morphological authentication of the plant material (desirable is a combination with DNA barcoding, e.g., PCR, RFLP, genome sequencing) and the information must be included in a separate section of Material and Methods, if applicable, combined with the information required under item 3:		✓		
Description of the extract and extraction process	3	A separate section in Material and Methods, covers the relevant information on the material investigated, including the full species name(s), authorites and family; e.g. *Salvia miltorrhiza* Bunge [Lamiaceae; *Salviae miltorrhizae radix et rhizoma*], and on the processing and extraction of the crude drug including the traditional processing of the material used medicinally (fumigation, steaming, roasting, cooking, frying, etc.).		✓		
Documentation of the legal basis for collection and processing	4	Full compliance with the Nagoya protocol, CITES, and all associated treaties including phytosanitary regulations.			✓	
Description of product characteristics, in case of a finished (commercial) product	5	Information on the characteristics of the commercial products including batch number and date of production/best by information and regulatory status.	✓			

Note: Please also include here the following information about your submitted manuscript:

Name of the journal: f Evidence-Based Complementary & Alternative Medicine

Date of the enquiry: 2015 Nov 18

Title of the manuscript: A Double-Blind, Randomized, Placebo-Controlled Trial

List of the authors: Dnyanraj Choudhary, Sauvik Bhattacharyya, Kedar Joshi

[1] Please acknowledge/cite this as follows: Heinrich M, Jalil B, Abdel-Tawab M, Echeverria J, Kulić Ž, McGaw LJ, et al. Best Practice in the chemical characterisation of extracts used in pharmacological and toxicological research—The ConPhyMP—Guidelines. Frontiers in Pharmacology. 2022;13:953205. https://doi.org/10.3389/fphar.2022.953205

[2] We strongly recommend reading this checklist in conjuncton with ConPhyMP 2022 explanaton and elaboraton for important clarifications on all items. If relevant, we also recommend after reading Heinrich et al. (2020) Best practice in research—Overcoming common challenges in phytopharmacological research. Journal of Ethnopharmacology. 2020;246:112230. https://doi.org/10.1016/j.jep.2019.112230

ConPhyMP checklist of items for conducting and reporting analytical methods[1,2] relevant for extract type A (for species or botanical drugs covered in a monograph in one of the national or regional pharmacopoeias)

SECTION/TOPIC	ITEM NO.	CHECKLIST ITEM	YES	NO	NOT APPLICABLE	PAGE NO., IF ANY
Type of extract	1	A – Confirm that the species or botanical drug under investigation is covered in a monograph in one of the national or regional pharmacopoeias.	✓			
Preferred/main methods for extract characterisation/ chemical analysis	2	Compliance with pharmacopoeial standards to be followed:				
		(a) The description of the active ingredients in the botanical drug (if known) or analytical marker compounds as defined.	✓			
		(b) An analysis as defined in the monograph is needed if the extract has not been supplied with a certificate.		✓		
		(c) If the preparation was purchased, the manufacturer and certificate of analysis need to be included.		✓		
		Including either the preferred or alternative approaches for characterisation:				
		(a) Triple chemical fingerprinting methods, each with one or more detection parameters.		✓		
		(b) Quantification of at least two marker compounds (unless this is not feasible, evidence needs to be provided), and justification of the choice of markers (if applicable).		✓		
Alternative methods for extract characterisation/chemical analysis	3	(a) Single chemical fingerprinting method with at least three different detection parameters (i.e., altered detection parameters, like TLC/ HPTLC with different staining reagents and/or UV excitation wave-lengths, HPLC-DAD/LCDAD with different wavelengths). The same applies to coupling MS or NMR to chromatographic techniques.		✓		
		(b) Quantification of at least two marker compounds (unless this is not feasible, evidence needs to be provided), and justification of the choice of markers (if applicable).		✓		
Use of reference standards	4	(a) Direct overlay of the chromatogram of the sample with that of an officially specified reference standard (if applicable).<br>(b) Chromatographic fingerprinting: Direct overlay of the chromato-gram of the sample with that of official reference standards of the powdered plant material or the dry extract from the plant material.		✓		
Comparison of different extracts/ samples of the same plants	5	(a) Direct comparison of the chromatographic/spectroscopic system and/or scoring system for "similarity" to be followed.			✓	

Note: Please also include here the following information about your submitted manuscript:

Name of the journal:	f Evidence-Based Complementary & Alternative Medicine
Date of the enquiry:	2015 Nov 18
Title of the manuscript:	A Double-Blind, Randomized, Placebo-Controlled Trial
List of the authors:	Dnyanraj Choudhary, Sauvik Bhattacharyya, Kedar Joshi

[1] Please acknowledge/cite this as follows: Heinrich M, Jalil B, Abdel-Tawab M, Echeverria J, Kulić Ž, McGaw LJ, et al. Best Practice in the chemical characterisation of extracts used in pharmacological and toxicological research—The ConPhyMP—Guidelines. Frontiers in Pharmacology. 2022;13:953205. https://doi.org/10.3389/fphar.2022.953205

[2] We strongly recommend reading this checklist in conjuncton with ConPhyMP 2022 explanaton and elaboraton for important clarifications on all items. If relevant, we also recommend after reading Heinrich et al. (2020) Best practice in research—Overcoming common challenges in phytopharmacological research. Journal of Ethnopharmacology. 2020;246:112230. https://doi.org/10.1016/j.jep.2019.112230

ConPhyMP checklist of information for reporting plant material and its initial processing [1,2] (relevant for all studies on medicinal and food plants including extract types A, B, and C)

SECTION/TOPIC	ITEM NO.	CHECKLIST ITEM	YES	NO	NOT APPLICABLE	PAGE NO., IF ANY
Title and abstract	1	A clear and concise title including an informative abstract and balanced summary.	✓			
Description of the botanical drug and taxonomic authentication	2	Botanical or morphological authentication of the plant material (desirable is a combination with DNA barcoding, e.g., PCR, RFLP, genome sequencing) and the information must be included in a separate section of Material and Methods, if applicable, combined with the information required under item 3:		✓		
Description of the extract and extraction process	3	A separate section in Material and Methods, covers the relevant information on the material investigated, including the full species name(s), authorites and family; e.g. *Salvia miltorrhiza* Bunge [Lamiaceae; *Salviae miltorrhizae radix et rhizoma*], and on the processing and extraction of the crude drug including the traditional processing of the material used medicinally (fumigation, steaming, roasting, cooking, frying, etc.).		✓		
Documentation of the legal basis for collection and processing	4	Full compliance with the Nagoya protocol, CITES, and all associated treaties including phytosanitary regulations.			✓	
Description of product characteristics, in case of a finished (commercial) product	5	Information on the characteristics of the commercial products including batch number and date of production/best by information and regulatory status.	✓			

Note: Please also include here the following information about your submitted manuscript:

Name of the journal:	Journal of Ayurveda and Integrative Medicine
Date of the enquiry:	2021 November 25
Title of the manuscript:	mnifera supplementation on adult's cognition and mood
List of the authors:	D Hooper 1, S Hooper 1, K Conway 1, H A Hausenblas 2

[1] Please acknowledge/cite this as follows: Heinrich M, Jalil B, Abdel-Tawab M, Echeverria J, Kulić Ž, McGaw LJ, et al. Best Practice in the chemical characterisation of extracts used in pharmacological and toxicological research—The ConPhyMP—Guidelines. Frontiers in Pharmacology. 2022;13:953205. https://doi.org/10.3389/fphar.2022.953205

[2] We strongly recommend reading this checklist in conjuncton with ConPhyMP 2022 explanaton and elaboraton for important clarifications on all items. If relevant, we also recommend after reading Heinrich et al. (2020) Best practice in research—Overcoming common challenges in phytopharmacological research. Journal of Ethnopharmacology. 2020;246:112230. https://doi.org/10.1016/j.jep.2019.112230

ConPhyMP checklist of items for conducting and reporting analytical methods[1,2] relevant for extract type A (for species or botanical drugs covered in a monograph in one of the national or regional pharmacopoeias)

SECTION/TOPIC	ITEM NO.	CHECKLIST ITEM	YES	NO	NOT APPLICABLE	PAGE NO., IF ANY
Type of extract	1	A – Confirm that the species or botanical drug under investigation is covered in a monograph in one of the national or regional pharmacopoeias.	✓			
Preferred/main methods for extract characterisation/chemical analysis	2	Compliance with pharmacopoeial standards to be followed:				
		(a) The description of the active ingredients in the botanical drug (if known) or analytical marker compounds as defined.		✓		
		(b) An analysis as defined in the monograph is needed if the extract has not been supplied with a certificate.		✓		
		(c) If the preparation was purchased, the manufacturer and certificate of analysis need to be included.	✓			
		Including either the preferred or alternative approaches for characterisation:				
		(a) Triple chemical fingerprinting methods, each with one or more detection parameters.		✓		
		(b) Quantification of at least two marker compounds (unless this is not feasible, evidence needs to be provided), and justification of the choice of markers (if applicable).		✓		
Alternative methods for extract characterisation/chemical analysis	3	(a) Single chemical fingerprinting method with at least three different detection parameters (i.e., altered detection parameters, like TLC/HPTLC with different staining reagents and/or UV excitation wavelengths, HPLC-DAD/LCDAD with different wavelengths). The same applies to coupling MS or NMR to chromatographic techniques.		✓		
		(b) Quantification of at least two marker compounds (unless this is not feasible, evidence needs to be provided), and justification of the choice of markers (if applicable).				
Use of reference standards	4	(a) Direct overlay of the chromatogram of the sample with that of an officially specified reference standard (if applicable).<br>(b) Chromatographic fingerprinting: Direct overlay of the chromatogram of the sample with that of official reference standards of the powdered plant material or the dry extract from the plant material.		✓		
Comparison of different extracts/samples of the same plants	5	(a) Direct comparison of the chromatographic/spectroscopic system and/or scoring system for "similarity" to be followed.			✓	

Note: Please also include here the following information about your submitted manuscript:

Name of the journal:	Journal of Ayurveda and Integrative Medicine
Date of the enquiry:	2021 November 25
Title of the manuscript:	mnifera supplementation on adult's cognition and mood
List of the authors:	D Hooper 1, S Hooper 1, K Conway 1, H A Hausenblas 2

[1] Please acknowledge/cite this as follows: Heinrich M, Jalil B, Abdel-Tawab M, Echeverria J, Kulić Ž, McGaw LJ, et al. Best Practice in the chemical characterisation of extracts used in pharmacological and toxicological research—The ConPhyMP—Guidelines. Frontiers in Pharmacology. 2022;13:953205. https://doi.org/10.3389/fphar.2022.953205

[2] We strongly recommend reading this checklist in conjuncton with ConPhyMP 2022 explanaton and elaboraton for important clarifications on all items. If relevant, we also recommend after reading Heinrich et al. (2020) Best practice in research—Overcoming common challenges in phytopharmacological research. Journal of Ethnopharmacology. 2020;246:112230. https://doi.org/10.1016/j.jep.2019.112230

ConPhyMP checklist of information for reporting plant material and its initial processing [1,2] (relevant for all studies on medicinal and food plants including extract types A, B, and C)

SECTION/TOPIC	ITEM NO.	CHECKLIST ITEM	YES	NO	NOT APPLICABLE	PAGE NO., IF ANY
Title and abstract	1	A clear and concise title including an informative abstract and balanced summary.	✓			
Description of the botanical drug and taxonomic authentication	2	Botanical or morphological authentication of the plant material (desirable is a combination with DNA barcoding, e.g., PCR, RFLP, genome sequencing) and the information must be included in a separate section of Material and Methods, if applicable, combined with the information required under item 3:	✓			
Description of the extract and extraction process	3	A separate section in Material and Methods, covers the relevant information on the material investigated, including the full species name(s), authorites and family; e.g. *Salvia miltorrhiza* Bunge [Lamiaceae; *Salviae miltorrhizae radix et rhizoma*], and on the processing and extraction of the crude drug including the traditional processing of the material used medicinally (fumigation, steaming, roasting, cooking, frying, etc.).		✓		
Documentation of the legal basis for collection and processing	4	Full compliance with the Nagoya protocol, CITES, and all associated treaties including phytosanitary regulations.			✓	
Description of product characteristics, in case of a finished (commercial) product	5	Information on the characteristics of the commercial products including batch number and date of production/best by information and regulatory status.	✓			

Note: Please also include here the following information about your submitted manuscript:

Name of the journal: Journal of Dietary Supplements

Date of the enquiry: 2024 Nov 5

Title of the manuscript: ts: Prospective, Randomized, Placebo-Controlled Study

List of the authors: ian Lopresti, Ravleen Suri, Neha Garg, Deepak Langade

Society for Medicinal Plant and Natural Product Research

Accessible at https://ga-online.org/conphymp

[1] Please acknowledge/cite this as follows: Heinrich M, Jalil B, Abdel-Tawab M, Echeverria J, Kulić Ž, McGaw LJ, et al. Best Practice in the chemical characterisation of extracts used in pharmacological and toxicological research—The ConPhyMP—Guidelines. Frontiers in Pharmacology. 2022;13:953205. https://doi.org/10.3389/fphar.2022.953205

[2] We strongly recommend reading this checklist in conjuncton with ConPhyMP 2022 explanaton and elaboraton for important clarifications on all items. If relevant, we also recommend after reading Heinrich et al. (2020) Best practice in research—Overcoming common challenges in phytopharmacological research. Journal of Ethnopharmacology. 2020;246:112230. https://doi.org/10.1016/j.jep.2019.112230

ConPhyMP checklist of items for conducting and reporting analytical methods[1,2] relevant for extract type A (for species or botanical drugs covered in a monograph in one of the national or regional pharmacopoeias)

SECTION/TOPIC	ITEM NO.	CHECKLIST ITEM	YES	NO	NOT APPLICABLE	PAGE NO., IF ANY
Type of extract	1	A – Confirm that the species or botanical drug under investigation is covered in a monograph in one of the national or regional pharmacopoeias.	✓			
Preferred/main methods for extract characterisation/ chemical analysis	2	Compliance with pharmacopoeial standards to be followed:				
		(a) The description of the active ingredients in the botanical drug (if known) or analytical marker compounds as defined.		✓		
		(b) An analysis as defined in the monograph is needed if the extract has not been supplied with a certificate.		✓		
		(c) If the preparation was purchased, the manufacturer and certificate of analysis need to be included.	✓			
		Including either the preferred or alternative approaches for characterisation:				
		(a) Triple chemical fingerprinting methods, each with one or more detection parameters.		✓		
		(b) Quantification of at least two marker compounds (unless this is not feasible, evidence needs to be provided), and justification of the choice of markers (if applicable).		✓		
Alternative methods for extract characterisation/chemical analysis	3	(a) Single chemical fingerprinting method with at least three different detection parameters (i.e., altered detection parameters, like TLC/ HPTLC with different staining reagents and/or UV excitation wave-lengths, HPLC-DAD/LCDAD with different wavelengths). The same applies to coupling MS or NMR to chromatographic techniques.		✓		
		(b) Quantification of at least two marker compounds (unless this is not feasible, evidence needs to be provided), and justification of the choice of markers (if applicable).		✓		
Use of reference standards	4	(a) Direct overlay of the chromatogram of the sample with that of an officially specified reference standard (if applicable). (b) Chromatographic fingerprinting: Direct overlay of the chromatogram of the sample with that of official reference standards of the powdered plant material or the dry extract from the plant material.		✓		
Comparison of different extracts/ samples of the same plants	5	(a) Direct comparison of the chromatographic/spectroscopic system and/or scoring system for "similarity" to be followed.			✓	

Note: Please also include here the following information about your submitted manuscript:

Name of the journal: Journal of Dietary Supplements

Date of the enquiry: 2024 Nov 5

Title of the manuscript: ts: Prospective, Randomized, Placebo-Controlled Study

List of the authors: ian Lopresti, Ravleen Suri, Neha Garg, Deepak Langade

[1] Please acknowledge/cite this as follows: Heinrich M, Jalil B, Abdel-Tawab M, Echeverria J, Kulić Ž, McGaw LJ, et al. Best Practice in the chemical characterisation of extracts used in pharmacological and toxicological research—The ConPhyMP—Guidelines. Frontiers in Pharmacology. 2022;13:953205. https://doi.org/10.3389/fphar.2022.953205

[2] We strongly recommend reading this checklist in conjuncton with ConPhyMP 2022 explanaton and elaboraton for important clarifications on all items. If relevant, we also recommend after reading Heinrich et al. (2020) Best practice in research—Overcoming common challenges in phytopharmacological research. Journal of Ethnopharmacology. 2020;246:112230. https://doi.org/10.1016/j.jep.2019.112230

ConPhyMP checklist of information for reporting plant material and its initial processing [1,2] (relevant for all studies on medicinal and food plants including extract types A, B, and C)

SECTION/TOPIC	ITEM NO.	CHECKLIST ITEM	YES	NO	NOT APPLICABLE	PAGE NO., IF ANY
Title and abstract	1	A clear and concise title including an informative abstract and balanced summary.	✓			
Description of the botanical drug and taxonomic authentication	2	Botanical or morphological authentication of the plant material (desirable is a combination with DNA barcoding, e.g., PCR, RFLP, genome sequencing) and the information must be included in a separate section of Material and Methods, if applicable, combined with the information required under item 3:		✓		
Description of the extract and extraction process	3	A separate section in Material and Methods, covers the relevant information on the material investigated, including the full species name(s), authorites and family; e.g. *Salvia miltorrhiza* Bunge [Lamiaceae; *Salviae miltorrhizae radix et rhizoma*], and on the processing and extraction of the crude drug including the traditional processing of the material used medicinally (fumigation, steaming, roasting, cooking, frying, etc.).		✓		
Documentation of the legal basis for collection and processing	4	Full compliance with the Nagoya protocol, CITES, and all associated treaties including phytosanitary regulations.			✓	
Description of product characteristics, in case of a finished (commercial) product	5	Information on the characteristics of the commercial products including batch number and date of production/best by information and regulatory status.	✓			

Note: Please also include here the following information about your submitted manuscript:

Name of the journal: l Journal of Environmental Research and Public Health

Date of the enquiry: 2022 Sep 20

Title of the manuscript: of Acute Ashwagandha Ingestion on Cognitive Function

List of the authors: owinski, Christopher J. Rasmussen, Richard B. Kreider

[1] Please acknowledge/cite this as follows: Heinrich M, Jalil B, Abdel-Tawab M, Echeverria J, Kulić Ž, McGaw LJ, et al. Best Practice in the chemical characterisation of extracts used in pharmacological and toxicological research—The ConPhyMP—Guidelines. Frontiers in Pharmacology. 2022;13:953205. https://doi.org/10.3389/fphar.2022.953205

[2] We strongly recommend reading this checklist in conjuncton with ConPhyMP 2022 explanaton and elaboraton for important clarifications on all items. If relevant, we also recommend after reading Heinrich et al. (2020) Best practice in research—Overcoming common challenges in phytopharmacological research. Journal of Ethnopharmacology. 2020;246:112230. https://doi.org/10.1016/j.jep.2019.112230

ConPhyMP checklist of items for conducting and reporting analytical methods[1,2] relevant for extract type A (for species or botanical drugs covered in a monograph in one of the national or regional pharmacopoeias)

SECTION/TOPIC	ITEM NO.	CHECKLIST ITEM	YES	NO	NOT APPLICABLE	PAGE NO., IF ANY
Type of extract	1	A – Confirm that the species or botanical drug under investigation is covered in a monograph in one of the national or regional pharmacopoeias.	✓			
Preferred/main methods for extract characterisation/ chemical analysis	2	Compliance with pharmacopoeial standards to be followed:				
		(a) The description of the active ingredients in the botanical drug (if known) or analytical marker compounds as defined.		✓		
		(b) An analysis as defined in the monograph is needed if the extract has not been supplied with a certificate.		✓		
		(c) If the preparation was purchased, the manufacturer and certificate of analysis need to be included.	✓			
		Including either the preferred or alternative approaches for characterisation:				
		(a) Triple chemical fingerprinting methods, each with one or more detection parameters.		✓		
		(b) Quantification of at least two marker compounds (unless this is not feasible, evidence needs to be provided), and justification of the choice of markers (if applicable).		✓		
Alternative methods for extract characterisation/chemical analysis	3	(a) Single chemical fingerprinting method with at least three different detection parameters (i.e., altered detection parameters, like TLC/ HPTLC with different staining reagents and/or UV excitation wave-lengths, HPLC-DAD/LCDAD with different wavelengths). The same applies to coupling MS or NMR to chromatographic techniques.		✓		
		(b) Quantification of at least two marker compounds (unless this is not feasible, evidence needs to be provided), and justification of the choice of markers (if applicable).		✓		
Use of reference standards	4	(a) Direct overlay of the chromatogram of the sample with that of an officially specified reference standard (if applicable). (b) Chromatographic fingerprinting: Direct overlay of the chromato-gram of the sample with that of official reference standards of the powdered plant material or the dry extract from the plant material.		✓		
Comparison of different extracts/ samples of the same plants	5	(a) Direct comparison of the chromatographic/spectroscopic system and/or scoring system for "similarity" to be followed.			✓	

Note: Please also include here the following information about your submitted manuscript:

Name of the journal:	l Journal of Environmental Research and Public Health
Date of the enquiry:	2022 Sep 20
Title of the manuscript:	of Acute Ashwagandha Ingestion on Cognitive Function
List of the authors:	owinski, Christopher J. Rasmussen, Richard B. Kreider

[1] Please acknowledge/cite this as follows: Heinrich M, Jalil B, Abdel-Tawab M, Echeverria J, Kulić Ž, McGaw LJ, et al. Best Practice in the chemical characterisation of extracts used in pharmacological and toxicological research—The ConPhyMP—Guidelines. Frontiers in Pharmacology. 2022;13:953205. https://doi.org/10.3389/fphar.2022.953205

[2] We strongly recommend reading this checklist in conjuncton with ConPhyMP 2022 explanaton and elaboraton for important clarifications on all items. If relevant, we also recommend after reading Heinrich et al. (2020) Best practice in research—Overcoming common challenges in phytopharmacological research. Journal of Ethnopharmacology. 2020;246:112230. https://doi.org/10.1016/j.jep.2019.112230

ConPhyMP checklist of information for reporting plant material and its initial processing [1,2] (relevant for all studies on medicinal and food plants including extract types A, B, and C)

SECTION/TOPIC	ITEM NO.	CHECKLIST ITEM	YES	NO	NOT APPLICABLE	PAGE NO., IF ANY
Title and abstract	1	A clear and concise title including an informative abstract and balanced summary.	✓			
Description of the botanical drug and taxonomic authentication	2	Botanical or morphological authentication of the plant material (desirable is a combination with DNA barcoding, e.g., PCR, RFLP, genome sequencing) and the information must be included in a separate section of Material and Methods, if applicable, combined with the information required under item 3:		✓		
Description of the extract and extraction process	3	A separate section in Material and Methods, covers the relevant information on the material investigated, including the full species name(s), authorites and family; e.g. *Salvia miltorrhiza* Bunge [Lamiaceae; *Salviae miltorrhizae radix et rhizoma*], and on the processing and extraction of the crude drug including the traditional processing of the material used medicinally (fumigation, steaming, roasting, cooking, frying, etc.).		✓		
Documentation of the legal basis for collection and processing	4	Full compliance with the Nagoya protocol, CITES, and all associated treaties including phytosanitary regulations.			✓	
Description of product characteristics, in case of a finished (commercial) product	5	Information on the characteristics of the commercial products including batch number and date of production/best by information and regulatory status.	✓			

Note: Please also include here the following information about your submitted manuscript:

Name of the journal: Medicine

Date of the enquiry: 2023 Sep 14

Title of the manuscript: a randomized, double-blind, placebo-controlled study

List of the authors: ammed Majeed, Kalyanam Nagabhushanam, Lakshmi Mundkur

Society for Medicinal Plant and Natural Product Research

Accessible at https://ga-online.org/conphymp

[1] Please acknowledge/cite this as follows: Heinrich M, Jalil B, Abdel-Tawab M, Echeverria J, Kulić Ž, McGaw LJ, et al. Best Practice in the chemical characterisation of extracts used in pharmacological and toxicological research—The ConPhyMP—Guidelines. Frontiers in Pharmacology. 2022;13:953205. https://doi.org/10.3389/fphar.2022.953205

[2] We strongly recommend reading this checklist in conjuncton with ConPhyMP 2022 explanaton and elaboraton for important clarifications on all items. If relevant, we also recommend after reading Heinrich et al. (2020) Best practice in research—Overcoming common challenges in phytopharmacological research. Journal of Ethnopharmacology. 2020;246:112230. https://doi.org/10.1016/j.jep.2019.112230

ConPhyMP checklist of items for conducting and reporting analytical methods[1,2] relevant for extract type A (for species or botanical drugs covered in a monograph in one of the national or regional pharmacopoeias)

SECTION/TOPIC	ITEM NO.	CHECKLIST ITEM	YES	NO	NOT APPLICABLE	PAGE NO., IF ANY
Type of extract	1	A – Confirm that the species or botanical drug under investigation is covered in a monograph in one of the national or regional pharmacopoeias.	✓			
Preferred/main methods for extract characterisation/ chemical analysis	2	Compliance with pharmacopoeial standards to be followed:				
		(a) The description of the active ingredients in the botanical drug (if known) or analytical marker compounds as defined.	✓			
		(b) An analysis as defined in the monograph is needed if the extract has not been supplied with a certificate.	✓			
		(c) If the preparation was purchased, the manufacturer and certificate of analysis need to be included.	✓			
		Including either the preferred or alternative approaches for characterisation:				
		(a) Triple chemical fingerprinting methods, each with one or more detection parameters.		✓		
		(b) Quantification of at least two marker compounds (unless this is not feasible, evidence needs to be provided), and justification of the choice of markers (if applicable).		✓		
Alternative methods for extract characterisation/chemical analysis	3	(a) Single chemical fingerprinting method with at least three different detection parameters (i.e., altered detection parameters, like TLC/ HPTLC with different staining reagents and/or UV excitation wave-lengths, HPLC-DAD/LCDAD with different wavelengths). The same applies to coupling MS or NMR to chromatographic techniques.		✓		
		(b) Quantification of at least two marker compounds (unless this is not feasible, evidence needs to be provided), and justification of the choice of markers (if applicable).		✓		
Use of reference standards	4	(a) Direct overlay of the chromatogram of the sample with that of an officially specified reference standard (if applicable).		✓		
		(b) Chromatographic fingerprinting: Direct overlay of the chromato-gram of the sample with that of official reference standards of the powdered plant material or the dry extract from the plant material.				
Comparison of different extracts/ samples of the same plants	5	(a) Direct comparison of the chromatographic/spectroscopic system and/or scoring system for "similarity" to be followed.			✓	

Note: Please also include here the following information about your submitted manuscript:

Name of the journal: Medicine

Date of the enquiry: 2023 Sep 14

Title of the manuscript: a randomized, double-blind, placebo-controlled study

List of the authors: ammed Majeed, Kalyanam Nagabhushanam, Lakshmi Mundkur

[1] Please acknowledge/cite this as follows: Heinrich M, Jalil B, Abdel-Tawab M, Echeverria J, Kulić Ž, McGaw LJ, et al. Best Practice in the chemical characterisation of extracts used in pharmacological and toxicological research—The ConPhyMP—Guidelines. Frontiers in Pharmacology. 2022;13:953205. https://doi.org/10.3389/fphar.2022.953205

[2] We strongly recommend reading this checklist in conjuncton with ConPhyMP 2022 explanaton and elaboraton for important clarifications on all items. If relevant, we also recommend after reading Heinrich et al. (2020) Best practice in research—Overcoming common challenges in phytopharmacological research. Journal of Ethnopharmacology. 2020;246:112230. https://doi.org/10.1016/j.jep.2019.112230

ConPhyMP checklist of information for reporting plant material and its initial processing [1,2] (relevant for all studies on medicinal and food plants including extract types A, B, and C)

SECTION/TOPIC	ITEM NO.	CHECKLIST ITEM	YES	NO	NOT APPLICABLE	PAGE NO., IF ANY
Title and abstract	1	A clear and concise title including an informative abstract and balanced summary.	✓			
Description of the botanical drug and taxonomic authentication	2	Botanical or morphological authentication of the plant material (desirable is a combination with DNA barcoding, e.g., PCR, RFLP, genome sequencing) and the information must be included in a separate section of Material and Methods, if applicable, combined with the information required under item 3:		✓		
Description of the extract and extraction process	3	A separate section in Material and Methods, covers the relevant information on the material investigated, including the full species name(s), authorites and family; e.g. *Salvia miltorrhiza* Bunge [Lamiaceae; *Salviae miltorrhizae radix et rhizoma*], and on the processing and extraction of the crude drug including the traditional processing of the material used medicinally (fumigation, steaming, roasting, cooking, frying, etc.).	✓			
Documentation of the legal basis for collection and processing	4	Full compliance with the Nagoya protocol, CITES, and all associated treaties including phytosanitary regulations.			✓	
Description of product characteristics, in case of a finished (commercial) product	5	Information on the characteristics of the commercial products including batch number and date of production/best by information and regulatory status.	✓			

Note: Please also include here the following information about your submitted manuscript:

Name of the journal:	Nutrients
Date of the enquiry:	2018 Nov 20
Title of the manuscript:	gth Training Adaptations and Recovery: The STAR Trial
List of the authors:	rock, Bill J. Raub, Chad M. Kerksick, Hector L. Lopez

[1] Please acknowledge/cite this as follows: Heinrich M, Jalil B, Abdel-Tawab M, Echeverria J, Kulić Ž, McGaw LJ, et al. Best Practice in the chemical characterisation of extracts used in pharmacological and toxicological research—The ConPhyMP—Guidelines. Frontiers in Pharmacology. 2022;13:953205. https://doi.org/10.3389/fphar.2022.953205

[2] We strongly recommend reading this checklist in conjuncton with ConPhyMP 2022 explanaton and elaboraton for important clarifications on all items. If relevant, we also recommend after reading Heinrich et al. (2020) Best practice in research—Overcoming common challenges in phytopharmacological research. Journal of Ethnopharmacology. 2020;246:112230. https://doi.org/10.1016/j.jep.2019.112230

ConPhyMP checklist of items for conducting and reporting analytical methods[1,2] relevant for extract type A (for species or botanical drugs covered in a monograph in one of the national or regional pharmacopoeias)

SECTION/TOPIC	ITEM NO.	CHECKLIST ITEM	YES	NO	NOT APPLICABLE	PAGE NO., IF ANY
Type of extract	1	A – Confirm that the species or botanical drug under investigation is covered in a monograph in one of the national or regional pharmacopoeias.	✓			
Preferred/main methods for extract characterisation/ chemical analysis	2	Compliance with pharmacopoeial standards to be followed:				
		(a) The description of the active ingredients in the botanical drug (if known) or analytical marker compounds as defined.	✓			
		(b) An analysis as defined in the monograph is needed if the extract has not been supplied with a certificate.	✓			
		(c) If the preparation was purchased, the manufacturer and certificate of analysis need to be included.	✓			
		Including either the preferred or alternative approaches for characterisation:				
		(a) Triple chemical fingerprinting methods, each with one or more detection parameters.		✓		
		(b) Quantification of at least two marker compounds (unless this is not feasible, evidence needs to be provided), and justification of the choice of markers (if applicable).		✓		
Alternative methods for extract characterisation/chemical analysis	3	(a) Single chemical fingerprinting method with at least three different detection parameters (i.e., altered detection parameters, like TLC/ HPTLC with different staining reagents and/or UV excitation wavelengths, HPLC-DAD/LCDAD with different wavelengths). The same applies to coupling MS or NMR to chromatographic techniques.		✓		
		(b) Quantification of at least two marker compounds (unless this is not feasible, evidence needs to be provided), and justification of the choice of markers (if applicable).		✓		
Use of reference standards	4	(a) Direct overlay of the chromatogram of the sample with that of an officially specified reference standard (if applicable).		✓		
		(b) Chromatographic fingerprinting: Direct overlay of the chromatogram of the sample with that of official reference standards of the powdered plant material or the dry extract from the plant material.				
Comparison of different extracts/ samples of the same plants	5	(a) Direct comparison of the chromatographic/spectroscopic system and/or scoring system for "similarity" to be followed.			✓	

Note: Please also include here the following information about your submitted manuscript:

Name of the journal:	Nutrients
Date of the enquiry:	2018 Nov 20
Title of the manuscript:	gth Training Adaptations and Recovery: The STAR Trial
List of the authors:	rock, Bill J. Raub, Chad M. Kerksick, Hector L. Lopez

[1] Please acknowledge/cite this as follows: Heinrich M, Jalil B, Abdel-Tawab M, Echeverria J, Kulić Ž, McGaw LJ, et al. Best Practice in the chemical characterisation of extracts used in pharmacological and toxicological research—The ConPhyMP—Guidelines. Frontiers in Pharmacology. 2022;13:953205. https://doi.org/10.3389/fphar.2022.953205

[2] We strongly recommend reading this checklist in conjuncton with ConPhyMP 2022 explanaton and elaboraton for important clarifications on all items. If relevant, we also recommend after reading Heinrich et al. (2020) Best practice in research—Overcoming common challenges in phytopharmacological research. Journal of Ethnopharmacology. 2020;246:112230. https://doi.org/10.1016/j.jep.2019.112230

ConPhyMP checklist of information for reporting plant material and its initial processing [1,2] (relevant for all studies on medicinal and food plants including extract types A, B, and C)

SECTION/TOPIC	ITEM NO.	CHECKLIST ITEM	YES	NO	NOT APPLICABLE	PAGE NO., IF ANY
Title and abstract	1	A clear and concise title including an informative abstract and balanced summary.	✓			
Description of the botanical drug and taxonomic authentication	2	Botanical or morphological authentication of the plant material (desirable is a combination with DNA barcoding, e.g., PCR, RFLP, genome sequencing) and the information must be included in a separate section of Material and Methods, if applicable, combined with the information required under item 3:		✓		
Description of the extract and extraction process	3	A separate section in Material and Methods, covers the relevant information on the material investigated, including the full species name(s), authorites and family; e.g. *Salvia miltorrhiza* Bunge [Lamiaceae; *Salviae miltorrhizae radix et rhizoma*], and on the processing and extraction of the crude drug including the traditional processing of the material used medicinally (fumigation, steaming, roasting, cooking, frying, etc.).	✓			
Documentation of the legal basis for collection and processing	4	Full compliance with the Nagoya protocol, CITES, and all associated treaties including phytosanitary regulations.			✓	
Description of product characteristics, in case of a finished (commercial) product	5	Information on the characteristics of the commercial products including batch number and date of production/best by information and regulatory status.	✓			

Note: Please also include here the following information about your submitted manuscript:

Name of the journal: Pharmacognosy Research

Date of the enquiry: 2014 Jan

Title of the manuscript: psychomotor performance in healthy human participants

List of the authors: Usharani Pingali, Raveendranadh Pilli, Nishat Fatima

[1] Please acknowledge/cite this as follows: Heinrich M, Jalil B, Abdel-Tawab M, Echeverria J, Kulić Ž, McGaw LJ, et al. Best Practice in the chemical characterisation of extracts used in pharmacological and toxicological research—The ConPhyMP—Guidelines. Frontiers in Pharmacology. 2022;13:953205. https://doi.org/10.3389/fphar.2022.953205

[2] We strongly recommend reading this checklist in conjuncton with ConPhyMP 2022 explanaton and elaboraton for important clarifications on all items. If relevant, we also recommend after reading Heinrich et al. (2020) Best practice in research—Overcoming common challenges in phytopharmacological research. Journal of Ethnopharmacology. 2020;246:112230. https://doi.org/10.1016/j.jep.2019.112230

ConPhyMP checklist of items for conducting and reporting analytical methods[1,2] relevant for extract type A (for species or botanical drugs covered in a monograph in one of the national or regional pharmacopoeias)

SECTION/TOPIC	ITEM NO.	CHECKLIST ITEM	YES	NO	NOT APPLICABLE	PAGE NO., IF ANY
Type of extract	1	A – Confirm that the species or botanical drug under investigation is covered in a monograph in one of the national or regional pharmacopoeias.	✓			
Preferred/main methods for extract characterisation/ chemical analysis	2	Compliance with pharmacopoeial standards to be followed:				
		(a) The description of the active ingredients in the botanical drug (if known) or analytical marker compounds as defined.	✓			
		(b) An analysis as defined in the monograph is needed if the extract has not been supplied with a certificate.		✓		
		(c) If the preparation was purchased, the manufacturer and certificate of analysis need to be included.	✓			
		Including either the preferred or alternative approaches for characterisation:				
		(a) Triple chemical fingerprinting methods, each with one or more detection parameters.		✓		
		(b) Quantification of at least two marker compounds (unless this is not feasible, evidence needs to be provided), and justification of the choice of markers (if applicable).	✓			
Alternative methods for extract characterisation/chemical analysis	3	(a) Single chemical fingerprinting method with at least three different detection parameters (i.e., altered detection parameters, like TLC/ HPTLC with different staining reagents and/or UV excitation wave-lengths, HPLC-DAD/LCDAD with different wavelengths). The same applies to coupling MS or NMR to chromatographic techniques.		✓		
		(b) Quantification of at least two marker compounds (unless this is not feasible, evidence needs to be provided), and justification of the choice of markers (if applicable).	✓			
Use of reference standards	4	(a) Direct overlay of the chromatogram of the sample with that of an officially specified reference standard (if applicable).		✓		
		(b) Chromatographic fingerprinting: Direct overlay of the chromatogram of the sample with that of official reference standards of the powdered plant material or the dry extract from the plant material.				
Comparison of different extracts/ samples of the same plants	5	(a) Direct comparison of the chromatographic/spectroscopic system and/or scoring system for "similarity" to be followed.			✓	

Note: Please also include here the following information about your submitted manuscript:

Name of the journal: Pharmacognosy Research

Date of the enquiry: 2014 Jan

Title of the manuscript: psychomotor performance in healthy human participants

List of the authors: Usharani Pingali, Raveendranadh Pilli, Nishat Fatima

[1] Please acknowledge/cite this as follows: Heinrich M, Jalil B, Abdel-Tawab M, Echeverria J, Kulić Ž, McGaw LJ, et al. Best Practice in the chemical characterisation of extracts used in pharmacological and toxicological research—The ConPhyMP—Guidelines. Frontiers in Pharmacology. 2022;13:953205. https://doi.org/10.3389/fphar.2022.953205

[2] We strongly recommend reading this checklist in conjuncton with ConPhyMP 2022 explanaton and elaboraton for important clarifications on all items. If relevant, we also recommend after reading Heinrich et al. (2020) Best practice in research—Overcoming common challenges in phytopharmacological research. Journal of Ethnopharmacology. 2020;246:112230. https://doi.org/10.1016/j.jep.2019.112230

ConPhyMP checklist of information for reporting plant material and its initial processing [1,2] (relevant for all studies on medicinal and food plants including extract types A, B, and C)

SECTION/TOPIC	ITEM NO.	CHECKLIST ITEM	YES	NO	NOT APPLICABLE	PAGE NO., IF ANY
Title and abstract	1	A clear and concise title including an informative abstract and balanced summary.	✓			
Description of the botanical drug and taxonomic authentication	2	Botanical or morphological authentication of the plant material (desirable is a combination with DNA barcoding, e.g., PCR, RFLP, genome sequencing) and the information must be included in a separate section of Material and Methods, if applicable, combined with the information required under item 3:		✓		
Description of the extract and extraction process	3	A separate section in Material and Methods, covers the relevant information on the material investigated, including the full species name(s), authorites and family; e.g. *Salvia miltorrhiza* Bunge [Lamiaceae; *Salviae miltorrhizae radix et rhizoma*], and on the processing and extraction of the crude drug including the traditional processing of the material used medicinally (fumigation, steaming, roasting, cooking, frying, etc.).	✓			
Documentation of the legal basis for collection and processing	4	Full compliance with the Nagoya protocol, CITES, and all associated treaties including phytosanitary regulations.			✓	
Description of product characteristics, in case of a finished (commercial) product	5	Information on the characteristics of the commercial products including batch number and date of production/best by information and regulatory status.	✓			

Note: Please also include here the following information about your submitted manuscript:

Name of the journal: Evidence-Based Complementary and Alternative Medicine

Date of the enquiry: 2013 Nov 28

Title of the manuscript: hania somnifera) in Oligospermic Males: A Pilot Study

List of the authors: ongre, Pradnya Aptikar, Madhura Kulkarni, Atul Dongre

[1] Please acknowledge/cite this as follows: Heinrich M, Jalil B, Abdel-Tawab M, Echeverria J, Kulić Ž, McGaw LJ, et al. Best Practice in the chemical characterisation of extracts used in pharmacological and toxicological research—The ConPhyMP—Guidelines. Frontiers in Pharmacology. 2022;13:953205. https://doi.org/10.3389/fphar.2022.953205

[2] We strongly recommend reading this checklist in conjuncton with ConPhyMP 2022 explanaton and elaboraton for important clarifications on all items. If relevant, we also recommend after reading Heinrich et al. (2020) Best practice in research—Overcoming common challenges in phytopharmacological research. Journal of Ethnopharmacology. 2020;246:112230. https://doi.org/10.1016/j.jep.2019.112230

ConPhyMP checklist of items for conducting and reporting analytical methods[1,2] relevant for extract type A (for species or botanical drugs covered in a monograph in one of the national or regional pharmacopoeias)

SECTION/TOPIC	ITEM NO.	CHECKLIST ITEM	YES	NO	NOT APPLICABLE	PAGE NO., IF ANY
Type of extract	1	A – Confirm that the species or botanical drug under investigation is covered in a monograph in one of the national or regional pharmacopoeias.	✓			
Preferred/main methods for extract characterisation/ chemical analysis	2	Compliance with pharmacopoeial standards to be followed:				
		(a) The description of the active ingredients in the botanical drug (if known) or analytical marker compounds as defined.	✓			
		(b) An analysis as defined in the monograph is needed if the extract has not been supplied with a certificate.		✓		
		(c) If the preparation was purchased, the manufacturer and certificate of analysis need to be included.	✓			
		Including either the preferred or alternative approaches for characterisation:				
		(a) Triple chemical fingerprinting methods, each with one or more detection parameters.		✓		
		(b) Quantification of at least two marker compounds (unless this is not feasible, evidence needs to be provided), and justification of the choice of markers (if applicable).		✓		
Alternative methods for extract characterisation/chemical analysis	3	(a) Single chemical fingerprinting method with at least three different detection parameters (i.e., altered detection parameters, like TLC/HPTLC with different staining reagents and/or UV excitation wave-lengths, HPLC-DAD/LCDAD with different wavelengths). The same applies to coupling MS or NMR to chromatographic techniques.		✓		
		(b) Quantification of at least two marker compounds (unless this is not feasible, evidence needs to be provided), and justification of the choice of markers (if applicable).		✓		
Use of reference standards	4	(a) Direct overlay of the chromatogram of the sample with that of an officially specified reference standard (if applicable).		✓		
		(b) Chromatographic fingerprinting: Direct overlay of the chromato-gram of the sample with that of official reference standards of the powdered plant material or the dry extract from the plant material.				
Comparison of different extracts/ samples of the same plants	5	(a) Direct comparison of the chromatographic/spectroscopic system and/or scoring system for "similarity" to be followed.			✓	

Note: Please also include here the following information about your submitted manuscript:

Name of the journal: Evidence-Based Complementary and Alternative Medicine

Date of the enquiry: 2013 Nov 28

Title of the manuscript: hania somnifera) in Oligospermic Males: A Pilot Study

List of the authors: ongre, Pradnya Aptikar, Madhura Kulkarni, Atul Dongre

[1] Please acknowledge/cite this as follows: Heinrich M, Jalil B, Abdel-Tawab M, Echeverria J, Kulić Ž, McGaw LJ, et al. Best Practice in the chemical characterisation of extracts used in pharmacological and toxicological research—The ConPhyMP—Guidelines. Frontiers in Pharmacology. 2022;13:953205. https://doi.org/10.3389/fphar.2022.953205

[2] We strongly recommend reading this checklist in conjuncton with ConPhyMP 2022 explanaton and elaboraton for important clarifications on all items. If relevant, we also recommend after reading Heinrich et al. (2020) Best practice in research—Overcoming common challenges in phytopharmacological research. Journal of Ethnopharmacology. 2020;246:112230. https://doi.org/10.1016/j.jep.2019.112230

ConPhyMP checklist of information for reporting plant material and its initial processing [1,2] (relevant for all studies on medicinal and food plants including extract types A, B, and C)

SECTION/TOPIC	ITEM NO.	CHECKLIST ITEM	YES	NO	NOT APPLICABLE	PAGE NO., IF ANY
Title and abstract	1	A clear and concise title including an informative abstract and balanced summary.	✓			
Description of the botanical drug and taxonomic authentication	2	Botanical or morphological authentication of the plant material (desirable is a combination with DNA barcoding, e.g., PCR, RFLP, genome sequencing) and the information must be included in a separate section of Material and Methods, if applicable, combined with the information required under item 3:		✓		
Description of the extract and extraction process	3	A separate section in Material and Methods, covers the relevant information on the material investigated, including the full species name(s), authorites and family; e.g. *Salvia miltorrhiza* Bunge [Lamiaceae; *Salviae miltorrhizae radix et rhizoma*], and on the processing and extraction of the crude drug including the traditional processing of the material used medicinally (fumigation, steaming, roasting, cooking, frying, etc.).		✓		
Documentation of the legal basis for collection and processing	4	Full compliance with the Nagoya protocol, CITES, and all associated treaties including phytosanitary regulations.			✓	
Description of product characteristics, in case of a finished (commercial) product	5	Information on the characteristics of the commercial products including batch number and date of production/best by information and regulatory status.	✓			

Note: Please also include here the following information about your submitted manuscript:

Name of the journal:	Journal of Obstetrics and Gynaecology Research
Date of the enquiry:	2021 Sep 22
Title of the manuscript:	A randomized, double-blind, placebo-controlled study
List of the authors:	ya, Vaishali Thakare, Sanjaya Chauhan, Deepak Langade

[1] Please acknowledge/cite this as follows: Heinrich M, Jalil B, Abdel-Tawab M, Echeverria J, Kulić Ž, McGaw LJ, et al. Best Practice in the chemical characterisation of extracts used in pharmacological and toxicological research—The ConPhyMP—Guidelines. Frontiers in Pharmacology. 2022;13:953205. https://doi.org/10.3389/fphar.2022.953205

[2] We strongly recommend reading this checklist in conjuncton with ConPhyMP 2022 explanaton and elaboraton for important clarifications on all items. If relevant, we also recommend after reading Heinrich et al. (2020) Best practice in research—Overcoming common challenges in phytopharmacological research. Journal of Ethnopharmacology. 2020;246:112230. https://doi.org/10.1016/j.jep.2019.112230

ConPhyMP checklist of items for conducting and reporting analytical methods[1,2] relevant for extract type A (for species or botanical drugs covered in a monograph in one of the national or regional pharmacopoeias)

SECTION/TOPIC	ITEM NO.	CHECKLIST ITEM	YES	NO	NOT APPLICABLE	PAGE NO., IF ANY
Type of extract	1	A – Confirm that the species or botanical drug under investigation is covered in a monograph in one of the national or regional pharmacopoeias.	✓			
Preferred/main methods for extract characterisation/ chemical analysis	2	Compliance with pharmacopoeial standards to be followed:				
		(a) The description of the active ingredients in the botanical drug (if known) or analytical marker compounds as defined.	✓			
		(b) An analysis as defined in the monograph is needed if the extract has not been supplied with a certificate.		✓		
		(c) If the preparation was purchased, the manufacturer and certificate of analysis need to be included.	✓			
		Including either the preferred or alternative approaches for characterisation:				
		(a) Triple chemical fingerprinting methods, each with one or more detection parameters.		✓		
		(b) Quantification of at least two marker compounds (unless this is not feasible, evidence needs to be provided), and justification of the choice of markers (if applicable).		✓		
Alternative methods for extract characterisation/chemical analysis	3	(a) Single chemical fingerprinting method with at least three different detection parameters (i.e., altered detection parameters, like TLC/ HPTLC with different staining reagents and/or UV excitation wave-lengths, HPLC-DAD/LCDAD with different wavelengths). The same applies to coupling MS or NMR to chromatographic techniques.		✓		
		(b) Quantification of at least two marker compounds (unless this is not feasible, evidence needs to be provided), and justification of the choice of markers (if applicable).		✓		
Use of reference standards	4	(a) Direct overlay of the chromatogram of the sample with that of an officially specified reference standard (if applicable).<br>(b) Chromatographic fingerprinting: Direct overlay of the chromato-gram of the sample with that of official reference standards of the powdered plant material or the dry extract from the plant material.		✓		
Comparison of different extracts/ samples of the same plants	5	(a) Direct comparison of the chromatographic/spectroscopic system and/or scoring system for "similarity" to be followed.			✓	

Note: Please also include here the following information about your submitted manuscript:

Name of the journal: Journal of Obstetrics and Gynaecology Research

Date of the enquiry: 2021 Sep 22

Title of the manuscript: A randomized, double-blind, placebo-controlled study

List of the authors: ya, Vaishali Thakare, Sanjaya Chauhan, Deepak Langade

[1] Please acknowledge/cite this as follows: Heinrich M, Jalil B, Abdel-Tawab M, Echeverria J, Kulić Ž, McGaw LJ, et al. Best Practice in the chemical characterisation of extracts used in pharmacological and toxicological research—The ConPhyMP—Guidelines. Frontiers in Pharmacology. 2022;13:953205. https://doi.org/10.3389/fphar.2022.953205

[2] We strongly recommend reading this checklist in conjuncton with ConPhyMP 2022 explanaton and elaboraton for important clarifications on all items. If relevant, we also recommend after reading Heinrich et al. (2020) Best practice in research—Overcoming common challenges in phytopharmacological research. Journal of Ethnopharmacology. 2020;246:112230. https://doi.org/10.1016/j.jep.2019.112230
